# Intense pulsed light improves signs and symptoms of dry eye disease due to meibomian gland dysfunction: A randomized controlled study

**DOI:** 10.1371/journal.pone.0270268

**Published:** 2022-06-23

**Authors:** Rolando Toyos, Neel R. Desai, Melissa Toyos, Steven J. Dell

**Affiliations:** 1 Department of Ophthalmology, Toyos Clinic, Germantown, Tennessee, United States of America; 2 Eye Institute of West Florida, Largo, Florida, United States of America; 3 Dell Laser Consultants, Austin, Texas, United States of America; Prince Sattam Bin Abdulaziz University, College of Applied Medical Sciences, SAUDI ARABIA

## Abstract

**Purpose:**

To compare the safety and efficacy of intense pulsed light (IPL) followed by meibomian gland expression (MGX), against monotherapy of MGX.

**Methods:**

Patients with moderate to severe meibomian gland dysfunction (MGD) were 1:1 randomized to 4 sessions of intense pulse light + MGX at 2-week intervals, or 4 sessions of Sham + MGX at 2-week intervals. Both patients and examiners were blinded to the allocation. Outcome measures, evaluated at the baseline (BL) and at a follow-up (FU) conducted 4 weeks after the last IPL session, included fluorescein tear breakup time (TBUT) as the primary outcome measure, OSDI (Ocular Surface Disease Index) questionnaire, Eye Dryness Score (EDS, a visual analog scale (VAS)-based questionnaire), Meibomian gland score (MGS, a score of meibum expressibility and quality in 15 glands on the lower eyelid), daily use of artificial tears, and daily use of warm compresses. In addition, during each treatment session, the number of expressible glands was counted in both eyelids, the predominant quality of meibum was estimated in both eyelids, and the level of pain/discomfort due to MGX and IPL was recorded.

**Results:**

TBUT increased from 3.8±0.2 (μ±standard error of mean (SEM)) to 4.5±0.3 seconds in the control arm, and from 4.0±0.2 to 6.0±0.3 in the study arm. The difference between arms was statistically significant (P < .01). Other signs/symptoms which improved in both arms but were greater in the study arm included MGS (P < .001), EDS (P < .01), the number of expressible glands in the lower eyelids (P < .0001) and upper eyelid (P < .0001), the predominant meibum quality in the lower eyelid (P < .0001) and upper eyelid (P < .0001), and the level of pain due to MGX (P < .0001). Outcome measures which improved in both arms with no significant differences between the two were OSDI (P = .9984), and the daily use of artificial tears (P = .8216). Meibography, daily use of warm compresses, and severity of skin rosacea did not show statistically significant changes in either arm. No serious adverse events were observed. There was a slight tendency for more adverse events in the control group (P = 0.06).

**Conclusions:**

The results of this study suggest that, in patients with moderate to severe symptoms, combination therapy of intense pulse light (IPL) and meibomian gland expression (MGX) could be a safe and useful approach for improving signs of dry eye disease (DED) due to meibomian gland dysfunction (MGD). Future studies are needed to elucidate if and how such improvements can be generalized to different severity levels of MGD.

## Introduction

### Background

Dry eye disease (DED) is a multifactorial disease of the ocular surface, characterized by a loss of homeostasis of the tear film, tear film instability and ocular surface inflammation [1, 2]. The prevalence of DED is between 5% and 50%, depending on geographical region [3]. The condition occurs in two main forms, aqueous-deficiency and evaporative, although both types often co-exist [4]. The major cause of evaporative dry eye disease is meibomian gland dysfunction (MGD), defined by the international workshop on MGD as “a chronic, diffuse abnormality of the meibomian glands, commonly characterized by terminal duct obstruction and/or qualitative/quantitative changes in the glandular secretion” [5]. MGD leads to poor quality of the meibum, destabilization of the tear film, exposure of the ocular surface and, eventually, the development of dry eye symptoms [6]. It was estimated that between 60% [7] and 86% [8] of DED cases are due to MGD. According to Tear Film & Ocular Society’s Dry Eye Workshop II (TFOS DEWS II), over the age of 40 the prevalence rate of MGD ranges between 38% to 68% [3].

Many treatments for MGD include lid hygiene [6], thermal pulsation [9], artificial tear substitutes [10], artificial lubricants [6], topical or systemic antibiotics [10], FDA-approved anti-inflammatory medicines like cyclosporine [11], autologous serum eye drops [12], immunosuppressant agents [6], Lymphocyte function-associated antigen-1antagonists [13], and meibomian gland expression [14–16].

Another therapeutic approach, which has gained popularity in the past 5 years, is administration of intense pulse light (IPL) to the skin of the periocular area. IPL technology consists of brief pulses of non-coherent and polychromatic light, with wavelengths ranging from 500 to 1200 nm. IPL was found to be useful in a range of dermatological applications, including capillary and venous malformations [17], telangiectasia [18], and erythema of rosacea [19]. The latter condition is especially relevant for dry eye disease, as it is estimated that about 80% of patients with skin rosacea suffer from MGD [20]. It is therefore reasonable to expect that IPL, which is extremely effective for improving rosacea, could be useful for management of MGD as well. Indeed, although the mechanism of action is still not well understood [21], since the pioneering work of Toyos and colleagues [22] a large number of studies have indicated that IPL can reduce both signs and symptoms of dry eye [23–26]. In its staged management algorithm, the TFOS DEWS II recommended this technology as a second step for treatment of DED, after lid hygiene and ocular lubricants of various types [10]. In 2020, Cote and colleagues performed a systematic review of the clinical literature and concluded that, due to the scarcity of randomized controlled trials (RCTs), the therapeutic value of IPL is still uncertain [27]. These authors also pointed out that the safety profile of IPL was not sufficiently reported.

### Objectives

The purpose of the current prospective multi-center RCT study is to further demonstrate the merits of IPL treatment for DED due to MGD, in a multi-site study performed on a North American population. The main objective was to demonstrate that IPL combined with meibomian gland expression is superior to meibomian gland expression alone, in terms of improvement of signs and symptoms of DED due to MGD. The null hypothesis was that there is no statistically significant difference between the change in tear-break up time in patients treated with IPL combined with MGX, and patients treated with MGX alone.

## Materials and methods

This research was approved by an Institutional Review Board (Sterling IRB, # 6051), and registered in ClinicalTrials.gov (NTC03396913).

### Trial design

This was a prospective, interventional, multi-site, parallel-group, two-arms, randomized, active-controlled with a 1:1 allocation ratio. The trial is set to assess the superiority of the study arm versus the control arm. The study was conducted in the Unites States (3 sites).

### Changes to trial design

In the original design, the sample size was planned for a power of 80%. The primary outcome measure was to be collected in both eyes, but the primary study hypothesis was to be evaluated for the study eye only, where the study eye was defined as the eye with the worst primary outcome measure at baseline (BL). After study commencement (but before any data was unmasked), it was found that 8 patients in one site were not treated in accordance with the treatment protocol. The study was paused in this site, until the treating physician in this site was re-trained to conform with the treatment protocol. To maintain a power of 80% the study statistician recommended to increase the sample size (see below). Following this recommendation, the protocol was amended and approved by the IRB.

### Participants

Eligible participants were adults aged 22 to 85 years of age with signs and symptoms of dry eye disease due to MGD, who met all inclusion and exclusion criteria. The inclusion criteria included patients with a tear break-up time (TBUT) ≤ 7 seconds in the study eye; patients with a meibomian gland secretion (MGS) ≤ 12 in the study eye (where MGS is a score evaluating the quality of meibum along the lower eyelid, as described by Lane and colleagues [9]: sum of 5 nasal + 5 central + 5 temporal meibomian glands along the lower eyelid, where each gland is graded 0 if blocked, or 1, 2, 3 if expressing an inspissated meibum, cloudy liquid meibum, a clear liquid meibum, respectively); patients with at least 5 non-atrophied meibomian glands in the lower eyelid of the study eye; and patients with an OSDI questionnaire score ≥ 23 (moderate to severe symptoms of dry eye, as defined by Miller et al., 2010 [28]). The main exclusion criteria included Fitzpatrick skin type V or VI; use of prescription eye drops within 7 days (excluding artificial tears or glaucoma drops) of recruitment; facial IPL treatment within the past 12 months; any thermal treatment of the eyelids or meibomian gland expression within the past 6 months; ocular surface and eyelid abnormalities, any systemic condition that may cause dry eye; use of photosensitive drugs within the past 3 months; pre-cancerous lesions, skin cancer or pigmented lesions within the treatment area; over exposure to sun within the past 1 month; ocular infections within the past 6 months; uncontrolled infections or immunosuppressive diseases; and unwillingness or inability to abstain from the use of medications known to cause dryness. An informed consent was obtained from all subjects enrolled in the study.

### Study settings

The study took place from January 2018 to July 2019, at 3 clinics in the USA (Dell Laser Consultants in Austin, Texas; Toyos Clinic in Nashville, Tennessee; Eye Institute of West Florida in Largo, Florida).

### Interventions

Patients were randomly assigned to receive IPL treatment followed by meibomian gland expression (the study arm) or sham IPL followed by meibomian gland expression (the control arm). Each patient underwent a series of 4 treatment sessions, 2 weeks apart. In each session, the eyes of the patient were occluded with eye protection (adhesive eye patches + Lumenis opaque goggles). In the study arm, IPL was generated by a Lumenis M22 system, with a 560 nm or 590 nm cut-off filter that blocked all wavelengths below 560 nm or 590 nm, respectively. In the control arm, IPL was generated by the same system, but all light signals were blocked with an aluminum plate instead of the 560/590 cut-off filter. based on the double-pass protocol described in a previous publication by Toyos and colleagues [22]. The treatment area included the malar region (from tragus to tragus, including the nose) and the peri-ocular area up to the lower edge of the eye protection, positioned along the lower lid margin inferior to the lash line. IPL treatment was administered in two passes. For patients with the study arm, fluence was adjusted based on the Fitzpatrick skin type (from 11 to 15 J/cm^2^, for Fitzpatrick skin types of IV to I, respectively). In all patients, a single follow-up (FU) session was scheduled 4 weeks after the fourth treatment session.

Participants were allowed to continue using artificial tears or warm compresses during the study.

### Outcomes

The primary endpoint was the change in tear break-up time (TBUT). Measurement of TBUT followed the same protocol in all sites: a FUL-GLO^®^ fluorescein sodium ophthalmic strip (0.6 mg) was applied to the inferior tarsal conjunctiva. The subject was asked to blink a few times to distribute the dye over the ocular surface. Once positioned at the slit lamp, the subject closed his/her eyelids completely. The examiner viewed the eye of the subject through a slit lamp using broad beam cobalt blue illumination and a yellow barrier filter. Then, the subject was asked to open his/her eyelids without blinking. A stopwatch was started as soon as the subject opened the eyelids, and was stopped at the first sign of breakup (first dark spot or discontinuity in the precorneal fluorescein-stained tear layer). For each eye, 3 consecutive readings were taken, and the average value was recorded. The change in TBUT, (ΔTBUT, was defined as the difference in the value of the outcome measure at baseline and at the follow-up (TBUT(FU)—TBUT(BL)).

Secondary endpoints included the change in OSDI (a validated questionnaire for self-assessment of symptoms, ranging from 0–100, where 0 indicated no symptoms and 100 was consistent with the most severe and frequent symptoms); and the change in EDS (a VAS questionnaire for self-assessment of symptoms, range from 0 to 100, where 0 coded for “No symptoms”, 50 for “Moderate symptoms”, and 100 for “Unbearable symptoms”).

Exploratory endpoints included: (1) the change in percentage of area loss of meibomian glands, as evaluated with infra-red meibography performed with the Antares topographer and Phoenix software analysis (CSO) or the Keratograph 5M and Meiboscan software (Oculus). A 5-point scale (no gland loss, < 25% loss, 2 = 25%—50%, 51–75%, and > 75%), was used to score the severity of area loss of meibomian glands; (2) the change in meibomian gland score or MGS as described by Lane and colleagues [9] (the sum of grades of 15 meibomian glands (5 nasal + 5 central + 5 temporal) along the lower eyelid, where each gland was graded 0 if blocked, or 1, 2, 3 if the expressed meibum was inspissated, cloudy liquid, or clear liquid, respectively); and (3) change (Normal versus Abnormal) in eyelid appearance in biomicroscopy evaluation with the slit lamp, including lid margin thickening, conjunctival injection, and loss of eye lashes.

The change in the severity of rosacea, from baseline to follow-up, was a post-hoc outcome measure. Skin rosacea was evaluated in the malar region. Severity was graded using the standard classification method proposed by the National Rosacea Society Expert Committee (2004). Additional post-hoc analyses were conducted for parameters collected after each treatment session, including the number of expressible glands along the upper and lower eyelids; the predominant quality of meibomian gland secretions along the upper and lower eyelids; the daily use of artificial tear drops and warm compresses (as reported by the participants); and the level of pain/discomfort due to IPL and MGX (each, self-assessed with a VAS ranging from 0 to 100, where 0 implied no pain and 100 implied most severe and intolerable pain).

### Change to outcomes

In the original design of the study, outcome measures were to be collected in both eyes but the primary study hypothesis was to be evaluated for the study eye only. Following the finding that 8 patients in one site were not treated in accordance with the treatment protocol (see “Changes to trial design”), the study statistician recommended to include both eyes in the statistical analysis. Correspondingly, the protocol was amended and approved by the IRB.

### Sample size

To detect a statistically significant difference in the change of TBUT between the study and control arms, it was assumed that the changes in TBUT are expected to be 5±5 sec and 1±5 sec in the study arm and control arm, respectively (based on preliminary results obtained in a pilot study [24]), and on the literature on the effect of meibomian gland expression). With a two-sided 5% significance and a power of 80%, the study statistician determined that a minimal sample size of 50 evaluable subjects (100 evaluable eyes) would be required. Following the finding that several patients in one site were not treated in accordance with the treatment protocol (see Changes to trial design), the study statistician recommended to increase the sample size to 166 evaluable eyes (83 evaluable subjects).

### Interim analyses and stopping guidelines

There were no interim analyses, and no stopping rules were defined.

### Randomization: Type

The randomization process adopted a blocked randomization strategy, using random block size of 2 and 4. Prior to study commencement a randomization sequence was created, per site, by the study statistician.

### Randomization: Allocation concealment mechanism

A clinical research associate (Sierra Clinical) provided each site with a set of sealed and opaque envelopes, each containing a randomized assignment as prepared by the study statistician. Each envelope was labeled with a unique and consecutive number.

### Randomization: Implementation

Following enrollment of a participant, the site study coordinator opened the envelope and determined the allocation. This information was conveyed to the treating physician only. The site examiners remained blinded.

### Blinding

Allocation was not disclosed to the patients. Since the eyes were occluded and the patient did not know what level of discomfort to expect, the patient was effectively blinded to the allocation. Allocation was also concealed from the examiners who assessed the outcome measures at baseline, the follow-up, and at each treatment session. Due to the nature of treatment, it was impossible to mask the individuals who delivered the IPL treatment itself. However, the individuals who performed MGX were also blinded to the allocation.

### Similarity of interventions

Except for the blocking of IPL energy in the control arm, versus active IPL in the study arm, interventions in the two arms were identical.

### Statistical methods

Statistical planning and analysis of the primary, secondary and exploratory endpoints was done by the study statistician, using the R software. Sub-analyses and analyses of post-hoc outcomes were performed by the sponsor with JMP 16.0.0.0 (SAS Institute, Inc.). Imputation of missing data was carried out with excel simulations.

OSDI, rosacea severity, use of warm compresses, and use of artificial tears were collected per subject. All other outcome measures were collected per eye.

For continuous variables, descriptive statistics were expressed as median, mean (μ) ± standard deviation (σ), μ ± standard error of the mean (SEM), or 95% confidence intervals (95% CI: low, high). Within study arm, the change (ΔX_*arm*_) of a continuous outcome measure X was calculated as X(FU)-X(BL), averaged across all subjects in that specific arm, where X(FU) and X(BL) are the values of the outcome measure at the follow up and the baseline, respectively. The statistical significance of ΔX was estimated with a paired two-tails t-test and the resulting p-value was noted with a small p. Between study arms, the statistical significance of the mean difference (MD_X =_ ΔX_*study*_—ΔX_*control*_) was estimated with a least squares fit model, where the change ΔX was the dependent variable, and the allocation (study arm versus control arm) was the independent variable. Results of a test evaluating the statistical significance of the difference between arms were represented with a large P. In outcome measures for which a statistically significant difference between the two arms was identified, an additional analysis was performed utilizing a more conservative approach. First, the inter-eye correlation was removed by using a linear mixed effect (LME) model with random intercept, with the change ΔX as the dependent variable, the allocation as an independent variable, and the subject identity as an independent variable with a random effect. Running this LME model is equivalent to defining the “average eye” (the arithmetic average between the value of the outcome measured in the left and right eye of a subject) as the dependent variable, and the application as the sole independent variable. Second, missing values were handled by implementing a multiple imputation technique: for each patient with a missing value at the follow-up, the missing value was imputed with the average result of 1000 simulations, where in each simulation the missing value of an outcome measure was replaced with *N*^−^(*p*_*i*_, μ_FU_, σ_FU_), where *N*^−^ is the inverse of a normal cumulative distribution with a mean μ_FU_ and a standard deviation μ_FU_ (the mean and standard deviation of the outcome measure at the follow-up, for the entire sample with available values at follow-up), and *p*_*i*_ is a random probability taken from a uniform distribution between 0 and 1. For simplicity, when applicable, results of this conservative analysis are mentioned in the text, but not in the Tables.

For ordinal categorical variables, descriptive statistics were expressed as frequencies and percentages; within arms, the statistical significance of the change from baseline to follow-up was examined with a Pearson’s chi-square test, or with a Fisher’s exact test (the latter, when > 20% of the cells in the contingency table had expected frequencies of less than 5); between arms, the statistical significance of the difference in number of eyes which improved/remained the same/deteriorated was estimated with an ordinal logistic regression.

## Results

### Participant flow

**Fig 1:**
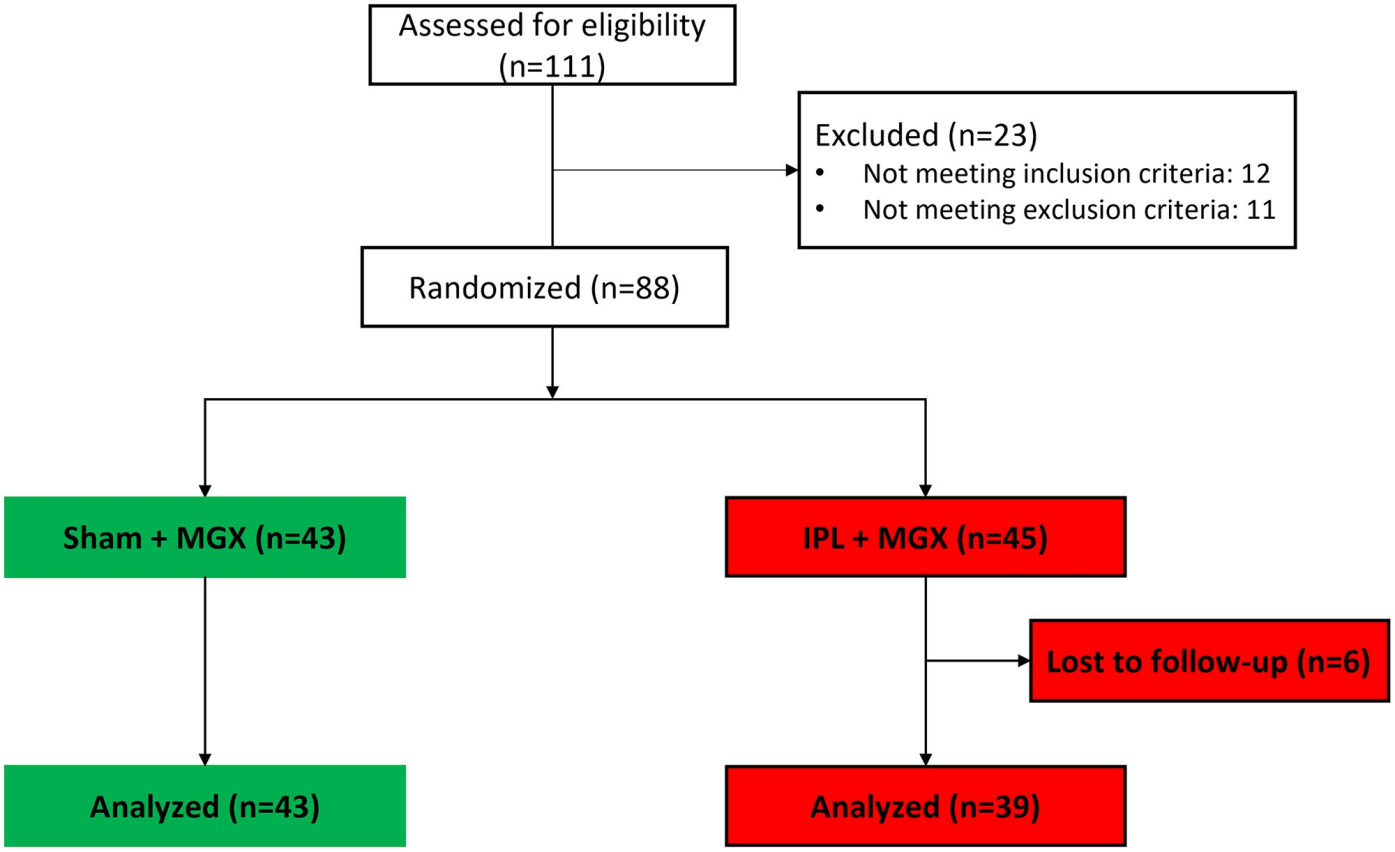
Flow diagram. n: number of subjects.

Fig 1 shows the flow diagram according to the CONSORT guidelines. One hundred eleven (111) subjects were screened between January 2018 and May 2019. Twelve subjects (12) were not eligible due to failure to meet all inclusion criteria (MGS > 12: n = 5; OSDI < 23: n = 4; TBUT > 7 seconds n = 3); Eleven (11) additional subjects were excluded due to use of photosensitive medications within 3 months prior to screening (n = 4), history or migraines, seizures or epilepsy (n = 2); ocular surface abnormality compromising corneal integrity (n = 2), use of prescription eyes drops for dry eye within 7 days prior to screening (n = 1), facial IPL within 12 months prior to screening (n = 1), and a combination of *Herpes simplex*, current use of punctal plugs, and precancerous lesions in the area of treatment (n = 1). Of the 88 eligible subjects, 43 and 45 were randomized to the sham treatment plus MGX (the control arm) and IPL plus MGX (the study arm), respectively. These 88 subjects constituted the safety set.

### Losses and exclusions

Of the 88 randomized subjects, 6 subjects (all assigned to the study arm) did not complete the full schedule of treatment: 3 withdrew after a single treatment session, 1 after two sessions, 1 after three sessions, and 1 after four sessions. Reasons for discontinuing the study were: subject did not want to continue due to pain of procedure (n = 1); subject did not want to abstain from anti-histamines (n = 1); and subject did not want to continue and gave no reason (n = 1). Three subjects were lost to follow-up without the possibility to inquire about their reasons to withdraw. None of the randomized subjects were excluded after randomization, The 82 subjects who completed all treatments sessions and the follow-up (43 and 39 subjects in the control and study arm, respectively) constituted the efficacy set.

### Recruitment

Patients were recruited between Jan 2018 and May 2019. Study duration per each patient was 10 weeks (4 treatment sessions at 2 weeks intervals + a follow-up at 4 weeks after the 4^th^ treatment session).

### Reason for stopped trial

Study ended when 82 subjects completed the study.

### Baseline data

Baseline data in the efficacy set are summarized in Table 1. Between the two arms, there were no differences in demographics (age: P = 0.3396; ethnicity: P = 0.7781; skin type: P = 0.9056). There was a trend form more women in the control arm (60% of women, versus 37% of men), but the difference was not statistically significant (P = 0.0503). The primary and secondary outcome measures were similar between the two arms (TBUT: P = 0.5253; OSDI: P = 0.1380; EDS: P = 0.1437). In the exploratory outcome measures, MGS and Meibography were more severe in the control arm, compared to the study arm (MGS: P = 0.0478; Meibography in lower lids: P = 0.0092; Meibography in upper lids: P = 0.0447).

**Table 1:** Baseline values.

		Level	Control (43 pts, 86 eyes)	Study (39 pts, 78 eyes)	P
Demographics					
Age (years) (Continuous)		Patient	56.8 [52.9, 60.7]	54.3 [49.8, 58.7]	0.3793
Gender (Nominal)		Patient	Women: 33 (76.7%)	Women: 22 (56.4%)	0.0503
			Men: 10 (23.3%)	Men: 17 (43.6%)	
Fitzpatrick skin type (Ordinal)		Patient	I: 4 (9.3%)	I: 5 (12.8%)	0.6340
			II: 19 (44.2%)	II: 18 (46.2%)	
			III: 15 (34.9%)	III: 11 (28.2%)	
			IV: 5 (11.6%)	IV: 5 (12.8%)	
Ethnicity (Nominal)		Patient	Caucasian: 37 (86.1%)	Caucasian: 32 (82.1%)	0.7781
			Hispanic: 5 (11.6%)	Hispanic: 5 (12.8%)	
			Asian/Pacific: 1 (2.3%)	Asian/Pacific: 2 (5.1%)	
Primary outcome					
TBUT (sec) (Continuous)		Eye	3.8 [3.4, 4.1]	4.0 [3.6, 4.4]	0.5253
Secondary outcomes					
OSDI (Continuous)		Patient	60.2 [54.6, 65.9]	53.8 [47.1, 60.5]	0.1380
EDS (Continuous)		Eye	71.0 [67.7, 74.3]	67.0 [62.6, 71.4]	0.1437
Exploratory outcomes					
MGS		Eye	8.4 [7.6, 9.2]	9.6 [8.9, 10.4]	0.0478 *
Meibography (%Loss of meibomian glands)	Lower lids (Ordinal)	Eye	None: 10 (11.6%)	None: 13 (16.7%)	0.0092 **
			< 25%: 47 (54.7%)	< 25%: 52 (66.6%)	
			25–50%: 16 (18.6%)	25–50%: 13 (16.7%)	
			51–75%: 9 (10.5%)	51–75%: 0 (0%)	
			>75%: 4 (4.6%)	>75%: 0 (0%)	
	Upper lids (Ordinal)	Eye	None: 14 (16.3%)	None: 15 (19.2%)	0.0447 *
			< 25%: 42 (48.8%)	< 25%: 47 (60.3%)	
			25–50%: 18 (20.9%)	25–50%: 15 (19.2%)	
			51–75%: 8 (9.3%)	51–75%: 1 (1.3%)	
			>75%: 4 (4.7%)	>75%: 0 (0%)	
Post-hoc outcomes					
Number of Expressible glands	Lower lids (Continuous)	Eye	10.6 [9.3, 11.9]	11.5 [10.2, 12.8]	0.3201
	Upper lids (Continuous)	Eye	10.7 [8.9, 12.5]	10.3 [8,7, 12.0]	0.7753
Predominant quality of meibomian gland secretion	Lower lids (Ordinal)	Eye	0 (Blocked): 10 (11.6%)	0 (Blocked): 5 (6.4%)	0.1389
			1 (Inspissated): 49 (57.0%)	1 (Inspissated): 41 (52.6%)	
			2 (Cloudy): 24 (27.9%)	2 (Cloudy): 32 (41.0%)	
			3 (Clear): 3 (3.5%)	3 (Clear): 0 (0%)	
	Upper lids (Ordinal)	Eye	0 (Blocked): 17 (19.8%)	0 (Blocked): 10 (12.8%)	0.1384
			1 (Inspissated): 39 (45.3%)	1 (Inspissated): 33 (42.3%)	
			2 (Cloudy): 28 (32.6%)	2 (Cloudy): 28 (35.9%)	
			3 (Clear): 2 (2.3%)	3 (Clear): 7 (9.0%)	
Skin Rosacea (Ordinal)		Patient	0 (None): 3 (7.0%)	0 (None): 6 (15.4%)	0.1418
			1 (Mild): 21 (48.8%)	1 (Mild): 22 (56.4%)	
			2 (Moderate): 16 (37.2%)	2 (Moderate): 7 (17.9%)	
			3 (Severe): 3 (7%)	3 (Severe): 4 (10.3%)	
Artificial tears (daily use) (Continuous)		Patient	2.7 [1.9, 3.6]	2.5 [1.8, 3.2]	0.6484
Warm compresses (daily use) (Continuous)		Patient	0.6 [0.2, 1.0]	0.41 [0.2, 0.6]	0.4625
Lid Margin thickening in biomicroscopy (Nominal)		Eye	Abnormal: 69 (80.2%)	Abnormal: 55 (70.5%)	0.1477
			Normal: 17 (19.8%)	Normal: 23 (29.5%)	
Conjunctival injection in biomicroscopy (Nominal)		Eye	Abnormal: 63 (73.3%)	Abnormal: 55 (70.5%)	0.6962
			Normal: 23 (26.7%)	Normal: 23 (29.5%)	
Loss of eye lashes in biomicroscopy (Biomicroscopy) (Nominal)		Eye	Abnormal: 26 (30.2%)	Abnormal: 19 (24.4%)	0.3999
			Normal: 60 (69.8%)	Normal: 59 (75.6%)	

Continuous variables: Mean and 95% confidence interval (μ [Low 95%, High 95%]). Categorical variables: frequency and percentage per category (n (%)); Statistical significance (P) was calculated with a two-sided t-test for continuous variables, ordinal logistic regression for ordinal variables, or Pearson’s chi-square test for nominal variables;

*: P<0.05;

**:P<0.01;

With respect to the post-hoc outcome measures, at baseline there were no differences between the two arms in the number of expressible glands in the lower lids (P = 0.3201), the number of expressible glands in the upper lids (P = 0.7753), the predominant quality of meibomian gland secretion in the lower lids (P = 0.1389), the predominant quality of meibomian gland secretion in the upper lids (P = 0.1384), the severity of skin rosacea (P = 0.1418), the number of daily artificial tears (P = 0.6484), the number of daily warm compresses (P = 0.4625), abnormal biomicroscopy findings in lid margin (P = 0.1477), abnormal biomicroscopy findings in the conjunctiva (P = 0.6962), and abnormal biomicroscopy findings in the eye lashes (P = 0.3999.

### Number analyzed

The primary analysis was intention-to-treat (ITT). Of the 88 randomized participants, 6 (all in the study group) were lost to follow-up. Thus, data from 82 patients were available for the ITT analysis. The final number of participants in the control and study groups were 43 patients (86 eyes) and 39 patients (78 eyes), respectively.

### Outcome measures tested at BL and FU

Tables 2 and 3 summarize the change in outcome measures tested at BL and FU.

**Table 2:** Change of continuous outcome measures tested at BL and FU. Adjusted: by value of the variable at baseline.

Outcome measure	Arm	FU		Change from BL (FU-B)		p (within arms)	P (Between arms)
		Mean [95%CI: low, high] (n)	Median	Mean [95%CI: low, high] (n)	Median		
**TBUT (sec)**	Control	4.5 [4.0, 5.1] (86)	3.85	0.7 [0.3, 1.2] (86)	0.4	0.0021** ↑	0.0147* Adjusted: 0.0076**
	Study	6.0 [5.4, 6.6] (78)	5.85	2.0 [1.4, 2.6] (78)	2	<.0001**** ↑	
**OSDI (0–100)**	Control	34.3 [27.5, 41.1] (43)	33.3	-25.9 [-33.5, -18.3] (43)	-27.1	<.0001**** ↑	0.9984 Adjusted: 0.3518
	Study	27.9 [21.5, 34.3] (39)	20.4	-25.9 [-33.1, -18.6] (39)	-25.5	<.0001**** ↑	
**EDS (0–100)**	Control	48.9 [43.5, 54.3] (86)	50	-22.1 [-28.0, -16.2] (86)	-15.5	<.0001**** ↑	0.0072** Adjusted: 0.0001***
	Study	34.0 [29.6, 38.5] (78)	32	-33.0[-38.1, -27.8] (78)	-31	<.0001**** ↑	
**MGS (0–45)**	Control	13.6 [12.1, 15.1] (86)	13	5.2 [3.8, 6.6] (86)	5	<.0001**** ↑	<.0001**** Adjusted by MGS at baseline: <0.0001****
	Study	28.2 [25.5, 30.9] (78)	26	18.5 [15.8, 21.2] (78)	16.5	<.0001**** ↑	
**Artificial tears (daily use)**	Control	2.1 [1.2, 3.0] (43)	1	-0.65 [-1.3, -0.05] (43)	0	0.0176* ↑	0.8216 Adjusted: 0.6248
	Study	1.7 [1.2, 2.3] (39)	2	-0.74 [-1.3, -0.19] (39)	0	0.0050** ↑	
**Warm compresses (daily use)**	Control	0.3 [0.1, 0.5] (43)	0	-0.3 [-0.7, 0.1] (43)	0	0.0566	0.3525 Adjusted: 0.5706
	Study	0.31 [0.1, 0.5] (39)	0	-0.1 [-0.3, 0.1] (39)	0	0.1267	

For all variables, the value at FU and the change from BL to FU are represented with Mean and the 95% confidence interval (μ [Low 95%, High 95%]), and the median. Within each arm, p tests the null hypothesis that there is no change between BL (Table 1) and FU (two-sided paired t-test). P tests the null hypothesis that the change is similar between the two arms (least squares fit). For eye level variables (TBUT, EDS and MGS), correlation between eyes was removed as explained in Methods.

*: P<0.05;

**: P<0.01;

***: P<0.001;

****: P<0.0001;

^↑^: Variable improved from BL to FU;

**Table 3:** Change of categorical outcome measures tested at BL and FU.

Outcome measure		Arm	FU Category n (%)	Change of Category from BL to FU: n (%)	p	P
**Biomicroscopy**	**Lid Margin thickening**	Control	Abnormal: 62 (72%) Normal: 24 (28%)	-1 (Deteriorated): 0 0 (Did not change): 79 (92%) +1 (Improved): 7 (8%)	0.2103	0.0605
		Study	Abnormal: 41 (52%) Normal: 37 (46%)	-1 (Deteriorated): 0 0 (Did not change): 64 (82%) +1 (Improved): 14 (18%)	0.0212*	
	**Conjunctival injection**	Control	Abnormal: 70 (81%) Normal: 16 (19%)	-1 (Deteriorated): 9 (11%) 0 (Did not change): 75 (87%) +1 (Improved): 2 (2%)	0.2024	0.0002***
		Study	Abnormal: 38 (49%) Normal: 40 (51%)	-1 (Deteriorated): 0 0 (Did not change): 61 (78%) +1 (Improved): 17 (22%)	0.0055**	
	**Loss of eyelashes**	Control	Abnormal: 25 (29%) Normal: 61 (71%)	-1 (Deteriorated): 5 (6%) 0 (Did not change): 75 (87%) +1 (Improved): 6 (7%)	0.8674	0.3594
		Study	Abnormal: 14 (18%) Normal: 64 (82%)	-1 (Deteriorated): 3 (4%) 0 (Did not change): 67 (86%) +1 (Improved): 8 (10%)	0.3267	
**Meibography (area loss)**	**Lower Lids**	Control	Normal: 10 (12%) < 25%: 45 (53%) 25–50%: 16 (19%) 51%-75%: 9 (11%) >75%: 4 (5%)	-1 (Deteriorated): 1 (1%) 0 (Did not change): 83 (99%) +1 (Improved): 0	1.000	0.1315
		Study	Normal: 15 (19.2%) < 25%: 51 (65.4%) 25–50%: 12 (15.4%) 51%-75%: 0 >75%: 0	-1 (Deteriorated): 1 (1%) 0 (Did not change): 73 (94%) +1 (Improved): 4 (5%)	0.9082	
	**Upper Lids**	Control	Normal: 14 (16%) < 25%: 38 (45%) 25–50%: 20 (24%) 51%-75%: 7 (8%) >75%: 5 (6%)	-1 (Deteriorated): 5 (6%) 0 (Did not change): 79 (94%) +1 (Improved): 0	0.9773	0.0699
		Study	Normal: 13 (16.7%) < 25%: 52 (66.6%) 25–50%: 13 (16.7%) 51–75%: 0 >75%: 0	-1 (Deteriorated): 3 (4%) 0 (Did not change): 70 (90%) +1 (Improved): 5 (6%)	0.5645	
**Rosacea**		Control	Normal: 4 (9%) Mild: 21 (49%) Moderate: 14 (33%) Severe: 4 (9%)	-1 (Deteriorated): 3 (6%) 0 (Did not change): 35 (81%) +1 (Improved): 5 (11%)	0.9397	0.0506
		Study	Normal: 9 (23.1%) Mild: 25 (64.1%) Moderate: 4 (10.2%) Severe: 1 (2.6%)	-1 (Deteriorated): 0 0 (Did not change): 30 (77%) +1 (Improved): 9 (23%)	0.3327	

For all variables, the frequency and percentage are represented per category at FU. Also represented are the number and percentage of patients/eyes which improved, did not change, or improved from BL. Within each arm, p tests the null hypothesis that there is no change in the number of patients across categories, between BL (Table 1) and FU (Chi-test). Between arms, P tests the null hypothesis that there is no difference in the number of patients who improved, remained the same, or deteriorated between the two arms (Ordinal logistic regression);

*: <0.05;

**: <0.01;

***: <0.001.

#### TBUT (primary outcome measure)

**Fig 2:**
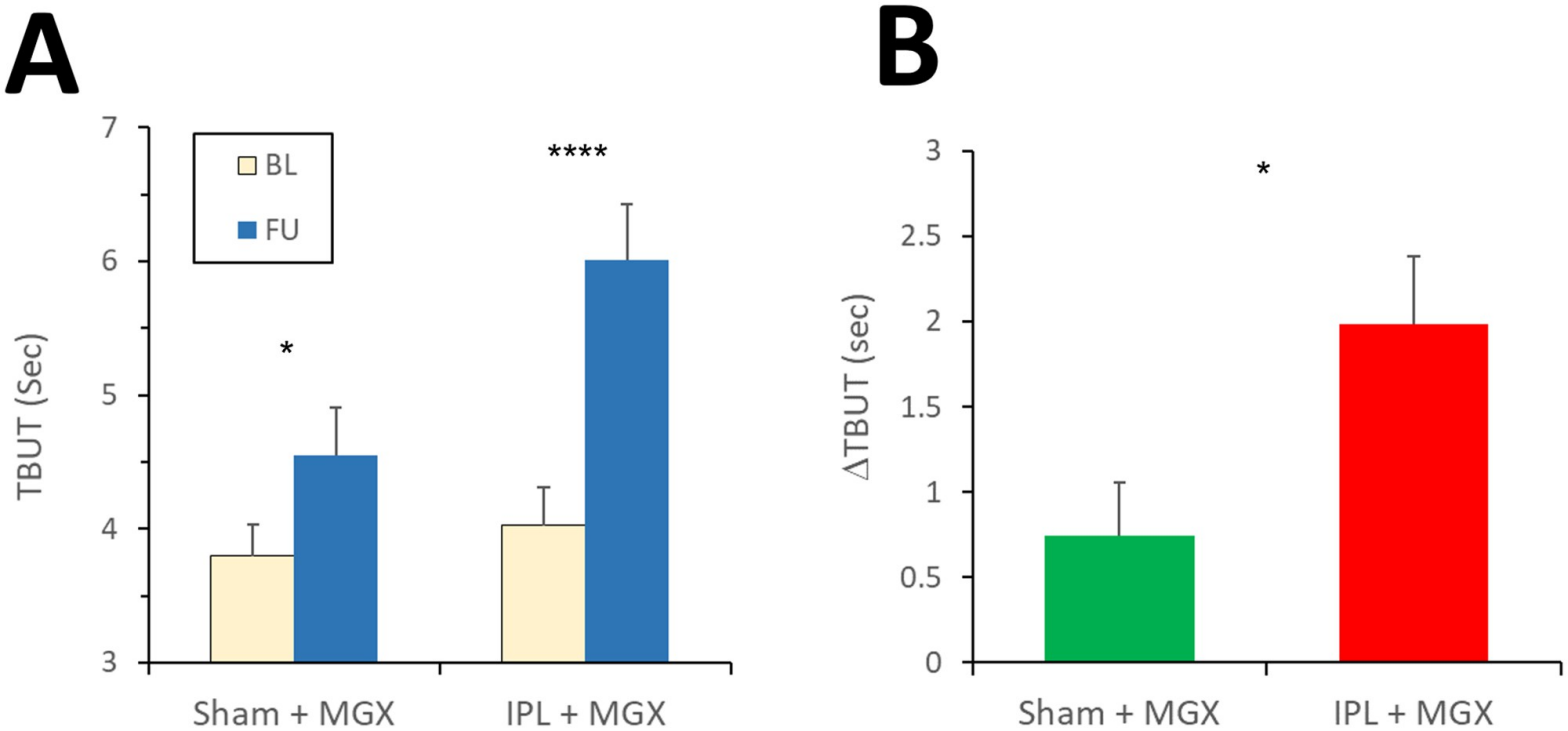
Change of primary outcome (TBUT). A. Absolute values of TBUT. Statistical tests within arms: paired two-sided t-test; **: p < 0.01; ****: p< 0.0001. B. ΔTBUT (the change of TBUT from BL to FU). Statistical test between arms: least squares fit of ΔTBUT. **: P<0.01.

TBUT improved (increased) in both arms, but the improvement was more pronounced in the study arm (compare Tables 1 & 2, and Fig 2). In the control arm, the mean TBUT increased from 3.8 [95% CI: 3.4, 4.1] to 4.6 [95% CI: 4.0, 5.1] seconds; the median TBUT increased from 3.7 to 3.85 seconds; and ΔTBUT (the difference of TBUT at FU and BL) was 0.75 [95% CI: 0.3–1.2] seconds (p < 0.01). In the study arm, the mean TBUT improved from 4.0 [95% CI: 3.6, 4.4] to 6.0 [95% CI: 5.4, 6.6] seconds, the median TBUT improved from 4.0 to 5.85 seconds, and ΔTBUT was 2.0 [95% CI: 1.4, 2.6] seconds (p < 0.0001). The mean difference between ΔTBUT of the two arms (MD_TBUT_ =) was 1.24 ±0.37 seconds (mean ± SEM), in favor of the study arm (P = 0.001). When the inter-eye correlation was taken into account, MD_TBUT_ was 1.24 ±0.50 seconds in favor of the study arm (unadjusted: p = 0.0147; adjusted by TBUT at baseline: p = 0.0076). Since this difference was statistically significant, a more conservative analysis (which handled missing values, as described in Methods), was also performed. In this conservative analysis, MD_TBUT_ slightly decreased to 1.20 ±0.48 seconds in favor of the study arm. This difference remained statistically significant, although to a lesser degree of certainty (P = 0.0136).

The minimal clinically important difference (MCID) for TBUT change was not previously characterized or defined in the literature. Hence, in a post-hoc analysis we examined several different possibilities for threshold values (2, 3, 4, and 5 seconds). We found that the proportions of eyes which exceeded these thresholds were twice or more in the study arm, compared to the control: 2 seconds- 90% vs. 44% (P = 0.00001); 3 seconds- 56% vs. 26% (P = 0.004); 4 seconds- 38% vs. 19% (P = 0.045); and 5 seconds- 26% vs. 9% (P = 0.049).

#### OSDI (secondary outcome measure)

**Fig 3:**
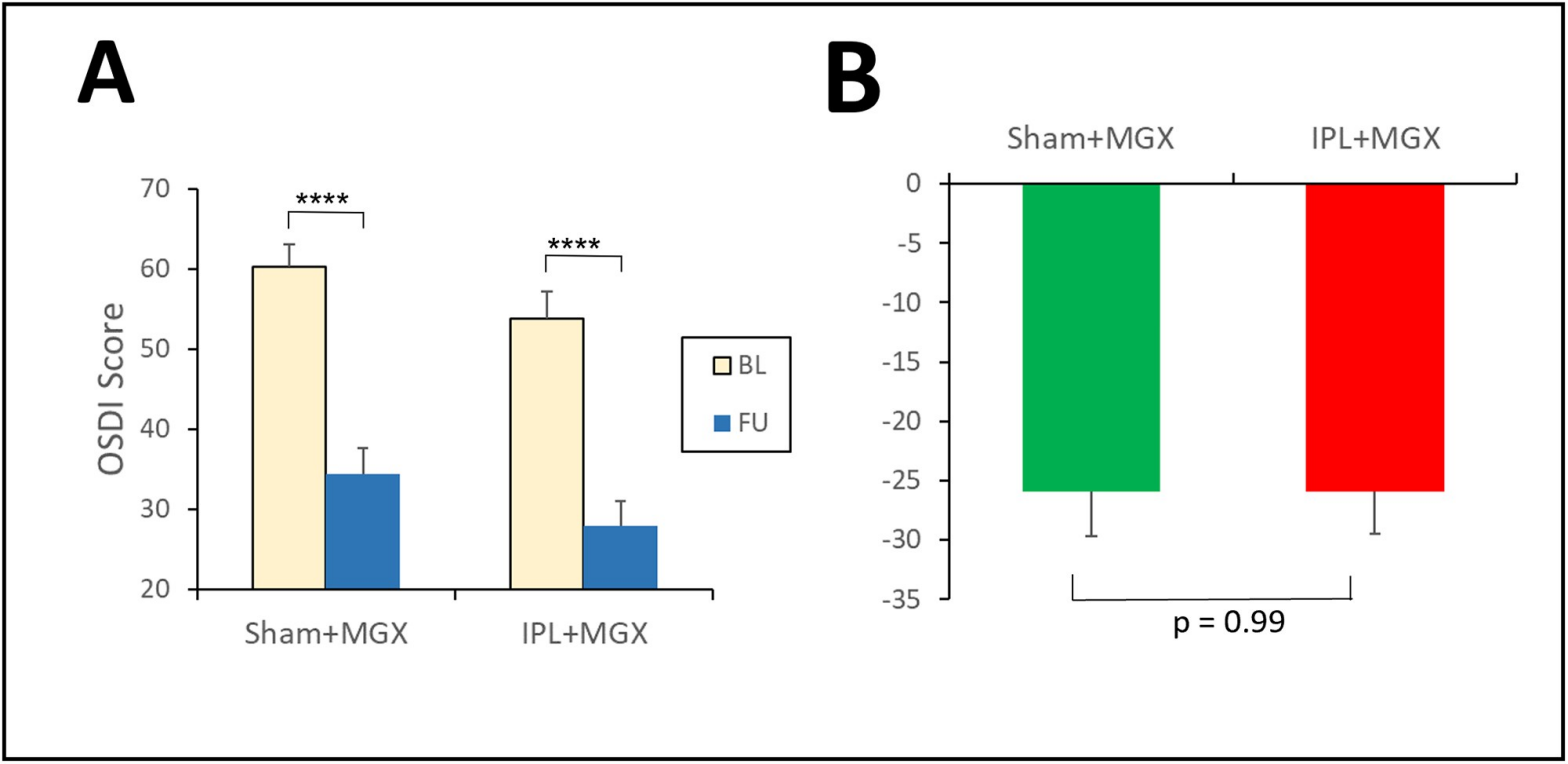
Change of symptoms (OSDI). **A**. Absolute values of OSDI; Statistical tests: 2-sided paired t-test of FU versus BL (within each arm); ****: p<0.0001. **B**. ΔTBUT; Statistical test: 2 sided least squares fit of ΔOSDI (between arms).

OSDI improved (decreased) in both arms (Compare Tables 1 & 2, and Fig 3). In the control arm, OSDI decreased from 60.2 ± 2.8 (μ±SEM) to 34.3 ± 3.4, with a ΔOSDI of -25.9 ± 3.6 points [95%CI: -33.5 to -18.3] (p<0.0001). In the study arm, OSDI decreased from 53.8 ± 3.3 to 27.9 ± 3.2, with a ΔOSDI of -25.9 ± 3.8 points [95%CI: -33.1 to -18.5] (p<0.0001). There were no significant differences between the two arms (unadjusted: P = 0.9984; adjusted by OSDI at baseline: P = 0.3518).

#### EDS (Secondary outcome measure))

EDS improved (decreased) in both arms (Compare Tables 1 & 2). In the control arm, ΔEDS decreased by -22.1±3.0 (mean±SEM) points [95%CI: -28 to -16.2] (p<0.0001). In the study arm, ΔEDS decreased by -33.0±2.6 points [95%CI: -38.1 to -27.8] (p<0.0001). The difference between ΔEDS of the two arms (MD_EDS_) was -10.8±4.0 (μ±SEM), in favor of the study arm (unadjusted: P = 0.0072; adjusted by EDS value at baseline: P = 0.0001). With a conservative analysis, this difference remained statistically significant (MD_EDS_ = -11.6 ± 5.2, mean ± SEM, in favor of the study arm; P = 0.0274).

#### MGS (Exploratory outcome measure)

**Fig 4:**
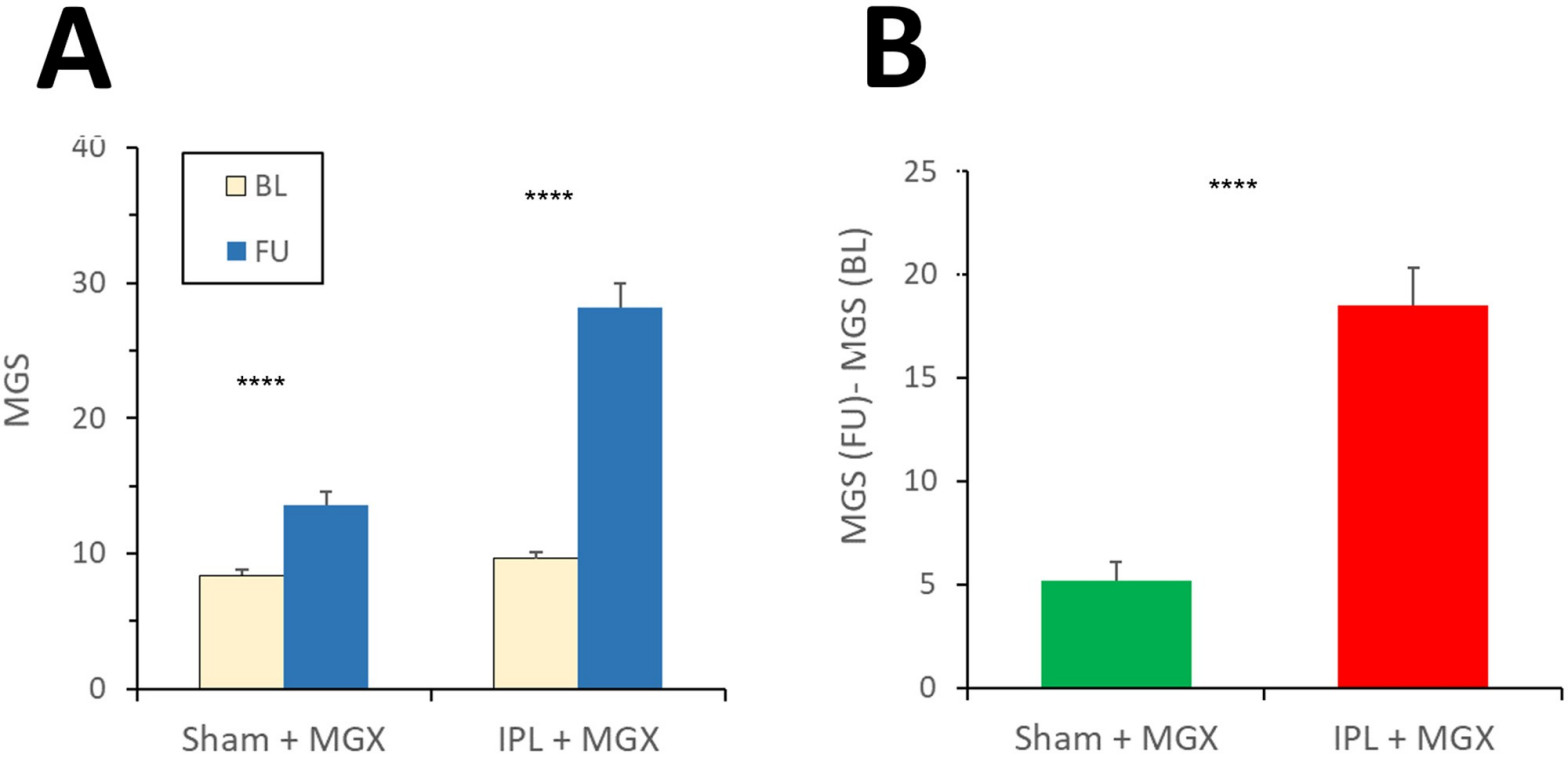
Change of MGS. **A**. Absolute values of MGS at BL and FU; ****: p (within arms) <0.0001. **B**. Change of MGS from BL to FU (ΔMGS); ****: P (between arms) <0.0001.

MGS improved (increased) in both arms, but the improvement was more pronounced in the study arm (Compare Tables 1 & 2, and Fig 4). ΔMGS increased by 5.2±0.7 (μ±SEM) points [95%CI: 3.8 to 6.6] and by 18.5±1.3 points [95%CI: 15.8 to 21.2] in the control and study arm, respectively. The difference between ΔMGS of the two arms (MD_MGS_) was 13.3 ± 1.5 (μ±SEM), in favor of the study arm (unadjusted: P<0.0001; adjusted by MGS value at baseline: P<0.0001). With a conservative analysis that hand, this difference remained statistically significant (MD_MGS_ = 12.8 ± 1.9, mean ± SEM, in favor of the study arm; P < 0.0001).

#### Meibography (Exploratory outcome measure)

Area loss of meibomian glands, as assessed with Meibography, was an exploratory outcome measure categorized in 5 levels, in each eyelid separately (Normal, less than 25%, between 25 and 50%, between 51% and 75%, and more than 75%). An improvement is defined as a switch from one level to a lower level. Within each arm, most eyelids did not change (lower lids: p = 1.000 for the control arm, and p = 0.9082 for the study arm; upper lids: p = 0.9773 for the control arm, and p = 0.5645 for the study arm). Between the two arms, in the lower lids there were no differences in the proportion of eyes which improved, deteriorated, or didn’t change (P = 0.1315); in the upper lids, there was a small tendency for more improvement in the study arm, but the difference was not statistically significant (P = 0.07).

#### Skin rosacea (Post-hoc outcome measure)

The severity of skin rosacea was defined as normal, mild, moderate, or severe. Within arms, there were no significant changes in the severity of skin rosacea (Control arm: p = 0.9397; Study arm: p = 0.3327). Between the two arms there was a tendency for more improvement in study patients compared to control patients, but the difference was not statistically significant (P = 0.0506).

#### Daily use of artificial tears (Post-hoc outcome measure)

In both arms, there was a statistically significant decrease the number of artificial tear drops used per day (Control arm: p = 0.0176; Study arm: p = 0.005). The difference between the two arms was not statistically significant (P = 0.8216).

#### Daily use of use of warm compresses (Post-hoc outcome measure)

Neither arm showed a significant decrease in the number of warm compresses used (Control arm: p = 0.0566; Study arm: p = 0.1267). There was no difference between the two arms (P = 0.3525).

#### Biomicroscopy (Post-hoc outcome measures)

For each of the examined features (lid margin thickening, conjunctival injection, and loss of eye lashes), an improvement is defined as an increase in the number of eyes which switched from abnormal to normal (Compare Tables 1 & 3).

*Lid margin thickening*. In the control arm, the percentage of normal eyelids increased from 19.8% to 28% (p = 0.2103). In the study arm, the percentage of eyes with normal lid margin increased from 20% to 46% (p = 0.0212). Between the two arms, there were no differences in the number of eyes which improved, deteriorated, of remained the same: more eyes improved in the study arm when compared to the control arm, but the difference was not statistically significant (18% vs 8%, P = 0.065).

*Conjunctival injection*. In the control arm, the percentage of normal eyelids *decreased* from 26.7% to 19% (p = 0.2024). In the study arm, the percentage of eyes with normal conjunctiva increased from 29.5% to 51% (p = 0.0055). More eyes improved in the study arm when compared to the control arm (22% vs 2%, P = 0.0002).

*Loss of eyelashes*. In the control arm, the percentage of normal eyelids remained stable (69.8% at BL, compared to 71% at FU; p = 0.8674). In the study arm, the percentage of eyes with normal eye lashes slightly increased from 75.6% to 82%, but the change was not significant (p = 0.3267). No differences were observed between the two arms (P = 0.3594).

### Outcome measures tested after each treatment session

Tables 4 and 5 summarize results of outcome measures tested after every treatment.

**Table 4:** Continuous outcome measures tested after every treatment session.

		Arm	Time = 0 (Tx1)	Time = 2 weeks (Tx2)	Time = 4 weeks (Tx3)	Time = 6 weeks (Tx4)	P
			Mean [95%CI: low, high] (n)	Mean [95%CI: low, high] (n)	Mean [95%CI: low, high] (n)	Mean [95%CI: low, high] (n)	
#Expressible glands	LL	Control	10.6 [9.3, 11.9] (86)	11.4 [104, 12.6] (86)	12.3 [10.9, 13.6] (86)	12.3 [11.0, 13.5] (86)	<0.0001****
		Study	1.5 [10.2, 12.8] (90)	15.7 [14.0, 17.4] (84)	18.1 [16.3, 19.9] (82)	20.0 [18.2, 21.5] (80)	
	UL	Control	10.7 [8,9, 12.5] (86)	9.8 [8.3, 11.3] (86)	10.5 [9.0, 12.0] (86)	11.2 [9.6, 12.8] (86)	<0.0001****
		Study	10.3 [8.7, 12.0] (90)	13.0 [11.0, 15.0] (84)	15.4 [13.2, 17.5] (82)	15.7 [13.6, 17.9] (82)	
Pain due to MGX		Control	43.0 [37.8, 48.2] (86)	40.5 [34.9, 46.1] (86)	39.9 [34.0, 45.9] (86)	36.7 [30.9, 42.4] (86)	0.0438*
		Study	48.8 [43.4, 54.1] (90)	43.4 [37.7, 43.4] (84)	39.0 [33.5, 44.5] (82)	34.7 [29.2, 40.2] (80)	
Pain due to IPL		Control	2.9 [1.6, 4.2] (86)	4.4 [2.0, 6.8] (86)	3.8 [2.6, 5.2] (86)	4.2 [2.6, 5.8] (86)	<0.0001****
		Study	50.0 [44.1, 55.6] (90)	45.4 [40.6, 50.2] (84)	42.2 [36.9, 47.6] (82)	40.7 [35.2, 46.1] (80)	

LL = Lower lids; UL = Upper lids; For all variables, the values at Tx1, Tx2, Tx3 and Tx4 are represented with Mean and 95% confidence interval (μ [Low 95%, High 95%]). P (least squares fit) tests the null hypothesis that the change from Tx1 to Tx4 is similar between the two arms.

*: P<0.05;

****: P<0.0001;

**Table 5:** Categorical outcome measures tested after every treatment session.

		Arm	Time = 0 (Tx1)	Time = 2 weeks (Tx2)	Time = 4 weeks (Tx3)	Time = 6 weeks (Tx4)	Change	P
			N (%)	N (%)	N (%)	N (%)		
Meibum Quality (0 = Blocked to 3 = clear liquid)	LL	Control	Blocked: 10 (12%) Inspissated: 49 (57%) Cloudy: 24 (28%) Clear: 3 (3%)	Blocked: 3 (3.5%) Inspissated: 45 (52.3%) Cloudy: 38 (44.2%) Clear: 0 (0%)	Blocked: 4 (5%) Inspissated: 54 (63%) Cloudy: 28 (32%) Clear: 0 (0%)	Blocked: 3 (3.5%) Inspissated: 52 (60.4%) Cloudy: 31 (36.1%) Clear: 0 (0%)	-1: 17 (20%) 0: 42 (49%) +1: 27 (31%)	P<0.0001****
		Study	Blocked: 6 (7%) Inspissated: 46 (51%) Cloudy: 38 (42%) Clear: 0 (0%)	Blocked: 0 (0%) Inspissated: 21 (25%) Cloudy: 52 (62%) Clear: 11 (13%)	Blocked: 1 (1%) Inspissated: 13 (16%) Cloudy: 37 (45%) Clear: 31 (38%)	Blocked: 0 (0%) Inspissated: 10 (12.5%) Cloudy: 38 (47.5%) Clear: 32 (40%)	-1: 1 (1%) 0: 31 (39%) +1: 48 (60%)	
	UL	Control	Blocked: 17 (20%) Inspissated: 39 (45%) Cloudy: 28 (33%) Clear: 2 (2%)	Blocked: 11 (13%) Inspissated: 38 (44%) Cloudy: 36 (42%) Clear: 1 (1%)	Blocked: 12 14%) Inspissated: 32 (37%) Cloudy: 41 (48%) Clear: 1 (1%)	Blocked: 7 (8%) Inspissated: 36 (42%) Cloudy: 39 (45%) Clear: 4 (5%)	-1: 9 (10%) 0: 30 (35%) +1: 47 (55%)	P = 0.0009***
		Study	Blocked: 14 (17%) Inspissated: 34 (40%) Cloudy: 29 (35%) Clear: 7 (8%)	Blocked: 10 (12%) Inspissated: 20 (24%) Cloudy: 25 (30%) Clear: 28 (34%)	Blocked: 6 (7.3%) Inspissated: 9 (11%) Cloudy: 29 (35.4%) Clear: 38 (46.3%)	Blocked: 4 (5%) Inspissated: 13 (16%) Cloudy: 22 (28%) Clear: 41 (51%)	-1: 9 (11%) 0: 19 (24%) +1: 52 (65%)	

LL = Lower lids, UL = Upper lids; -1 = Deteriorated, 0 = No change, +1 = Improved; The frequency and percentage of each meibum quality level are represented at Tx1, Tx2, Tx3 and Tx4. P tests the null hypothesis that the percentage of eyelids which improved, remained the same or deteriorated are similar between the two arms (ordinal logistic regression).

**: P<0.01;

****: P<0.0001;

#### Number of expressible glands (Post-hoc outcome measure)

**Fig 5:**
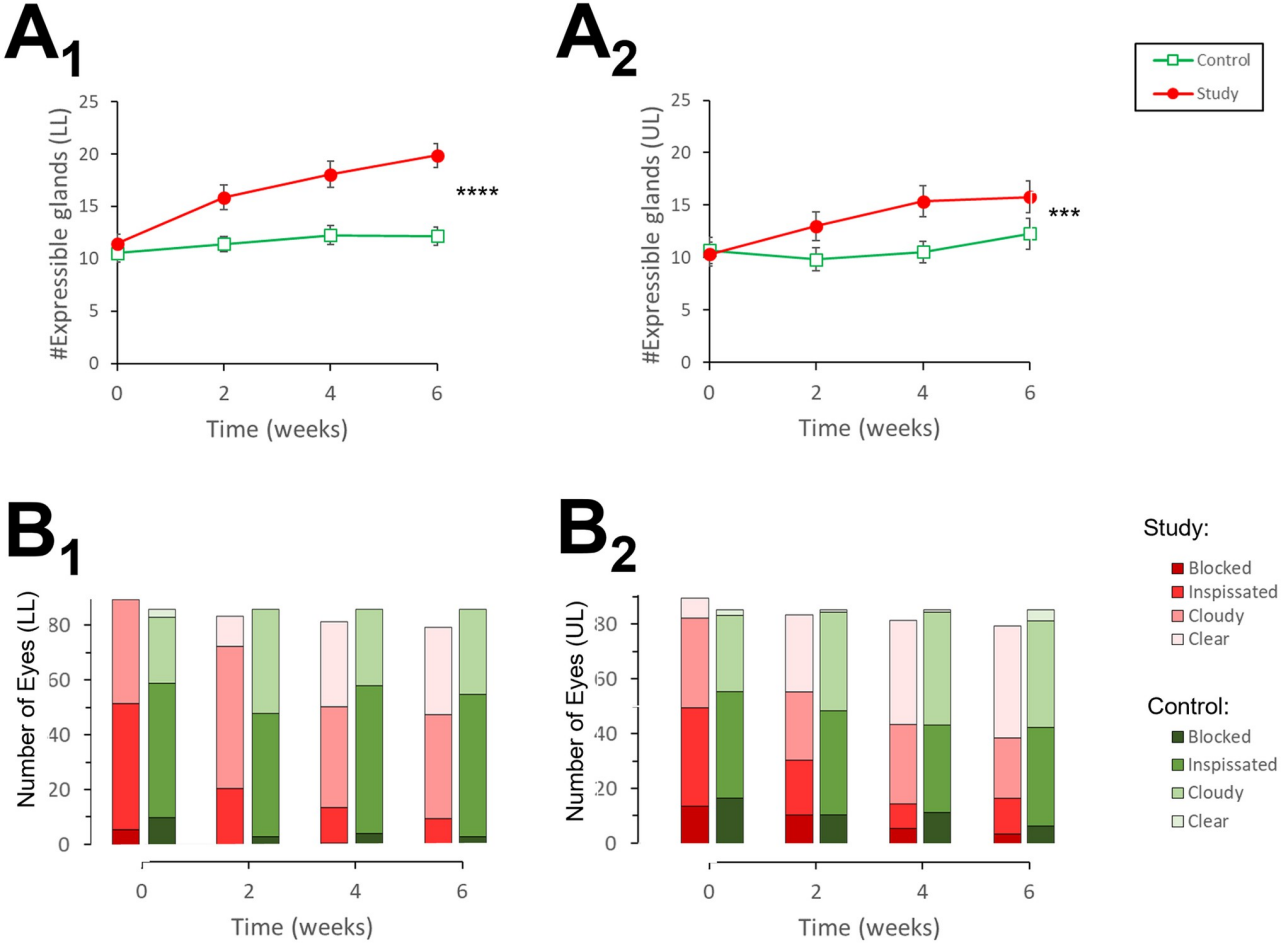
Number of expressible glands and meibum quality as function of time. Time: 0 = Tx 1, 2 weeks = Tx 2, 4 weeks = Tx 3, 6 weeks = Tx 4; A. Number of expressible gland in lower lids (A1) and upper lids (A2). Error bars: SEM; ***: P (between arms) < 0.001; ****: P (between arms) <0.0001. B. Distribution of predominant quality of meibum (blocked, inspissated, cloudy liquid, clear liquid) in study and control eyes. B1: lower lids. B2: upper lids.

The number of expressible glands, as function of time, is illustrated in Fig 5A. In the lower lids, in both arms the number of expressible glands increased: From Tx1 to Tx4, there was a change of 1.7 glands [95% CI: 0.59 to 2.86] in the control arm, versus a change of 8.0 glands [95% CI: 6.6 to 9.4]) in the study arm (Fig 5A_1_). At Tx4, the number of expressible glands in the lower lid was 19.9 [95% CI: 18.2, 21.5]) in the study arm, compared to 12.3 [95% CI: 11.0, 13.6] in the control arm (P<0.0001). Recall that at the baseline (Tx1) there was no statistically significant difference between the two arms (P = 0.3201, Table 1).

In the upper lids, there number of expressible glands in the control arm remained stable, while it increased in the study arm (Fig 5A2). From Tx1 to Tx4, there was an increase of 0.53 glands [95% CI: -0.78 to 1.85] in the control arm, compared an increase of 5.1 glands [95% CI: 3.52 to 6.73] in the study arm. At Tx4, the number of expressible glands in the upper lid was 15.7 [95% CI: 13.6, 17.9] in the study arm, compared to 11.2 [95% CI: 9.6, 12.8] in the control arm (P < 0.001). At Tx1, however, there was no difference between the arms (P = 0.7753, Table 1).

#### Pain due to MGX (post-hoc outcome measure)

In the control arm, pain decreased from 43 [95% CI: 37.8, 48.2] at Tx1 to 36.7 [95% CI 30.9, 42.4] at Tx4. In the study arm, pain decreased from 48.8 [95% CI: 43.3, 54.2] at Tx1 to 34.8 [95%CI: 29.3, 40.2] at Tx4. At Tx4, the difference between the two arms was statistically significant (P < 0.0001).

#### Pain due to IPL (post-hoc outcome measure)

In the control arm, pain due to IPL remained low, with 2.9 [95%CI: 1.7, 4.2] at Tx1 and 4.2 [95%CI: 2.6, 5.8] at Tx4. In the study arm, pain due to IPL decreased from 49.8 [95%CI: 44.1, 55.6] at Tx1 to 40.7 [95%CI: 35.3, 46.1] at Tx4. The difference between the arms was statistically significant (P < 0.0001).

#### Predominant quality of meibum (post-hoc outcome measure)

The predominant quality of meibum, as function of time, is illustrated in Fig 5B.

For the lower lids (Fig 5B_1_), in the control arm there was no significant change in the predominant quality of the meibum. For example, in the control arm the proportions of eyelids with predominantly dysfunctional meibomian glands was 69% (12% blocked + 57% inspissated) at Tx1, and 63.9% (3.5% blocked + 60.4% inspissated) at Tx4. In contrast, in the study arm the predominant quality of the meibum improved, with 58% dysfunctional glands at Tx1 (7% blocked + 51% inspissated) decreasing to 12.5% (0% blocked + 12.5% inspissated) at Tx4. The percentage of eyelids which deteriorated, remained the same, or improved was 20%, 49% and 31% in the control arm, compared to 1%, 39%, and 60% in the study arm. This difference was statistically significant (P<0.0001).

Similar results were observed for the upper lids. In the control arm, the proportions of eyelids with predominantly dysfunctional meibomian glands was 65% (20% blocked + 45% inspissated) at Tx1, and 50% (8% blocked + 42% inspissated) at Tx4. In contrast, in the study arm the predominant quality of the meibum decreased from 57% at Tx1 (17% blocked + 40% inspissated) to 21% (5% blocked + 16% inspissated) at Tx4. The percentage of eyelids which deteriorated, remained the same, or improved was 10%, 35% and 55% in the control arm, compared to 11%, 24%, and 65% in the study arm. This difference was statistically significant (P = 0.0009).

### Adverse events

The safety population included 88 randomized subjects. There were no serious adverse events reported. The incidence of adverse events was 8.9% in the study arm (mild: n = 1; moderate: n = 3), and 20.9% in the control arm (mild: n = 5; moderate: n = 3; severe: n = 1). Although there was a tendency for more adverse events in the control arm, the difference between the two arms was not statistically significant (P = 0.06).

In the study arm, 1 subject experienced 2 ocular-related adverse events (moderate allergic conjunctivitis and moderate bacterial conjunctivitis). The treating physician determined that the seasonal allergic conjunctivitis (detected first) was not related to the procedure nor the device, but the bacterial allergic conjunctivitis (detected two weeks later) was possibly related to the procedure (i.e., the meibomian gland expression). As a result of this adverse event, this subject was discontinued from the study. Another subject experienced mild skin pain (possibly related to the procedure). A third subject experienced moderate blepharitis (unrelated to both procedure and device). No subjects experienced systemic adverse events.

In the control arm, one subject experienced a severe conjunctival telangiectasia (unrelated to both procedure and device); one subject experienced a mild chalazion (unrelated) skin-related adverse events (mild chalazion, mild stye), and 6 subjects experienced systemic adverse events (mild bronchitis, mild sinus infection, moderate sinus infection, mild hyperlipidemia, and 2 cases of seasonal allergy worsening).

Except for the mild pain and the moderate bacterial conjunctivitis which were both possibly related to the procedure, none of the other reported adverse events were related to either the procedure or the device.

## Discussion

### Interpretation of results

Results of this study show that, in comparison with monotherapy of meibomian gland expression, the combination of IPL and meibomian gland expression was more effective in reducing signs of DED due to MGD. With respect to the primary outcome measure, TBUT increased in both arms but on average the change was 1.2 seconds longer in study eyes, compared to control eyes. The between-arms difference was statistically significant. The minimal clinically important difference (MCID) for TBUT change was not previously characterized or defined in the literature. Hence, in a post-hoc analysis we examined several different possibilities. We found that for cutoff values of 2,3,4,or 5 seconds, the proportion of eyes which exceeded these thresholds was twice or more in the study arm, compared to the control arm. For example, 56% vs. 26% of study vs. control eyes exceeded a TBUT change of 3 seconds, and 26% vs. 9% of study vs control eyes exceeded a TBUT change of 5 seconds. If one accepts that such TBUT changes are clinically meaningful, this finding suggests that, at least for some of the patients, IPL could be beneficial. IPL was particularly effective in improving signs related to the functionality of meibomian glands, such as MGS, the number of expressible glands, and the predominant quality of meibum. With respect to symptoms, there was a difference between several methods of evaluating symptoms. Using the OSDI questionnaire and the subject’s report of daily use of artificial tears, both study and control subjects improved but the difference between the two arms was mostly negligible. These results are in agreement with other studies with similar designs [25, 29]. Using the EDS questionnaire, in contrast, there was more improvement in study compared to control subjects. One possibility to explain the difference between these two types of questionnaires is that OSDI asks about the symptoms during the last week, whereas EDS is more general and does not restrict the subject to relate to any specific time range. The difference between OSDI and EDS results could reflect, for example, some added value that IPL has in the first few days after a treatment session, but would then fade out.

Why, between the two arms, significant differences in signs were not translated to significant differences in symptoms evaluated with the OSDI questionnaire? This is a key question. First, it is important to reiterate that dry eye disease is characterized by a poor correlation between signs and symptoms, perhaps due to the heterogeneity of DED itself [30], or because of a lack of well-defined diagnostic criteria commonly in use [3]. Moreover, MGX, which was described 100 years ago [31], is well-known for eliciting symptomatic relief for DED patients, as was observed in the current study, and also reported by others [14, 15, 16, 23]. Hence, it should not be surprising to find that symptoms were reduced not only in the study group, but also in the control. However, the lack of difference in OSDI, between the two arms, requires additional research for better understanding. This could reflect a flooring effect specific to this type of questionnaire: in responders, MGX would be sufficient to transiently reduce the OSDI score to a minimal level and, in those patients, the addition of IPL combined with MGX would not result in further improvement in OSDI.

### Mechanisms of action

The mechanism of action of IPL, with respect to DED due to MGD, is not yet fully understood. One possibility is that IPL closes abnormal telangiectasia and blocks the inflammatory mediators they secrete [22]. As a result, a major source of inflammation of the peri-orbital area is removed. Support for this mechanism is the finding that IPL significantly reduces the levels of key cytokines in tear samples [32]. Another possibility is that IPL activates cells by photobiomodulation (PBM). In PBM, light (especially in the red and near infra-red range) is absorbed within cytochrome C oxidase of mitochondria, resulting in a boost in ATP production and modulation of intracellular calcium levels [33]. Previous studies have shown that PBM can up-regulate anti-oxidant defenses, reduce reactive oxygen species in oxidative stressed cells, reduce the levels of pro-inflammatory cytokines in activated inflammatory cells, and even change the phenotype of macrophages (from a form specialized in killing bacteria and pathogens, to a form involved in removal of protein debris and stimulation of healing) [34]. In addition, IPL may also attenuate melanogenic gene overexpression, and suppress UVB-induced cytokine expression [35]. All of these could contribute to reduce inflammation and trigger healing mechanisms at the ocular surface and meibomian gland levels. A third possibility is that IPL could induce heat-shock production, as was demonstrated in skin cells [36, 37]. It is also possible that IPL reduces the population of Demodex mites, another significant risk factor in DED due to MGD [38, 39]. Other researchers proposed that meibomian gland health depends on relative hypoxia [40]. According to these researchers, loss of hypoxic conditions leads to MGD, and the thrombotic effects of IPL are useful for closing excessive blood vessels, thus restoring the hypoxic conditions necessary for normal function of the meibomian glands. Finally, there is the possibility that IPL generates heat which softens abnormally inspissated secretions of dysfunctional meibomian glands. This last explanation is, however, controversial. Some researchers proposed that even brief pulses of IPL are sufficient to transfer heat to the eyelids, melt an abnormally inspissated meibum within the meibomian glands, and thus facilitate their expression [41]. Other researchers argued that IPL can induce only short term thermal effects, with minimal changes in skin surface temperature [26]. According to this line of reasoning, IPL pulses are too brief to induce sustained changes of the meibum.

Several of our results bring further support to some of these potential mechanisms. First, although IPL was applied below the lower eyelids, the upper eyelids also responded positively to IPL: about one third of the increase in the number of expressible eyelids was observed in the upper lids. This result suggests that some factor, or some factors, is or are propagating some distance away from the site of IPL application, whether it is circulation of beneficial molecules (anti-inflammatory agents, anti-oxidants, heat shock proteins, etc.) via the orbital vasculature, or heat transfer through skin and connective tissues. Another interesting result is that patients in the study arm reported less pain associated with MGX, compared to patients in the control arm. Indeed, MGX is a forceful procedure which can be uncomfortable. With some patients, the procedure is not well-tolerated, even under the influence of local anesthetics [23]. For such patients, thus, monotherapy MGX may not be a practical option. Arita and colleagues reported that 3 of their study subjects (7%) refused to be treated with MGX alone, because of such pain, whereas none of the subjects treated with IPL and MGX complained of unacceptable discomfort. To explain these results, Arita and colleagues adopted the temperature increase as the mechanism of action at work, suggesting that IPL warms the meibomian glands, thereby melting the meibum, decreasing the pressure required for expression and, thus, reducing the pain associated with MGX. As we mentioned earlier, this explanation is not accepted by all. An alternative explanation is that from treatment to treatment the quality of meibum improves due to the PBM processes mentioned earlier. This would be reflected in reduced pain associated with MGX not at the current IPL session, but at the *following* one.

### Limitations of the study

The current study has several limitations. First, despite our efforts to mask the allocation and to include only patients naïve to IPL, it was not possible to completely ensure participant blinding. Since IPL is normally felt as a sensation ranging from mild discomfort to moderate pain, some patients could have correctly guessed their group assignment, based on their preliminary expectations and their sensations during the IPL treatment. Second, since study patients were treated with both MGX and IPL, it is difficult to isolate the contribution of IPL. In the design of the control arm, who were treated with MGX alone, we implicitly assumed that the two components are compounded, and therefore simple subtraction of the changes in the two arms should have given us a good estimation of the effect size. However, it is possible that the two components combine in a more complex way than simple linear addition. Another limitation of the study was that the follow-up period was relatively short. Further studies are necessary to elaborate on the durability of IPL’s long-term effectiveness. Next, this study was not designed to determine the efficacy of IPL in groups with different severity levels of MGD. Although between-group differences in baseline values of individual outcome measures were neutralized with statistical methods, it is possible that the baseline severity of MGD is not well defined by any of these outcome measures alone, but depends on complex interactions involving several such outcome measures. In such a case, between-group differences in the baseline severity of MGD could have biased the results. Future studies are required to determine the efficacy of IPL as function of MGD severity, so that clinicians may be better informed who of their patients are more likely to benefit from this technology. Finally, findings from this study is based on a specific population, namely patients with mild to moderate MGD, predominantly Caucasian, age 22–85, and with Fiztpatrick skin types I-IV (predominantly II to III). Future studies are warranted to justify the group differences in the more general population.

## Conclusions

The current study suggests that IPL, when combined with MGX, may be useful to improve signs and symptoms of MGD in a North American population. With respect to at least some of the signs, patients treated with IPL and MGX could benefit more than patients treated with MGX alone. Based on the results of this study, in February 2021 the US FDA issued an approval for the Lumenis device, for the use of IPL in management of DED due to MGD.

## Supporting information

S1 Dataset Data presented in this paper. (XLSX)

S1 Checklist CONSORT 2010 checklist of information to include when reporting a randomised trial*. (DOC)

S1 File Clinical study protocol. (DOCX)
