# Supplementary material for: Intense pulsed light improves signs and symptoms of dry eye disease due to meibomian gland dysfunction: A randomized controlled study
Source: PLoS One. 2022 Jun 23;17(6):e0270268. doi: 10.1371/journal.pone.0270268 (PMC9223330; doi:10.1371/journal.pone.0270268)
Supplement: S1 File — (DOCX) [file pone.0270268.s003.docx]

Clinical Study Protocol

**Study Title:** Effectiveness of Intense Pulsed Light (IPL) for improving signs and symptoms in Dry Eye Disease (DED) due to Meibomian Gland Dysfunction (MGD)

Study Synopsis

| **Study Device** | M22 intense pulsed light (IPL) module is a class II device with an FDA clearance (K142860) for benign cutaneous vascular lesions, including erythema of rosacea and facial telangiectasia. | |
| --- | --- | --- |
| **Protocol number** | LUM-VBU-M22-IPL-17-01 | |
| Study Title | Effectiveness of Intense Pulsed Light (IPL) for improving signs and symptoms in Dry Eye Disease (DED) due to Meibomian Gland Dysfunction (MGD) | |
| **Study design** | Multi-center, prospective, randomized, sham-controlled, superiority, non-significant risk | |
| **Projected Study period** | Initiation Date: January 15, 2018 | Completion Date: July 1, 2018 |
| **Study population** | Up to 83 male or female subjects, aged 22-85 with signs and symptoms of DED caused by MGD Enrollment will continue until at least 10 subjects without skin rosacea are enrolled. | |
| **Investigational device** | Lumenis^®^ M22 system with the IPL handpiece | |
| **Study Duration** | From screening to termination, each subject will participate in the study for 10-11 weeks (depending on whether screening & enrollment occurred on the same day or up to 1 week later)  The total study duration from the screening of the first subject to termination of the last subject is estimated to be 75 weeks. | |
| **Main Inclusion Criteria** | - Tear break-up time (TBUT) ≤ 7 seconds - MGS ≤ 12 for 15 glands in the lower eyelid - At least 5 non-atrophied meibomian glands in the lower eyelid - Symptoms self-assessed using the OSDI questionnaire ≥ 23 | |
| **Detailed Inclusion Criteria:** | - Subject is able to read, understand and sign an IC form - 22-85 years of age - Fitzpatrick skin type I-IV - Subject is able and willing to comply with the treatment/FU schedule and requirements - In the study eye, TBUT ≤ 7 seconds (PMID: [21450918](https://www.ncbi.nlm.nih.gov/pubmed/21450918)) - In the study eye, MGS ≤ 12 (PMID: [2222996](https://www.ncbi.nlm.nih.gov/pubmed/22222996)) - In the study eye, at least 5 non-atrophied meibomian glands in the lower eyelid - Symptoms self-assessed using the OSDI questionnaire ≥ 23 (PMID: [20065224](https://www.ncbi.nlm.nih.gov/pubmed/20065224)) | |
| **Main Exclusion Criteria:** | - Fitzpatrick skin type V or VI - Contact lens wear within the month prior to screening - Unwilling to discontinue use of contact lenses for the duration of the study - Ocular surgery or eyelid surgery, within 6 months prior to screening - Neuro-paralysis in the planned treatment area, within 6 months prior to screening - Other uncontrolled eye disorders affecting the ocular surface, for example active allergies - Current use of punctal plugs - Pre-cancerous lesions, skin cancer or pigmented lesions in the planned treatment area - Uncontrolled infections or uncontrolled immunosuppressive diseases - Subjects with ocular infections, within 6 months prior to screening - Prior history of cold sores or rashes in the perioral area or in the planned treatment area that could be stimulated by light at a wavelength of 560 nm to 1200 nm, including: Herpes simplex 1 & 2, Systemic Lupus erythematosus, and porphyria - Within 3 months prior to screening, use of photosensitive medication and/or herbs that may cause sensitivity to 560-1200 nm light exposure, including: Isotretinoin, Tetracycline, Doxycycline, and St. John's Wort - Over exposure to sun, within 4 weeks prior to screening - Use of prescription eye drops for dry eye, within 7 days prior to screening, excluding artificial tears and glaucoma drops - Radiation therapy to the head or neck, within 12 months prior to screening - Planned radiation therapy, within 8 weeks after the last treatment session - Treatment with chemotherapeutic agent, within 8 weeks prior to screening - Planned chemotherapy, within 8 weeks after the last treatment session - New topical treatments within the area to be treated, or oral therapies, within 3 months prior to screening- except over-the-counter acetaminophen-based analgesics for pain management, new oral omega 3 fatty acid supplements and topical artificial tears - Change in dosage of any systemic medication, within 3 months prior to screening - Anticipated relocation or extensive travel outside of the local study area preventing compliance with follow-up over the study period - Legally blind in either eye - History of migraines, seizures or epilepsy - Facial IPL treatment, within 12 months prior to screening - Any thermal treatment of the eyelids, including Lipiflow, within 6 months prior to screening - Expression of the meibomian glands, within 6 months prior to screening - In either eye, moderate to severe (Grade 3-4 on the EFRON scale) inflammation of the conjunctiva, including: allergic, vernal or giant papillary conjunctivitis - In either eye, severe (Grade 4 on the EFRON scale) inflammation of the eyelid, including: blepharochalasis, staphylococcal blepharitis or seborrheic blepharitis - Ocular surface abnormality that may compromise corneal integrity in either eye (e.g., prior chemical burn, recurrent corneal erosion, corneal epithelial defect, Grade 3 corneal fluorescein staining, or map dot fingerprint dystrophy) - Eyelid abnormalities that affect lid function in either eye, including: entropion, ectropion, tumor, edema, blepharospasm, lagophthalmos, severe trichiasis, and severe ptosis - Any systemic condition that may cause dry eye disease, including: Stevens-Johnson syndrome, vitamin A deficiency, rheumatoid arthritis, Wegener’s granulomatosis, sarcoidosis, leukemia, Riley-Day syndrome, systemic lupus erythematosus, and Sjögren's syndrome - Unwilling or unable to abstain from the use of medications known to cause dryness (e.g., isotretinoin, antihistamines) throughout the study duration. Subjects must discontinue these medications for at least 1 month prior to the baseline visit.   Any condition revealed whereby the investigator deems the subject inappropriate for this study | |
| **Objectives** | **Primary objectives** TBUT estimated at the single FU (Between 4 weeks -3 days and 4 weeks + 7 days after the final treatment)  Measurement of TBUT will be implemented using FUL-GLO® fluorescein ophthalmic strips. Three successive readings will be taken and averaged to a single value.  **Secondary objectives** Self-evaluation of OSDI at the single FU, using the OSDI questionnaire Self-evaluation of Eye Dryness Score (EDS) at the single FU, using a VAS | |
| **Criteria for Evaluation** | **Primary endpoint:**  The difference in the change of TBUT from BL to FU, between eyes in the study arm and eyes in the control arm  The improvement of TBUT in eyes of the study arm is larger than the improvement of TBUT in eyes of the control arm, where improvement is defined as a positive change of TBUT from BL to FU  **Secondary endpoints**  The difference in the change of OSDI from BL to FU, between subjects in the study arm and subjects in the control arm  The difference in the change of EDS from BL to FU, between subjects in the study arm and subjects in the control arm  The improvement of OSDI in the study arm is larger than the improvement of OSDI in the control arm, where improvement is defined as a negative change of OSDI from BL to FU  The improvement of EDS in the study arm is larger than the improvement of EDS in the control arm , where improvement is defined as a negative change of EDS from BL to the FU | |
| **Exploratory effectiveness endpoints** | - The difference in the proportion of eyes with normal TBUT (TBUT >10 sec) at FU, between eyes in the study arm and eyes in the control arm - The difference in the proportion of subjects with normal OSDI (OSDI < 23) at FU, between subjects in the study arm and subjects in the control arm - The difference in eyelids appearance, as qualitatively evaluated by the study investigator, between eyes in the study arm and eyes in the control arm - The difference in the percentage of area loss of meibomian glands, as evaluated using meibography, between eyes in the study arm and eyes in the control arm - The difference in the change of MGS from BL to FU, between eyes in the study arm and eyes in the control arm | |
| **Safety Endpoints** | Throughout the study:   - the difference in the incidence of ocular adverse events, between subjects in the study arm and subjects in the control arm - the difference in the incidence of non-ocular adverse events, between subjects in the study arm and subjects in the control arm - the difference in the incidence of unanticipated serious adverse events, between subjects in the study arm and subjects in the control arm   At the treatment sessions-   - the difference in the change of bio-microscopy examinations pre- and post- treatment, between subjects in the study arm and subjects in the control arm - the difference in the self-assessment of pain/discomfort during IPL administration, between subjects in the study arm and subjects in the control arm - the difference in the self-assessment of pain/discomfort during MGX, between subjects in the study arm and subjects in the control arm | |
| **Screening and baseline (BL) visit** | Subjects will sign the IC form within one week of passing an informed consent process and having been provided the informed consent form (ICF). After signing the ICF, subjects will undergo the following baseline (BL) assessments, in the specified order:   1. Three consecutive measurements of tear break-up time (TBUT) in both eyes 2. Routine ophthalmology tests in both eyes, including: biomicroscopy, best-corrected visual acuity (BCVA) and intra-ocular pressure (IOP) 3. Ocular Surface Disease Index (OSDI), self-assessed using an OSDI questionnaire 4. Eye dryness score (EDS), self-assessed using a visual analog scale (VAS) 5. High resolution close-up photos of both eyes, including: the iris, lid margins and eyelashes 6. Meibography of the upper and lower eyelids in both eyes 7. Meibomian gland secretion (MGS) score in 15 glands of the lower eyelid in both eyes | |
| **Randomization** | - Eligible subjects will be randomized 1:1 to a study arm or to a control arm - The randomization process will adopt a blocked randomization strategy, using random block sizes of 2 and 4 | |
| **Treatment schedule** | Four (4) treatment sessions, 2 weeks (-3 days, + 7 days) apart.  Each treatment session will consist of the following procedures, in the specified order:   1. Subject’s report of the daily usage (frequency and dose) of eye drops, warm compresses and lid hygiene since the previous visit 2. Pre-treatment biomicroscopy with the slit lamp (observation of lid margins, eyelashes, conjunctiva) 3. **In the study arm**:   Active IPL administered on the malar region (from tragus to tragus, below the lower eyelids, including the nose)  **In the control arm**:  Sham IPL administered on the same facial areas   1. Meibomian gland expression (MGX) of the upper and lower eyelids in both eyes 2. Post-treatment biomicroscopy with the slit lamp 3. Self-assessment of pain/discomfort during IPL administration, using a VAS 4. Self-assessment of pain/discomfort during MGX, using a VAS | |
| **Follow-up (FU) visit** | There will be a single follow-up (FU) visit, which will occur 4 weeks (-3 days, + 7 days) after the final treatment session. Subjects will undergo the following assessments, in the specified order:   1. Subject’s report of the daily usage (frequency and dose) of eye drop, warm compresses and lid hygiene since the previous visit 2. Three consecutive measurements of TBUT in both eyes 3. Routine ophthalmology tests in both eyes, including: biomicroscopy, BCVA and IOP 4. OSDI, self-assessed using an OSDI questionnaire 5. EDS, self-assessed using a VAS 6. High resolution close-up photos of both eyes, including: iris, lid margins and eyelashes 7. Meibography of the upper and lower eyelids in both eyes 8. Meibomian gland secretion (MGS) score in 15 glands of the lower eyelid in both eyes | |
| **Safety outcome measures** | - incidence of non-ocular adverse events throughout the study - incidence of ocular adverse events throughout the study - incidence of unanticipated serious adverse events throughout the study - self-assessment of pain/discomfort of IPL in treatment sessions (using a VAS) - self-assessment of pain/discomfort of MGX in treatment sessions (using a VAS) | |
| **Covariates** | - Baseline TBUT (continuous) - Baseline skin rosacea (4-point scale) | |
| **Sample Size** | The following assumptions were used to calculate the original sample size:   1. In the study arm, the change of TBUT from BL to FU ≈ 5 ± 5 sec 2. In the control arm, the change of TBUT from BL to FU ≈ 1 ± 5 sec 3. A type I error of 0.05 (two-tailed test) 4. A type II error of 0.2 (power = 0.8) 5. A 1:1 ratio of Treatment to Control   With these assumptions, the minimal sample size was calculated to be 50 evaluable subjects (100 evaluable eyes): 25 subjects (50 eyes) in the control arm and 25 subjects (50 eyes) in the study arm.  Assuming a dropout rate of 15%, the anticipated number of enrolled subjects was 59. Assuming a screening failure rate of 15%, the anticipated total number of screened subjects was 69.  After study initiation (but before any data were unmasked) it was found that 8 IPL patients at one site were not treated in accordance with the protocol. Accordingly, patients were added to the study to restore the original power and to allow comparison before/after corrective actions were taken. The final sample size is 83 evaluable subjects (166 evaluable eyes).Based on an updated dropout rate of 7%, the anticipated number of enrolled subjects is 89. Based on an updated screening rate of 23%, the anticipated number of screened subjects is 116. | |
| **Number of sites** | Three (3)-Four (4) | |

Table of Contents

[1. Introduction 14](#_Toc517801184)

[1.1. Background 14](#_Toc517801185)

[*1.1.1.* *Meibomian Gland Dysfunction (MGD): prevalence and current management* 14](#_Toc517801186)

[*1.1.2.* *Preferred practice pattern for management of MGD* 14](#_Toc517801187)

[*1.1.3.* *Use of intense pulsed light (IPL) for treatment of rosacea* 15](#_Toc517801188)

[*1.1.4.* *Clinical evidences for the effectiveness of IPL in the treatment of MGD* 15](#_Toc517801189)

[1.2. Rationale of the Study 16](#_Toc517801190)

[2. The Investigational Device 16](#_Toc517801191)

[2.1. Summary description of the investigational device 16](#_Toc517801192)

[*2.1.1.* *General* 16](#_Toc517801193)

[*2.1.2.* *IPL treatment head* 17](#_Toc517801194)

[*2.1.3.* *Filters* 18](#_Toc517801195)

[*2.1.4.* *Lightguides* 18](#_Toc517801196)

[*2.1.5.* *Chiller tip* 19](#_Toc517801197)

[*2.1.6.* *Controls* 19](#_Toc517801198)

[2.2. Intended use 19](#_Toc517801199)

[2.3. Intended purpose in the clinical investigation 19](#_Toc517801200)

[2.4. Training 20](#_Toc517801201)

[2.5. Procedures involved in the use of the investigational device (study arm) 20](#_Toc517801202)

[*2.5.1.* *Cleaning* 20](#_Toc517801203)

[*2.5.2.* *Topical anesthesia* 20](#_Toc517801204)

[*2.5.3.* *Coupling gel* 20](#_Toc517801205)

[*2.5.4.* *Eye protection* 20](#_Toc517801206)

[*2.5.5.* *Determination/confirmation of skin type* 20](#_Toc517801207)

[*2.5.6.* *Settings and adjustment of IPL parameters* 21](#_Toc517801208)

[*2.5.7.* *Treatment area* 24](#_Toc517801209)

[2.6. Comparator device (control arm) 25](#_Toc517801210)

[3. Study Design 25](#_Toc517801211)

[3.1. Detailed study Design 25](#_Toc517801212)

[*3.1.1.* *Randomization* 26](#_Toc517801213)

[3.2. Subject Withdrawal and Replacement 26](#_Toc517801214)

[3.3. Study Visits 26](#_Toc517801215)

[*3.3.1.* *Screening and Baseline Assessments* 27](#_Toc517801216)

[*3.3.2.* *Treatment visits* 28](#_Toc517801217)

[*3.3.3.* *Follow Up visit* 28](#_Toc517801218)

[3.4. Study Objectives 29](#_Toc517801219)

[*3.4.1.* *Primary objective* 29](#_Toc517801220)

[*3.4.2.* *Secondary Objectives* 29](#_Toc517801221)

[3.5. Study Endpoint 29](#_Toc517801222)

[*3.5.1.* *Primary efficiency endpoint* 29](#_Toc517801223)

[*3.5.2.* *Success criterion* 29](#_Toc517801224)

[*3.5.3.* *Secondary efficiency endpoints* 30](#_Toc517801225)

[*3.5.4.* *Exploratory efficiency endpoints* 30](#_Toc517801226)

[*3.5.5.* *Safety endpoints* 30](#_Toc517801227)

[*3.6.* *Study duration* 31](#_Toc517801228)

[3.7. Schedule of Times and Events 31](#_Toc517801229)

[3.8. Concurrent Control 33](#_Toc517801230)

[3.9. Blinding 33](#_Toc517801231)

[3.10. Interim analysis 33](#_Toc517801232)

[4. Study Population and Subject Selection 33](#_Toc517801233)

[4.1. Source and Sample Size 33](#_Toc517801234)

[4.2. Eligibility 34](#_Toc517801235)

[*4.2.1.* *Inclusion Criteria* 34](#_Toc517801236)

[*4.2.2.* *Exclusion Criteria* 34](#_Toc517801237)

[4.3. Subject Compensation 36](#_Toc517801238)

[*4.3.1.* *Visits* 36](#_Toc517801239)

[*4.3.2.* *Travel expenses* 36](#_Toc517801240)

[5. Study Procedures 37](#_Toc517801241)

[5.1. Screening visit 38](#_Toc517801242)

[*5.1.1.* *Subject Identification* 38](#_Toc517801243)

[*5.1.2.* *General examination* 38](#_Toc517801244)

[*5.1.3.* *Fitzpatrick* skin type 38](#_Toc517801245)

[*5.1.4.* *Informed consent* 39](#_Toc517801246)

[*5.1.5.* *Self-Assessment of Symptoms* 39](#_Toc517801247)

[*5.1.6.* *Assessment of TBUT* 40](#_Toc517801248)

[*5.1.7.* *Routine ophthalmic examination* 40](#_Toc517801249)

[*5.1.8.* *Assessment of MGS score* 41](#_Toc517801250)

[*5.1.9.* *Close-up photos of the eyes* 41](#_Toc517801251)

[*5.1.10.* *Meibography* 42](#_Toc517801252)

[*5.1.11.* *Enrollment* 43](#_Toc517801269)

[*5.1.12.* *Randomization* 43](#_Toc517801270)

[5.2. Treatment visits 44](#_Toc517801271)

[*5.2.1.* *Pre‑Treatment Procedures* 44](#_Toc517801272)

[*5.2.2.* *Treatment* 45](#_Toc517801273)

[*5.2.3.* *Post-treatment Instructions* 46](#_Toc517801274)

[*5.2.4.* *Check-up call* 46](#_Toc517801275)

[5.3. Follow‑up Visit 46](#_Toc517801276)

[*5.3.1.* *Schedule* 46](#_Toc517801277)

[*5.3.2.* *Examiner* 47](#_Toc517801278)

[*5.3.3.* *General examination and report of adverse events* 47](#_Toc517801279)

[*5.3.4.* *Assessment of symptoms* 47](#_Toc517801280)

[*5.3.5.* *Assessment of TBUT* 47](#_Toc517801281)

[*5.3.6.* *Routine ophthalmic examinations* 47](#_Toc517801282)

[*5.3.7.* *Close-up photos of the eyes* 48](#_Toc517801283)

[*5.3.8.* *Meibography* 48](#_Toc517801284)

[*5.3.9.* *MGS score* 48](#_Toc517801285)

[5.4. Termination visit 49](#_Toc517801286)

[6. Study Evaluations 49](#_Toc517801287)

[6.1. Efficiency 49](#_Toc517801288)

[*6.1.1.* *Primary effectiveness endpoint* 49](#_Toc517801289)

[*6.1.2.* *Secondary effectiveness endpoints* 49](#_Toc517801290)

[*6.1.3.* *Exploratory effectiveness endpoints* 49](#_Toc517801291)

[6.2. Safety 50](#_Toc517801292)

[7. Study Analysis Plan 50](#_Toc517801293)

[7.1. Primary Study Hypothesis 50](#_Toc517801294)

[7.2. Secondary Study Hypotheses 51](#_Toc517801295)

[7.3. Sample Size 51](#_Toc517801296)

[*7.3.1.* *Original calculation of the sample size* 52](#_Toc517801297)

[*7.3.2.* *Adjusted calculation of the sample size.* 52](#_Toc517801298)

[7.4. Study analyses 52](#_Toc517801299)

[*7.4.1.* *Stratified Analysis* 53](#_Toc517801300)

[8. Adverse events 54](#_Toc517801301)

[8.1. Adverse Events Definitions 54](#_Toc517801302)

[*8.1.1.* *Severity* 55](#_Toc517801310)

[*8.1.2.* *Relationship of AE to the device or the procedure* 55](#_Toc517801311)

[*8.1.3.* *Pre-existing Conditions* 56](#_Toc517801312)

[*8.1.4.* *Diagnosis of Adverse Event* 56](#_Toc517801313)

[*8.1.5.* *Anticipated Outcome Related Adverse Events* 56](#_Toc517801314)

[*8.1.6.* *Unanticipated Adverse Device Effects* 56](#_Toc517801315)

[*8.1.7.* *Serious Adverse Events (SAEs)* 57](#_Toc517801316)

[8.2. Reporting 58](#_Toc517801317)

[*8.2.1.* *Adverse Events (AE) and Severe Adverse Events (SAE) Reporting* 58](#_Toc517801318)

[*8.2.2.* *Device Malfunctions/Medical Device Reporting* 58](#_Toc517801319)

[8.3. Risk/ Benefit Analysis 58](#_Toc517801320)

[*8.3.1.* *Risks* 58](#_Toc517801321)

[*8.3.2.* *Anticipated Benefits* 58](#_Toc517801322)

[9. Administrative Procedures 59](#_Toc517801323)

[9.1. Investigator Selection 59](#_Toc517801324)

[9.2. Institutional Review Board (IRB) Approval 59](#_Toc517801325)

[9.3. Case Report Forms/Data Collection 59](#_Toc517801326)

[9.4. Required Documentation 60](#_Toc517801327)

[9.5. Device Use/Accountability 60](#_Toc517801328)

[9.6. Training Requirements 61](#_Toc517801329)

[9.7. Modification of Protocol 61](#_Toc517801330)

[9.8. Data Retention/Archiving Data 61](#_Toc517801331)

[9.9. Study Monitoring 61](#_Toc517801332)

[9.10. Termination of Study 61](#_Toc517801333)

[9.11. Reporting Requirements 62](#_Toc517801334)

[10. References 62](#_Toc517801335)

[11. Abbreviations and Terms 66](#_Toc517801336)

[12. Revision Control 67](#_Toc517801337)

**List of Tables**

[Table 1. *Fitzpatrick skin types* 21](#_Toc517801365)

[Table 2. *Recommended* *ranges of* *IPL settings.* 23](#_Toc517801366)

[Table 3. *Schedule of times and events* 32](#_Toc517801367)

[Table 4. *Study procedures and assessments* 37](#_Toc517801368)

[Table 5. *References* 61](#_Toc517801369)

[Table 6. *List of Abbreviations* 65](#_Toc517801370)

[Table 7. *Revisions* 66](#_Toc517801371)

[Table 8. *Text modifications* 66](#_Toc517801372)

**List of Figures**

[Figure 1. *Lumenis M22 system* 17](#_Toc517801345)

[Figure 2. *Filters and lightguides of the Lumenis M22 system* 18](#_Toc517801346)

[Figure 3. *IPL settings on the touch-screen monitor of the Lumenis M22 system* 22](#_Toc517801347)

[Figure 4. *Recommended area of test spots* 22](#_Toc517801348)

[Figure 5. *Treatment area* 25](#_Toc517801349)

[Figure 6. *Schedule of visits.* 27](#_Toc517801350)

[Figure 7. *Close-up photo of the eye* 41](#_Toc517801351)

[Figure 8. *Five-grade Meiboscale* 42](#_Toc517801352)

## Introduction

## Background

### *Meibomian Gland Dysfunction (MGD): prevalence and current management*

Meibomian gland dysfunction (MGD) is one of the most frequent diseases observed in ophthalmology. It is considered the major cause of evaporative dry eye disease (DED) [1, 2], a condition which severely affects the quality of life [3]. Symptoms include burning, itching, pain, redness, blurry vision and decrease in visual acuity. It is estimated that, in the US alone, over 40 million individuals are affected by DED [4]. More than two-thirds of DED cases are caused by MGD [5].

In normal healthy eyes, moisture of the ocular surface is maintained and stabilized by the tear film, a thin multilayer structure comprising: an inner layer of mucin (a protein produced by the cornea epithelium and conjunctiva); a middle layer of aqueous substance (generated by the lacrimal gland); and an external layer of olive-oil like liquid (the meibum, secreted by the meibomian glands). Abnormal quality or quantity of the meibum may lead to evaporation of the tear film.

The meibomian glands (MGs) consists of a few dozens of glands located at the base of the eyelid margins. In MGD, the glands narrow and the viscosity of the meibum increases, often due to inflammatory processes near the ocular surface. As a result, the meibum outflow is severely reduced, leading to evaporation of the tear film. This, in turn, leads to further inflammation of the ocular surface, further obstruction of the MGs, more reduction of the meibum outflow and more evaporation of the tear film.

### *Preferred practice pattern for management of MGD*

Currently, there is no accepted standard of care for MGD. The preferred practice pattern for management of MGD includes dietary modifications, ocular lubricants with lipid-containing supplements, lid hygiene, warm compresses, intra-ductal probing, debridement scaling, and meibomian gland expression (MGX) [6, 7]. The effects of these methods is temporal and limited, as they mostly address symptoms and not the underlying cause. Topical antibiotics and various types of anti-inflammatory drugs may also be useful.

In the current study we will examine the effect of IPL followed by MGX. In MGX, meibomian glands are squeezed (by applying force on the inner and outer surfaces of the eyelid with a specially designed forceps, Q-tips, or fingers) in order to unclog obstructed glands and evacuate their content (the meibum), which in MGD is often viscous and thus causing obstruction. A limitation of this method is the discomfort experienced by the patient, especially when the meibum is viscous and not liquid as should be in healthy glands.

### *Use of intense pulsed light (IPL) for treatment of rosacea*

Pulsed dye laser is a well-established method for treatment of benign vascular lesions such as rosacea. However, one limitation of this method is that most energy is deposited in the superficial vessels. Thus, clinical success is highly dependent on vessel depth, diameter and wall thickness.

Intense Pulsed Light (IPL) treatment relies on the principle of selective photothermolysis, in which thermal damage is limited to specific targets at the cellular level. In the case of skin conditions such as erythema of rosacea or acne, this target is the oxyhemoglobin within blood cells, which circulate in capillaries near the surface of the skin. In contrast with pulsed dye laser, IPL has a wider wavelength range, a variable pulse duration and multiple split light pulses. These characteristics cause an additive heating, leading to coagulation of vessels of different diameters and depths. Delays between pulses allows the epidermis to cool down and prevent collateral damage.

The first IPL device for treatment of lower extremity telangiectasias received FDA approval in 1995. Since then, the use of IPL was FDA-approved for dozens of indications in dermatology [8].

A first report of IPL for treating *facial* dermatological conditions dates from 1996, when it was utilized to successfully treat 80 subjects with treatment resistant facial port wine stains [9]. Later that year, a small study of 14 subjects demonstrated that IPL is highly effective and safe for treatment of essential telangiectasias, without post-treatment side effects such as purpura and changes in pigmentation, which are common after laser therapy [10]. A larger study of 200 subjects with various facial vascular lesions (facial veins, telangiectasias, facial hemangiomas, rosacea and port wine stains) demonstrated that IPL is an effective and safe alternative to laser treatment [11]. These results were later supported with a study of 1,000 consecutive subjects with facial telangiectasias [12]. In this retrospective study, 90% of the subjects experienced a clearance of 75% to 100%. Adverse side effects were minimal, involving only about 7% of the treatments.

### *Clinical evidences for the effectiveness of IPL in the treatment of MGD*

In 2002, Toyos and colleagues observed that rosacea subjects treated with IPL showed improvement in symptoms of dry eye disease [13]. They hypothesized that IPL application to the malar region and nose closes the abnormal telangiectasias, thus decreasing the secretion of inflammatory substances and improving the function of meibomian glands.

Since this discovery in 2002, Toyos and his colleagues used IPL off-label to treat hundreds of rosacea subjects presenting with dry eye disease [14, 15]. A case report from 2002 demonstrated the potential of IPL technology for treatment of dry eye disease (DED) [16]. Prior to the IPL sessions, the subject reported unbearable dry eye symptoms that were unresponsive to other lines of therapy (artificial tears, punctal plugs, cyclosporine, etc.). After 4 monthly applications of IPL, there was a dramatic improvement in tear break-up time (TBUT) and in Schirmer’s test. A recent publication summarizes data collected from 78 subjects presenting with severe symptoms of dry eye. In this retrospective study, TBUT and subjective evaluation of dry eye symptoms with the OSDI questionnaire were improved in 87% and 93% of the subjects, respectively [17]. The change in TBUT reflected a shift from severe to moderate dry eye.

Recently, additional studies demonstrated the effectiveness of IPL therapy [18]. Some of these studies demonstrated that the combination of IPL and MGX was able to improve both objective signs and subjective symptoms of DED secondary to MGD [19, 20, 21, 22, 23, 24, 25]. Other studies showed that IPL alone, without meibomian expression, was effective as well: in 2 prospective, randomized, placebo-controlled studies, IPL was applied on one side of the face, while the other side received a sham treatment. Lipid layer grade and non-invasive TBUT improved significantly from baseline to post-treatment in the treated eye, but not in the control eye [26, 27]. These studies support the hypothesis that IPL has a genuine effect on MGD, rather than merely a placebo effect.

## Rationale of the Study

A number of recent prospective studies demonstrated that IPL treatment as monotherapy or in combination with MGX gave some relief to signs and symptoms of DED due to MGD. In all of these studies, the main limitation was that the study was designed as a single arm trial. Hence, at least part of the relief observed in these studies could result from a placebo effect.

The aim of the current prospective study is to examine the contribution of the IPL treatment *itself*. For the first time, the effect of IPL will be examined in a study designed as a blinded randomized controlled trial. Subjects in the study arm will be treated with IPL followed by MGX, while subjects in the control arm will be treated with sham IPL followed by MGX. A significant difference in the outcomes of the two arms will provide support for a genuine contribution of the IPL treatment. In addition, it is expected that subjects in the study arm might feel less pain during MGX, as the heat transferred from the site of IPL administration to the meibomian glands may liquefy the meibum thus facilitating expression.

## The Investigational Device

## Summary description of the investigational device


### *General*

The M22 system (Figure 1) is 510(k) cleared in the United States by the U.S. FDA for aesthetic applications (K142860). The M22 system is a multi-application, multi-technology system which comprises a system console, an operator control panel, an LCD monitor with touch-screen technology, and several treatment heads and hand pieces. The system is continuously monitored and controlled by its internal computer. Selected parameters and user information are displayed on the monitor screen. In its full configuration, the M22 system includes 4 treatment heads and handpieces:

1. ResurFX laser (1565 nm) for fractional non-ablative skin resurfacing
2. Multi-spot Nd:YAG laser (1064 nm), for the treatment of vascular lesions
3. Q-switched Nd:YAG (1064 nm) for the removal of dark tattoos and treatment of pigmented lesions
4. Intense Pulsed Light or IPL (400-1200 nm), for treating vascular and pigmented lesions, and hair removal.

The M22 system is modular. In one of its many configurations, the M22 comprises the IPL module (treatment head and handpiece) only. Hereafter, this configuration is also referred to as the M22 with Optimal Pulse Technology, or M22-OPT. In this study, the M22-OPT is used.

### *IPL treatment head*

The IPL treatment head emits light in the range of 515 -1200 nm. The light is emitted perpendicular to the handpiece axis. The tip (lightguide) is made of sapphire and provides coverage of 1.2 cm^2^ (small tip: 8 mm by 15 mm) or 5.25 cm^2^ (large tip: 15 mm by 35 mm). The lightguide is cooled by a thermo-electric cooler (hereafter referred to as chiller). A set of filters is provided, to block part(s) of the emitted spectrum.


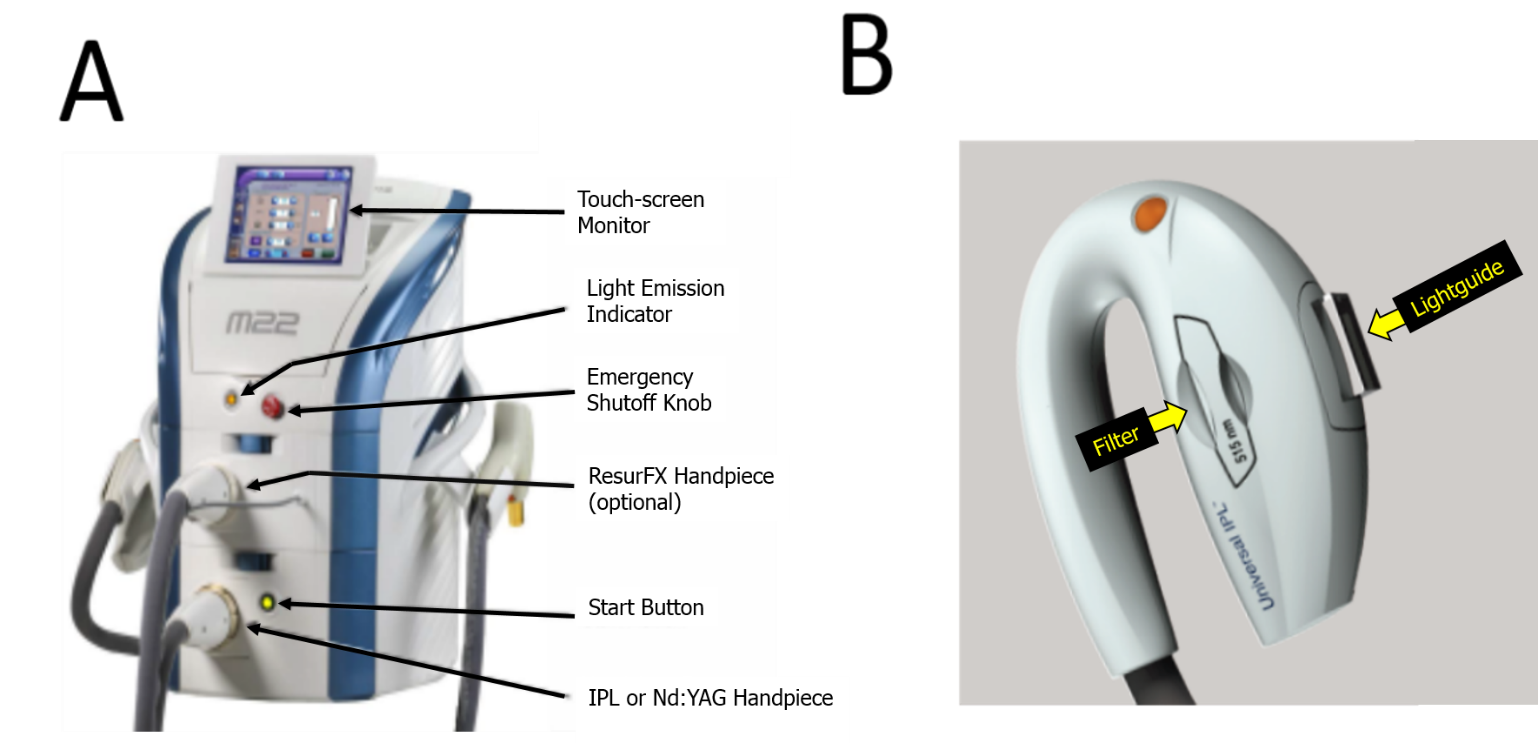


Figure 1. *Lumenis M22 system*

**A**: Multifunctional M22 platform. In its full configuration, the system has 4 handpieces: ResurFX 1565 nm, Multi-spot Nd:YAG 1064 nm, Q-switched Nd:YAG 1064 nm, and IPL. In this study, only the IPL handpiece is relevant **B**. IPL handpiece: The light energy is transmitted through a cooled sapphire lightguide. A filter blocks part of the emitted spectrum. In this example, all wavelengths below 515 nm are blocked.

### *Filters*

The IPL treatment head operates at a spectrum of 400-1200 nm. A filter, inserted in the handpiece, is used to block part of the spectrum (Figure 1B). Different types of filters are available with the M22-OPT system:

- High-pass filters, which block all wavelengths below some designated cut-off value. 7 kinds of high-pass filters are available: 515 nm, 560 nm, 590 nm, 615 nm, 640 nm, 695 nm and 755 nm.
- Notch filters, which blocking all wavelength outside of the designated ranges. 2 kinds of notch-filters are available: 530-650 & 900-1200 nm; and 400-600 & 800-1200 nm.
- An additional filter was specifically designed for the purpose of this study, to implement a sham treatment with the M22-OPT (not available commercially). This filter completely blocks all wavelengths emitted by the Xenon flash lamp. Hereafter, this filter is referred to as the “∞ filter”.

In this study, 3 types of filters are used:

1. the 560 nm or the 590 nm filter, for subjects the study arm
2. the ∞ filter, for any subject in the control arm.

### *Lightguides*

In the M22-OPT, IPL pulses are emitted perpendicular to the light bulb (located within the handpiece), through a SapphireCool^TM^ lightguide. Three types of lightguides exist: a small round lightguide (6 mm in diameter), a small rectangular lightguide (8 mm x 15 mm = 1.2 cm^2^) and a large rectangular lightguide (15 mm X 35 mm = 5.25 cm^2^) (Figure 2). In this study, only the rectangular lightguides are used.


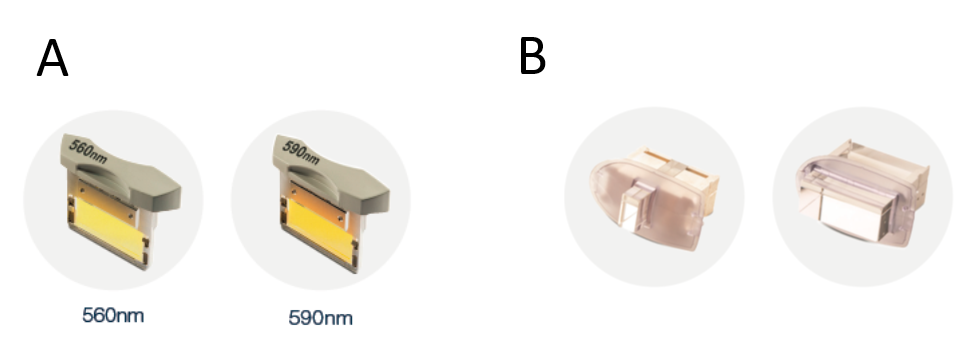


Figure 2. *Filters and lightguides of the Lumenis M22 system*

A: Two of the cut-off filters available in the M22-OPT system. Both filters are used in this study, depending on the skin type. Numbers below indicate cut-off wavelengths. B. Rectangular lightguides available in the M22-OPT: 15 mm X 8 mm (left) and 15 mm X 35 mm (right).

### *Chiller tip*

The chiller is a thermo-electric cooler component, which provides continuous cooling to the tip of the sapphire lightguide. When the tip is in contact with the skin, the chiller tip cools the epidermis and dermis under the treatment area. It is used to minimize thermal injury to non-target skin and skin structures, thus reducing possible complications. In addition, it reduces pain/discomfort that the subject may feel during IPL administration.

### *Controls*

The system controls the pulse shape and fluence using a unique Optimal Pulse Technology (OPT).

## Intended use

The Lumenis M22 system includes 4 treatment handpieces designed for aesthetic and dermatological skin procedure applications. The handpiece relevant to this study is the Intense Pulsed Light (IPL) handpiece.

The Lumenis M22 system with the IPL handpiece, hereafter also referred to as “M22 with Optimal Pulse Technology”, or “M22-OPT”, is indicated for (FDA clearance K142860):

• Benign epidermal lesions, including dyschromia, hyperpigmentation, melasma, ephelides (freckles) and tattoos

• Cutaneous lesions, including warts, scars and striae

• Benign cutaneous vascular lesions, including port wine stains, hemangiomas, facial, truncal and leg telangiectasias, erythema of rosacea, angiomas and spider angiomas, poikiloderma of Civatte, leg veins and venous malformations

• Removal of unwanted hair from all skin types, and to effect stable long term, or permanent hair reduction in skin types I-V through selective targeting of melanin in hair follicles

• Mild to moderate inflammatory Acne (*Acne vulgaris*)

## Intended purpose in the clinical investigation

In the current clinical investigation, the intended purpose is a relief in signs and symptoms of dry eye disease (DED) due to meibomian gland dysfunction (MGD). The studied population includes patients with signs and symptoms of MGD. The procedure will include administration of IPL pulses on the malar region (from tragus to tragus, below the lower eyelids, including the nose), followed by MGX.

## Training

Both the study investigators and the Sponsor, prior to any independent use of the device, will agree upon the study investigators’ training requirements. Prior to the study, the Sponsor will ensure that the study investigators have received in depth training on the use of the device. All study investigators will be trained before participation of subjects.

## Procedures involved in the use of the investigational device (study arm)

The following steps describes the procedure for administering IPL pulses using the M22-OPT:

### *Cleaning*

Before treatment, the subject’s face must be thoroughly cleaned with a mild cleansing agent, like soap, facial wipes, or a medical grade alcohol diluted by 50%.

### *Topical anesthesia*

Generally skin treatments with the M22-OPT can be performed without topical anesthesia. If topical anesthesia is used, it is not recommended to use products with vasoconstrictive effect, like EMLA. A compound gel containing Benzocaine 20% - Lidocaine 7% -Tetracaine 7% may be used.

The topical anesthetics is generally applied for a period of time (up to 1 hour) before treatment. Prior to IPL administration, the entire topical anesthetic must be completely removed and the face must be washed again.

### *Coupling gel*

The area to be treated will be coated with a thin (1-2 mm) layer of transparent coupling gel. The coupling get will be provided by the Sponsor.

### *Eye protection*

During the entire duration of IPL administration, the subject must wear two types of eye protection: First, adhesive disposable eye patches (Honeywell, Derma Aid) will be positioned over the eyelids, to cover the entire periocular area extending from the upper lid crease to the lower lid crease. On top of these, the subject will wear opaque goggles (OD ≥ 5).

In addition, during IPL administration physicians and staff present in the room must wear protective eyewear with OD = 3.

Eye protection goggles (for subjects) and glasses (for operators) will be provided by the Sponsor.

### *Determination/confirmation of skin type*

Darker skin types react more strongly to the IPL administration. Hence, before setting the IPL settings it is imperative to determine/confirm the subject’s Fitzpatrick skin type, according to Table 1 [28].

Table 1. *Fitzpatrick skin types*

| **Skin Type** | **Features** | **Reaction to UV** |
| --- | --- | --- |
| I | Very fair/ blue eyes/ freckles | Always burns, never tans |
| II | Fair/ blue, hazel or green eyes | Always/usually burns, tans with difficulty, tan fades rapidly |
| III | Cream white/fair with any eye or hair color/very common | Sometimes middle burn, always or usually tans, tan stays for weeks. |
| IV | Brown/typical Mediterranean skin/Moderately pigmented and may include Asian, Middle Eastern, Indian and Hispanic | Rarely burns, tans with ease, tan stays for months |
| V | Darker brown/darker skin type and may include Asian, Middle Eastern, Indian, Hispanic and non-Caucasian Mediterranean | Very rarely burns, tans very easily |
| VI | Darkest brown or black (non-Caucasian) | Never burns, tans very easily |

### *Settings and adjustment of IPL parameters*

IPL parameters include fluence (range: 10-32 J/cm^2^), number of sub-pulses (1-3), pulse duration (range: 3-20 msec), delay or intra-pulse duration (range: 5-150 msec), and a chiller option for cooling the lightguide tip. Note that for a single pulse, the minimal pulse duration is 4 msec, but for a double or triple pulse, the minimal sub-pulse duration is 3 msec. Settings can be viewed and modified on a touch-screen monitor (Figure 3).


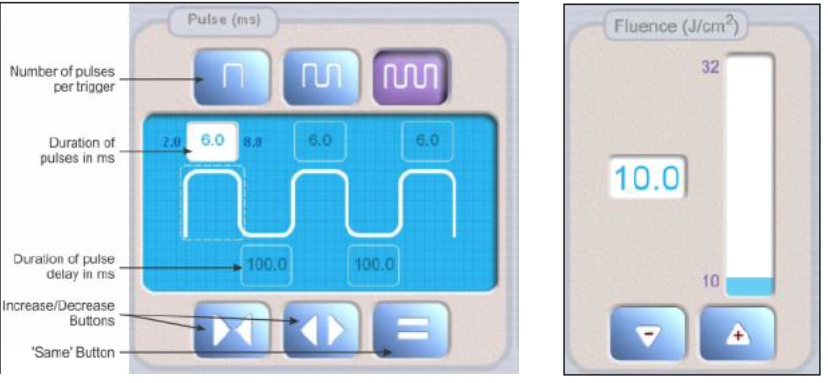


Figure 3. *IPL settings on the touch-screen monitor of the Lumenis M22 system*

Left panel: setting of Pulse characteristics- Number of sub-pulses, durations of sub-pulses and delays between sub-pulses. Right panel: setting of the total fluence. In the example, each sub-pulse exposes the treated area to a third of the total fluence (i.e., 3.33 J/cm^2^).

Prior to the first treatment (Tx1), a test spot must be performed in an inconspicuous area of similar tanning, thickness and consistency as the area to be treated (for example, the lateral side of the malar region, as illustrated in Figure 4. For the first test spot of a subject, it is recommended to use the pre-defined IPL settings for the “Skin Treatments” (ST) application, with the *Primary Condition* set to “Rosacea/Telangiectasia”, the *Lesion Depth* set to “Shallow” and the *Skin Type* set to the Fitzpatrick skin type. This action will select a set of pre-defined IPL settings (Fluence, pulse duration, pulse delay and filter) adequate for a subject with that skin type.

| 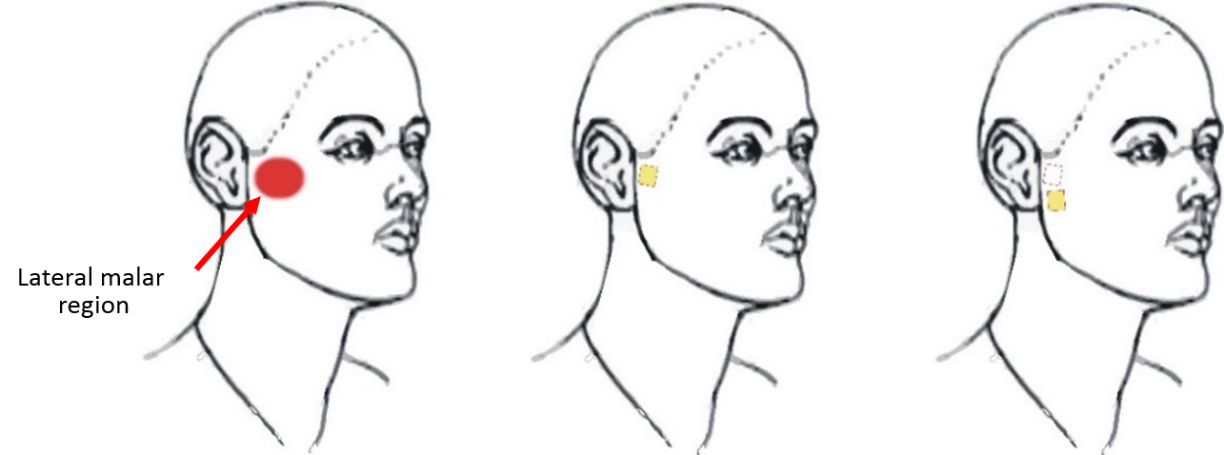  Figure 4. *Recommended area of test spots* |
| --- |

Table 2 shows the recommended IPL settings depending on the Fitzpatrick skin types. In this study, only skin types I to IV are eligible to participate.

To optimize treatment, the study investigator may adjust these pre-defined IPL settings, depending on the sensation of pain/discomfort, the immediate skin reaction, the long-term skin reaction, and the efficiency of treatment.

For the first IPL treatment, the IPL settings are based on the results of the test spots. For the 1^st^ test spot, it is advised to use the IPL settings recommended for treatment of shallow Rosacea/Telangiectasia (numbers in Table 2) and to examine the skin reaction after a few minutes. Upon decision of the study investigator, the IPL settings may be modified according to the ranges specified in Table 2. If the skin reaction to the 1^st^ test spot is excessive (such as: a break in skin integrity, blistering, sloughing and/or discoloration or bruising), or if the IPL pulse is not well-tolerated, it is recommended to gradually decrease fluence, and/or gradually increase the delay Test spots can be repeated up to 5 times.

For subsequent treatment sessions, test spots must be performed before the treatment itself. For the 1^st^ test spot, it is advised to use the IPL settings used in the previous IPL session. The study investigator may slightly modify these settings depending on the long-term skin reaction or the efficacy of the treatment. If the long-term skin response obtained after the previous treatment session is excessive (such as: a break in skin integrity, blistering, sloughing and/or discoloration or bruising) the study investigator may *decrease* the intensity of treatment by decreasing the fluence by 1 J/cm^2^, increasing the pulse duration by 1 msec and/or *increasing* the delay by 5 msec. On the other hand, if the long-term skin response is acceptable but the treatment was not effective, the study investigator may increase the intensity of treatment by *increasing* the fluence by 1 J/cm^2^, *decrease* the pulse duration by 1 msec and/or *decrease* the delay by 5 msec. As for the first treatment, test spots can be repeated several times before the IPL settings are determined and the treatment itself can start.

Whether or not the IPL settings were modified from session to session, the IPL settings used for the treatment must be recorded in the appropriate eCRF.

Table 2. *Recommended* *ranges of* *IPL settings.*

The recommended IPL settings are the numbers to the right of the inequality signs for fluence, pulse duration and pulse delay. For filters, it is recommended to use the values not in parentheses. These values are recommended but can be modified, at the discretion of the investigator. *: Only for subjects in the study arm. For subjects in the control arm, use a filter which blocks all wavelengths. This filter is labeled with a red sticker and the symbol “∞”.

| Skin type | Fluence (J/cm^2^) | Filter (nm) * | Pulse structure | Pulse duration (msec) | Delay (msec) | Chiller |
| --- | --- | --- | --- | --- | --- | --- |
| I | ≤ 20 | 560 (or 590) | Triple | ≥ 3 | ≥ 15 | On |
| II | ≤ 19 | 560 (or 590) | Triple | ≥ 3.5 | ≥ 20 | On |
| III | ≤ 18 | 560 (or 590) | Triple | ≥ 3 | ≥ 25 | On |
| IV | ≤ 17 | 590 | Triple | ≥ 3 | ≥ 30 | On |

*THIS SPACE INTENTIONALLY LEFT BLANK*

### *Treatment area*

The treatment area includes the malar region, below the lower eyelids, from tragus to tragus including the nose (Figure 5). The IPL lightguide must cover the skin above the orbital rim, about 2 mmm below the lid margin of the lower eyelid.

| 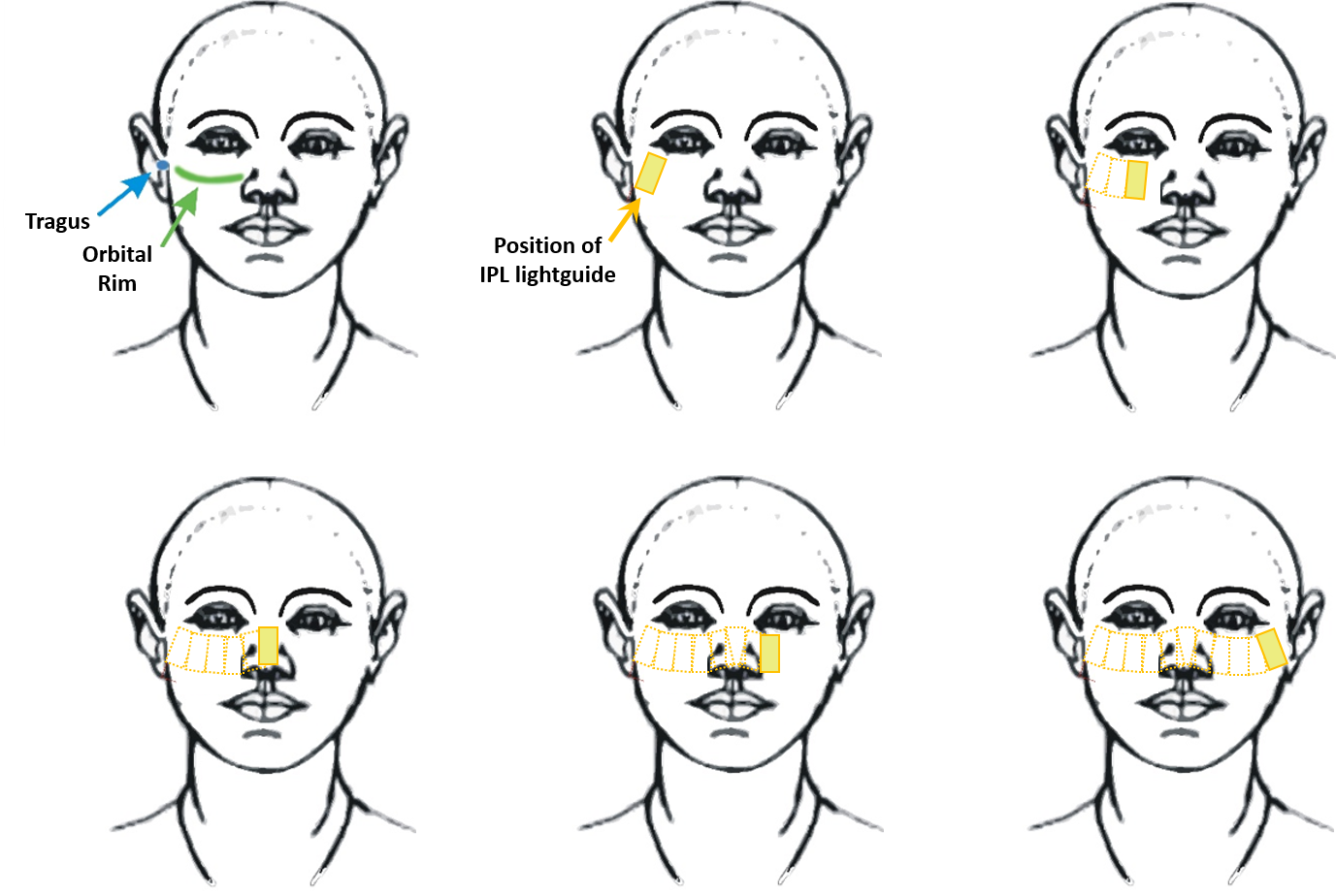  Figure 5. *Treatment area*  Read top row from left to right, and then bottom row from left to right. The IPL lightguide must cover the skin above the orbital rim (green line), about 2 mm below the lid margin of the lower eyelid. |
| --- |

## Comparator device (control arm)

Subjects in the control arm will undergo exactly the same procedures as subjects in the study arm (section ‎2.5), except that IPL treatment will be sham. The sham IPL treatment will be implemented by blocking all wavelengths emitted by the Xenon flash lamp, using the ∞ filter (see section ‎2.1.3).

## Study Design

## Detailed study Design

Prospective, multi-center, randomized, controlled, superiority, interventional, non-significant risk

### *Randomization*

Upon enrollment to the study, subjects will be randomized 1:1 to a study arm or to a control arm. The randomization process will adopt a blocked randomization strategy, using random block size of 2 and 4. Each investigational site will be provided with a set of envelopes with randomization assignments. Randomization envelopes are labeled with a unique envelope number and are to be opened in consecutive order.

## Subject Withdrawal and Replacement

In the written Informed Consent Form (ICF), the subjects will be advised that they have the right to withdraw from the study at any time without prejudice, and may be withdrawn at the Investigator’s/Lumenis’ discretion at any time. In the event that a subject drops out of the study or is withdrawn from the study, an Exit/Termination eCRF must be completed. On the withdrawal page the Investigator will record the date of the withdrawal, the person who initiated withdrawal and the reason for withdrawal.

Reasonable effort should be made to contact any subject lost to follow‑up during the course of the study, in order to complete assessments and retrieve any outstanding data and study medication/supplies. The records of subjects who terminate prior to completing the study will be retained and the reason for termination will be documented.

The following are possible reasons for subject dropout/withdrawal:

- Adverse event that would prevent subject compliance with the protocol;
- Subject withdrawal of consent;
- Subject lost to follow‑up (e.g., subject cannot be located or contacted and does not return for follow-up visits);
- Subject death;
- Investigator/Lumenis requested subject to be withdrawn.

Every effort will be made to follow the subject for the remainder of the study, even if the subject was withdrawn from the study.

## Study Visits

Each subject will undergo 4 treatment sessions at 2 weeks intervals, and a follow-up session at 4 weeks after the final treatment session. The subject can advance a treatment visit or the follow-up visit by up to 3 days, or delay it by up to 7 days. The timeline is displayed in Figure 6.

| 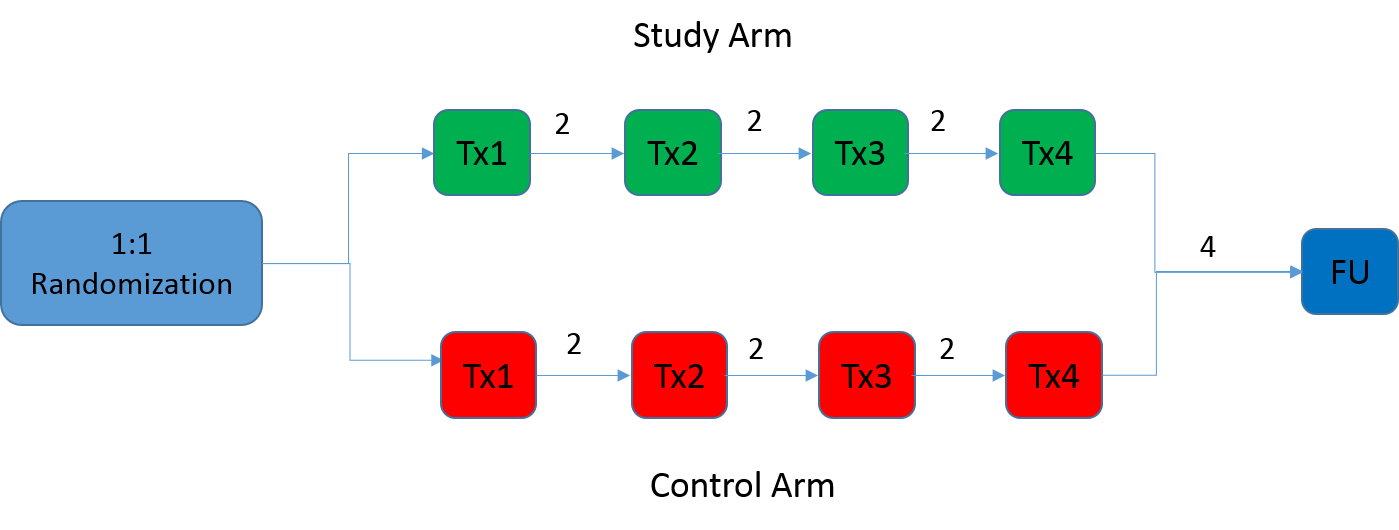  Figure 6. *Schedule of visits.*  Tx =Treatment visits; FU = Follow-up visit; Numbers above the arrows represent the time intervals (-3 days, + 7 days). In the study arm, the treatment consists of IPL administered to the malar region, followed by meibomian gland expression (Green boxes). In the control arm, the treatment consists of *sham* IPL administered to the same region, followed by meibomian gland expression (Red boxes). |
| --- |

### *Screening and Baseline Assessments*

The study investigator or a designee will start an informed consent (IC) process. The study investigator or a designee will explain that only subjects who sign the IC form are allowed to participate in this study. In the IC process, the subjects will be told that they have a 50% probability to be included in a study arm, and a 50% probability to be included in a control arm. Procedures in each of the arms will be explained to the subjects. The subject must understand that, after signing the IC form, further tests will be necessary to decide if he or she can continue in the study. The study investigator or a designee should clearly indicate that, even if the IC form is signed, the subject may not be allowed to participate in the study if the outcomes of some of the tests do not meet all inclusion and exclusion criteria, or if the immediate skin reaction to the test spots is not acceptable.

Following signing the IC form, the subject will first self-evaluate his/her symptoms using two tools: (1) the OSDI questionnaire; (2) the EDS, using a visual analog scale (0-100 VAS).

The following assessments will be performed in both eyes by a masked examiner:

- Three consecutive readings of TBUT (before any other test)
- Routine ophthalmology tests, including:
- Biomicroscopy with the slit lamp
- Best-corrected visual acuity (BCVA) with an ETDRS chart
- Intra-ocular pressure (IOP) measurement
- Meibography
- Number of non-atrophied meibomian glands in the lower eyelids
- Meibomian gland secretion score (MGS), using the method described by Lane and colleagues. [29]
- Capture of high-resolution photos of the upper and lower eyelids, including lid margins, irises and eyelashes

### *Treatment visits*

The first treatment session will be performed immediately after randomization (within one week from screening). In addition to this first treatment session, there will be 3 additional treatment sessions, at 2 weeks intervals. The subject can advance a treatment session by up to 3 days, or delay a treatment session by up to 7 days.

In total, each subject will undergo 4 treatment sessions. Each treatment session will include the following procedures, in the designated order:

1. Pre-treatment biomicroscopy with the slit lamp (observation of: lid margins, conjunctiva, and eye lashes)
2. Active or sham IPL treatment in the malar region including the nose
3. Recording of pain/discomfort during the IPL treatment using a VAS
4. Meibomian gland expression (MGX) of the lower and upper eyelids in both eyes
5. Recording of pain/discomfort during MGX using a VAS
6. Post-treatment biomicroscopy
7. Assessment of skin reaction 5-10 minutes after IPL

### *Follow Up visit*

There will be a single follow-up visit at 4 weeks after the final treatment session. The subject can advance the follow-up visit session by up to 3 days, or delay the follow-up visit by up to 7 days. At the beginning of the follow-up visit, the subject will self-evaluate his/her symptoms using two tools: (1) the OSDI questionnaire; (2) the EDS score using a VAS.

The following assessments will be performed in both eyes by a masked examiner:

:

- Three consecutive readings of TBUT (before any other test)
- Routine ophthalmology tests, including:
- Biomicroscopy with the slit lamp
- BCVA with an ETDRS chart
- IOP measurement
- Meibography
- Meibomian gland secretion score (MGS), using the method described by Lane and colleagues. [29]
- Capture of high-resolution photos of the upper and lower eyelids, including lid margins, irises and eyelashes

## Study Objectives

### *Primary objective*

The primary objective of the study is to determine the efficiency of IPL therapy, in improving TBUT in eyes with moderate to severe DED due to MGD. This objective was chosen because a reduced TBUT is one of the most common signs of DED due to MGD. TBUT is considered abnormal when shorter than 10 seconds. To include subjects with moderate to severe DED, in this study the relevant inclusion criterion is TBUT ≤ 7 seconds.

### *Secondary Objectives*

- To determine the efficiency of IPL in improving *symptoms* of DED, in subjects with DED due to MGD
- To qualitatively assess the effect of IPL on the appearance of the eyelids, , in subjects with DED due to MGD
- To determine the safety of IPL therapy

## Study Endpoint

### *Primary efficiency endpoint*

The difference in the *change* of TBUT, between eyes in the study arm and eyes in the control arm, where *change* is defined as TBUT at the follow-up minus TBUT at the baseline.

### *Success criterion*

The study will be determined as successful if it satisfies two conditions:

1. The difference in the change of TBUT, between eyes in the study arm and eyes in the control arm, is statistically significant at the α= 0.05 level.
2. The *change* of TBUT is *higher* in eyes of the study arm, compared to eyes of the control arm

, where the *change* of TBUT is defined as TBUT at the follow-up minus TBUT at the baseline.

### *Secondary efficiency endpoints*

- The difference in the *change* of OSDI, between subjects in the study arm and subjects in the control arm
- The difference in the *change* of EDS, between subjects in the study arm and subjects in the control arm

, where the *change* of the variable (OSDI or EDS) is defined as the value of the variable at the follow-up minus the value of the variable at the baseline.

### *Exploratory efficiency endpoints*

- The difference in the proportions of eyes with TBUT > 10 sec (i.e., not consistent with DED) at the follow-up, between eyes in the study arm and eyes in the control arm
- The difference in the proportions of subjects with OSDI < 23 (i.e., not consistent with DED) at the follow-up, between subjects in the study arm and subjects in the control arm
- The difference in eyelids appearance, as qualitatively evaluated by the study investigator, between eyes in the study arm and eyes in the control arm
- The difference in the *change* of MGS, between eyes in the study arm and eyes in the control arm
- The difference in the *change* of Meiboscore (Meibography), between eyes in the study arm and eyes in the control arm

### *Safety endpoints*

- The difference in the incidence of non-ocular adverse events, between subjects in the study arm and subjects in the control arm
- The difference in the incidence of ocular adverse events, between subjects in the study arm and subjects in the control arm
- The difference in the incidence of unanticipated serious adverse events, between subjects in the study arm and subjects in the control arm
- The difference in the level of pain/discomfort during IPL treatment, between subjects in the study arm and subjects in the control arm
- The difference in the level of pain/discomfort during MGX, between subjects in the study arm and subjects in the control arm
- The difference in the change of biomicroscopy immediately before and immediately after IPL treatment, between subjects in the study arm and subjects in the control arm

## *Study duration*

- For an individual participant, from the screening visit to the follow-up visit, the study duration is expected to be between 10-11 weeks (depending on whether screening and randomization/Tx1 were performed on the same day, or up to 1 week later).
- For the entire sample, from the screening of the first subject to the follow-up of the last subject, the study duration is expected to be 75 weeks. This is based on the following assumptions: a final sample size of 83 subjects (166 evaluable eyes); 3-4 sites; a screening rate of 1.5 subjects per week per site; a 23% rate of screening failures; and a lost to follow-up rate of 7% (89 enrolled subjects and 116 screened subjects)
- Enrollment will continue until at least 10 enrolled subjects have no signs of skin rosacea, even if 89 subjects were already enrolled to the study. Hence, in principle study duration can extend beyond 75 weeks.

## [Schedule of Times and Events](#_Toc342835838)

The schedule of times and events is summarized in Table 3 (see also Figure 6).

*THIS SPACE INTENTIONALLY LEFT BLANK*

Table 3. *Schedule of times and events*

| **Event** | **Time (weeks)** | **Details** |
| --- | --- | --- |
| Screening and Informed consent process | 0 | Study investigator explains the risks/benefits/procedures to the candidate |
| Informed consent signing | 0 to 1 | Subject signs the informed consent form and is enrolled |
| Assessment of baseline study outcomes | 0 to 1 | Self-assessment of symptoms with the OSDI questionnaire and EDS (VAS), and only then the following tests performed by the study investigator: routine ophthalmology tests, TBUT, MGS, and high-resolution photos of the eyelids |
| Determination of the study eye | 0 to 1 | The study eye is determined. In general, the study eye is the eye with the lower baseline TBUT. However, if both eyes have the same TBUT, the study eye is the eye with the lower baseline MGS; If both eyes have identical TBUT and identical MGS, the study eye is set randomly using a pseudo-random generator. |
| Randomization | 0 to 1 | Subject is allocated to the study arm or to the control arm |
| 1^st^ treatment | 0 to 1 | Active IPL or sham IPL, followed by MGX |
| Check-up call | 0 + 1 day to 1 week + 1 day | Phone call to be performed one day after the first treatment. The study investigator or designee will talk with the patient and ask about his/her medical condition and whether any adverse events occurred |
| 2^nd^ treatment | 2 to 3  (-3 days, +7 days) | Active IPL or sham IPL, followed by MGX |
| 3^rd^ treatment | 4 to 5  (-3 days, +7 days) | Active IPL or sham IPL, followed by MGX |
| 4^th^ treatment | 6 to 7  (-3 days, +7 days) | Active IPL or sham IPL, followed by MGX |
| Follow-up | 10 to 11  (-3 days, +7 days) | Self-assessment of symptoms with the OSDI questionnaire and EDS (VAS), and only then the following tests performed by the study investigator: routine ophthalmology tests, TBUT, and high-resolution photos of the eyelids |

## Concurrent Control

Subjects will be randomized 1:1 to a study arm and to a control arm. Subjects in the control arm will undergo exactly the same procedures as subjects in the study arm, with the exception that the IPL pulses will not be actually delivered. This applies to both test spots and the full treatment.

Due to the nature of the IPL treatment, it is not possible to ensure masking of the subjects, as subjects are generally aware that IPL treatment should be felt and should normally leave a temporary redness of the skin. This said, the following steps will be taken:

In the informed consent process, subjects will be told that in some cases the IPL treatment might be felt as a mild pain/discomfort on the skin, and that the treatment can cause redness of the skin that should disappear within a few minutes. During IPL administration, all subjects will wear protective goggles that will completely occlude their vision.

In the control arm, subjects will undergo a sham treatment that will mimic the IPL (IPL) therapy. The sham treatment will be implemented using a filter that blocks all light emitted by the M22-IPL (the ∞ filter described in section ‎2.1.3). The tip of the lightguide will still be cooled and the device will still produce clicking sounds whenever the trigger button on the IPL handpiece is triggered, but no light will be actually transmitted to the skin. Following the sham IPL procedure, subjects will undergo meibomian gland expression.

## Blinding

Every effort will be made to ensure examiners measuring primary outcomes are masked to the treatment allocation. However, due to the nature of the IPL treatment, it is not possible to completely ensure masking of the examiner. The examiner that will assess the outcome measures at the baseline visit and at the follow-up visit will not be the study investigator that will administer the IPL pulses. This will increase the probability that the examiner is masked to the allocation.

## Interim analysis

There will be no interim analysis in this study.

## Study Population and Subject Selection

## Source and Sample Size

The aim of this study is to analyze up to 83 subjects (166 eyes) who completed the full schedule, randomized 1:1 to a study arm and a control arm. It is estimated that 23% of the subjects will be screening failures, and that 7%% of the randomized subjects will drop out or will be lost to follow-up. Therefore, to reach up to 83 subjects (166 eyes) who will complete the study,116 subjects are expected to be screened and 89 subjects are expected to be enrolled.

## Eligibility

Subject eligibility is based on diagnosis of symptoms and signs compatible with DED due to MGD. Each subject will be evaluated by the study investigator according to the following criteria:

### *Inclusion Criteria*

Subjects must meet all of the following inclusion criteria to be entered into the study:

- Subject is able to read, understand and sign an Informed Consent (IC) form
- Subject is able and willing to comply with the treatment/FU schedule and requirements
- Subject is 22-85 years old
- OSDI questionnaire ≥ 23 (moderate to severe symptoms, as described in [30])
- TBUT ≤ 7 seconds in the study eye (level 2 to 5 severity, as described in [31])
- MGS of ≤ 12 in the study eye (evidence of meibomian gland obstruction along the lower eyelid, as described in [29])
- At least 5 non-atrophied meibomian glands along the lower eyelid of the study eye

, where the study eye is determined as explained in section ‎5.1.10.

### *Exclusion Criteria*

Any of the following will exclude the subject from the study:

- Fitzpatrick skin type V or VI
- Contact lens wear within the month prior to screening
- Unwilling to discontinue use of contact lenses for the duration of the study
- Ocular surgery or eyelid surgery within 6 months prior to screening
- Neuro-paralysis in the planned treatment area within 6 months prior to screening
- Other uncontrolled eye disorders affecting the ocular surface, for example active allergies
- Current use of punctal plugs
- Pre-cancerous lesions, skin cancer or pigmented lesions in the planned treatment area
- Uncontrolled infections or uncontrolled immunosuppressive diseases
- Subjects with ocular infections, within 6 months prior to screening
- Prior history of cold sores or rashes in the perioral area or in the planned treatment area that could be stimulated by light at a wavelength of 560 nm to 1200 nm, including: Herpes simplex 1 & 2, Systemic Lupus erythematosus, and porphyria
- Within 3 months prior to screening, use of photosensitive medication and/or herbs that may cause sensitivity to 560-1200 nm light exposure, including: Isotretinoin, Tetracycline, Doxycycline, and St. John’s Wort
- Over exposure to sun, within 4 weeks prior to screening
- Use of prescription eye drops for dry eye, within 7 days prior to screening, excluding artificial tears and glaucoma drops
- Radiation therapy to the head or neck, within 12 months prior to screening
- Planned radiation therapy, within 8 weeks after the last treatment session
- Treatment with chemotherapeutic agent, within 8 weeks prior to screening
- Planned chemotherapy, within 8 weeks after the last treatment session
- New topical treatments within the area to be treated, or oral therapies, within 3 months prior to screening- except over-the-counter acetaminophen-based analgesics for pain management, new oral omega 3 fatty acid supplements and topical artificial tears
- Change in dosage of any systemic medication, within 3 months prior to screening
- Anticipated relocation or extensive travel outside of the local study area preventing compliance with follow-up over the study period
- Legally blind in either eye
- History of migraines, seizures or epilepsy
- Facial IPL treatment, within 12 months prior to screening
- Any thermal treatment of the eyelids, including Lipiflow, within 6 months prior to screening
- Expression of the meibomian glands, within 6 months prior to screening
- In either eye, moderate to severe (Grade 3-4 on the EFRON scale) inflammation of the conjunctiva, including: allergic, vernal or giant papillary conjunctivitis
- In either eye, severe (Grade 4 on the EFRON scale) inflammation of the eyelid, including: blepharochalasis, staphylococcal blepharitis or seborrheic blepharitis
- Ocular surface abnormality that may compromise corneal integrity in either eye (e.g., prior chemical burn, recurrent corneal erosion, corneal epithelial defect, Grade 3 corneal fluorescein staining, or map dot fingerprint dystrophy)
- Eyelid abnormalities that affect lid function in either eye, including: entropion, ectropion, tumor, edema, blepharospasm, lagophthalmos, severe trichiasis, and severe ptosis
- Any systemic condition that may cause dry eye disease, including: Stevens-Johnson syndrome, vitamin A deficiency, rheumatoid arthritis, Wegener’s granulomatosis, sarcoidosis, leukemia, Riley-Day syndrome, systemic lupus erythematosus, and Sjögren’s syndrome
- Unwilling or unable to abstain from the use of medications known to cause dryness (e.g., isotretinoin, antihistamines) throughout the study duration. Subjects must discontinue these medications for at least 1 month prior to the baseline visit.
- Any condition revealed whereby the investigator deems the subject inappropriate for this study

## Subject Compensation

### *Visits*

Subjects will not pay for any of the office visits, examinations and procedures as part of this clinical study.

### *Travel expenses*

To cover their travel expenses, subjects will be paid $50 for each of the four treatment visits they complete, and another $50 for the follow-up visit.  Subjects who will complete the 4 treatment visits and the follow up visit that is required as part of this clinical study will receive an additional $250. In the event that the subject is discontinued early from the study, due to a significant medical event or cancellation by the Sponsor, the $250 additional payment will be paid in proportion with the number of visits that the patient completed.

The maximum amount paid to each study subject for participating in the study is $500. Payments will be given after the follow-up visit or after the subject’s last visit as part of the study, whichever comes sooner.

*THIS SPACE INTENTIONALLY LEFT BLANK*

## Study Procedures

Table 4. *Study procedures and assessments*

Tx = Treatment visit; FU = Follow-up visit

| **Activities** | **Screening** | **Enrollment / Tx1** | **Tx2** | **Tx3** | **Tx4** | **FU** |
| --- | --- | --- | --- | --- | --- | --- |
| General examination (Demographics, medical history, allergies, etc.) | 🗸 |  |  |  |  |  |
| Determination of Fitzpatrick skin type | 🗸 |  |  |  |  |  |
| Informed Consent (IC) process | 🗸 |  |  |  |  |  |
| Informed consent signing |  | 🗸 |  |  |  |  |
| Self-administration of OSDI questionnaire |  | 🗸 |  |  |  | 🗸 |
| Self-administration of EDS score (VAS) |  | 🗸 |  |  |  | 🗸 |
| Biomicroscopy with the slit lamp |  | 🗸 | 🗸 | 🗸 | 🗸 | 🗸 |
| BCVA | 🗸 |  |  |  |  |  |
| IOP | 🗸 |  |  |  |  |  |
| TBUT (3 readings) in both eyes |  | 🗸 |  |  |  | 🗸 |
| MGS score in both eyes |  | 🗸 |  |  |  | 🗸 |
| High-resolution close-up photos of both eyes |  | 🗸 |  |  |  | 🗸 |
| Meibography of the lower and upper eyelids in both eyes |  | 🗸 |  |  |  | 🗸 |
| Determination of the study eye |  | 🗸 |  |  |  |  |
| Enrollment if passed all inclusion/exclusion criteria |  | 🗸 |  |  |  |  |
| Randomization |  | 🗸 |  |  |  |  |
| Report of AE from previous treatments |  |  | 🗸 | 🗸 | 🗸 | 🗸 |
| Report of artificial eye drops and warm compresses use |  | 🗸 | 🗸 | 🗸 | 🗸 | 🗸 |
| Test spots (active or sham IPL) |  | 🗸 | 🗸 | 🗸 | 🗸 |  |
| IPL therapy (active or sham IPL) |  | 🗸 | 🗸 | 🗸 | 🗸 |  |
| Pain/discomfort of IPL using a VAS |  | 🗸 | 🗸 | 🗸 | 🗸 |  |
| MGX in both eyes |  | 🗸 | 🗸 | 🗸 | 🗸 |  |
| Pain/discomfort of MGX using a VAS |  | 🗸 | 🗸 | 🗸 | 🗸 |  |
| AE reporting and documentation |  | 🗸 | | | | |

## Screening visit

Subject identification, demographics information, past medical ocular history, known allergies, use of medications, routine ocular examinations, and determination of Fitzpatrick skin type will be performed at screening. If the study investigator determines that, based on this information, the subject is a candidate for the study, the subject will undergo an informed consent process.

If the subject signs the informed consent form within 1 week from the informed consent process, the subject will undergo the following procedures: self-administration of symptoms with the OSDI questionnaire and with a VAS (EDS score); Assessment of TBUT (before any other test done by the examiner); routine ophthalmic examinations (biomicroscopy with the slit lamp, BCVA with an ETDRS chart, IOP with tonometry); Meibography; Assessment of MGS; High resolution photos of the upper and lower lid margins.

The subject will be enrolled to the study only if he/she passes all inclusion/exclusion criteria.

All screened and enrolled subjects that sign a consent form will be recorded on the Screening/Enrollment log by site staff.

### *Subject Identification*

At the screening, the subject will receive a unique identifying Subject ID number that will be composed of the site number and a three digit consecutive subject number. This unique Subject ID will be used throughout the entire study and will be entered in the subject's eCRFs for the screening visit, for each treatment visit, and for the follow-up visit.

### *General examination*

A general clinical examination is required to assess eligibility. This examination will include demographics information, past medical ocular history, known allergies, and use of medications.

### *Fitzpatrick* skin type

The Fitzpatrick skin type will be determined according to genetic disposition, reaction to sun exposure and tanning habits [28]. The Fitzpatrick scale classifies 6 skin types, as summarized in Table 1. In this study, only Fitzpatrick skin types I-IV are eligible to participate, consistent with the device labeling.

### *Informed consent*

During the informed consent (IC) process with the subject, the study investigator will explain the purpose of the study, the possible risks and benefits, and the schedule of visits required for participation. The study investigator will explain to the subject that, after signing the IC form, the subject may not be found eligible to participate in the study if he/she doesn’t satisfy all inclusion/exclusion criteria.

The subject may take the IC form, consult with family, friends or doctor. After reading and understanding the IC, if the subject decides that he/she wishes to participate in the study, he/she should return within 1 week, with a signed IC form at which time he/she will undergo specific tests as specified below (sections ‎5.1.5 to ‎5.1.9). These tests will be done *only* after the subject signed the ICF, clearly indicating his/her understanding of the requirements and risks involved with study participation and other applicable treatment options.

### *Self-Assessment of Symptoms*

To reduce the risk of bias, symptoms will be self-evaluated by the subject *before* any of the other specific tests (TBUT, MGS, and close-up photos of the eyelids) are performed by the study investigator(s).

Self-assessment of symptoms will be performed using 2 different methods:

1. Ocular Surface Disease Index (OSDI), using a questionnaire (developed by Allergan, Inc.) questionnaire. This questionnaire, which reports a single score for both eyes, is well-established for diagnosing symptoms of DED [30].

- The questionnaire will be filled-out by the subject
- The subject will grade 12 questions related to: ocular symptoms (3 questions), vision-related function items (6 questions) and environmental triggers items (3 questions)
- Each of the 12 items will be scored from 0 to 4, where 0 indicates “never” and 4 indicates “always”
- The questionnaire should take 5 minutes to complete.
- OSDI will be calculated as the sum of the answered scores times 25, divided by the number of questions answered (range: 0 to 100).
- Based on the OSDI, a subject can be categorized as having a normal ocular surface (0-12 points), mild symptoms (13-22 points), moderate symptoms (23-32 points), or severe symptoms (33-100 points), as described in [30].
- OSDI at baseline is used for an inclusion criterion: to be included in the study, the subject must have OSDI ≥ 23 at baseline (moderate or severe symptoms).

1. Eye Dryness Score (EDS), using a VAS. This questionnaire, which reports a single score for both eyes, has been used in other dry eye studies and was recently used as the primary symptom endpoint for FDA approval of lifitegrast for the treatment of signs and symptoms of dry eye (Xiidra, manufactured by Shire US, Inc.). EDS was accepted as a clinical outcome assessment by the division of transplant and ophthalmology at fda.gov (NDA 208073, June 2015)

- The subject will be shown a 10 cm line, and will be asked to mark an X sign along the line
- The subject will be instructed to mark the X sign on the leftmost side of the line if he/she does not have any symptoms of DED, on the rightmost side of the line if he/she has intolerable symptoms of DED, or somewhere along the line at a location that best represents his/her perception of symptoms in all other cases.
- The distance between the left side of the line and the X mark will be measured. EDS (0-100) will be calculated as this distance times 10.

### *Assessment of TBUT*

TBUT will be measured for each eye separately as follows:

- A FUL-GLO^®^ fluorescein sodium ophthalmic strip (0.6 mg) will be applied to the inferior tarsal conjunctiva
- The subject will be asked to blink a few times to distribute the dye over the ocular surface.
- Once positioned at the slit lamp, the subject will close his/her eye completely.
- Then, the subject will be asked to open his/her eye without blinking.
- The timer will start as soon as the subject will open his/her eye, and it will be stopped at the first sign of breakup (first dark spot or discontinuity in the precorneal fluorescein-stained tear layer).
- For each eye, 3 consecutive readings will be taken.
- TBUT will be calculated as the average of these 3 readings.

TBUT at baseline is used as an inclusion criterion, and also to determine the identity of the study eye. To be included in the study, the study eye must have TBUT ≤ 7 seconds at baseline (severity levels 2-5, as described in [31]). The study eye will be the eye with the lower TBUT at baseline (if both eyes have the same TBUT at baseline, see section ‎5.1.10).

### *Routine ophthalmic examination*

Routine ophthalmic examinations will be performed separately for each eye, and will include: biomicroscopy with the slit lamp (examination of lid margins, meibomian gland orifices, eye lashes, conjunctiva, etc.), BCVA with an ETDRS chart, and IOP with tonometry.

### *Assessment of MGS score*

The MGS score will be estimated for each eye separately as described by Lane and colleagues [29]:

- Each gland will be graded as follows: 3 = clear liquid secretion, 2 = cloudy liquid secretion; 1 = inspissated/toothpaste consistency; 0 = no secretion.
- Along the lower eyelid, 15 glands will be evaluated: 5 nasal, 5 central and 5 temporal.
- MGS will be estimated as the sum of grades of these 15 glands (range: 0 to 45).

The MGS score at baseline will be used for an inclusion criterion: to be included in the study, the study eye must have MGS ≤ 12 at baseline.

### *Close-up photos of the eyes*

Close-up photos of both eyes will be captured with a high-resolution camera (> 325 ppi). Image resolution should be high enough to see the lid margins, the iris and individual eye lashes in focus.

For best results, the camera should be used in “Macro” mode. For a simpler camera with no macro mode (for example, iPhone 7), position the camera at a distance of 30-40 cm from the eye, and use the optic zoom to take a close-up photo. If a mobile phone camera is used, it is advised to use a second mobile phone to provide illumination, rather than rely on the flash capabilities of the camera. See an example in Figure 7 below.

*
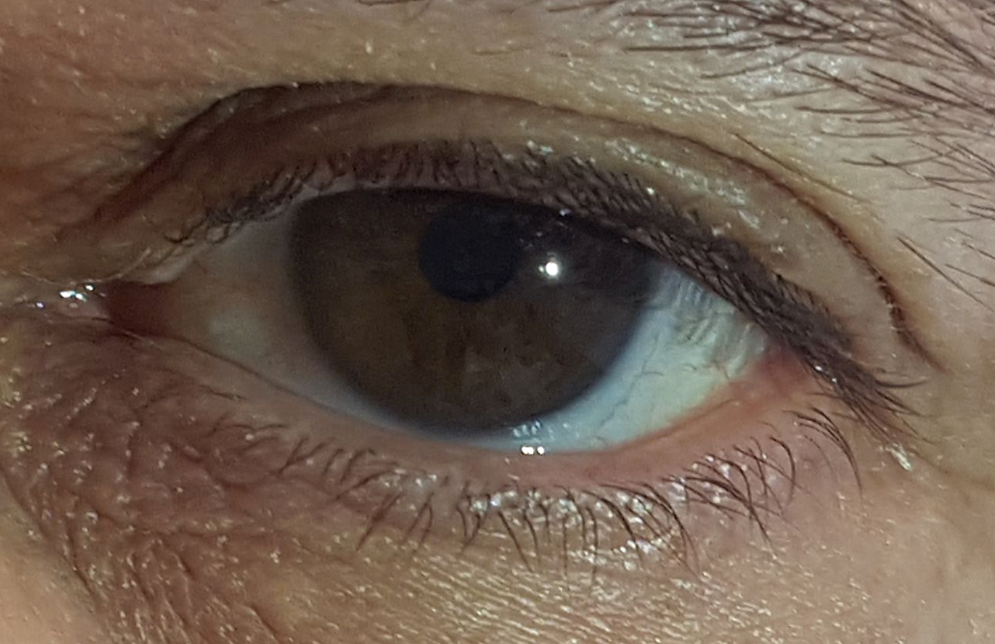
*

Figure 7. *Close-up photo of the eye*

This photo was captured with an iPhone 7. The mobile phone was positioned at a distance of 30 cm from the eye. Zooming was performed by tapping on the screen with 2 fingers and using an outward spread gesture of the fingers. A second mobile as a source of illumination (instead of the flash mode).

### *Meibography*

Meibography of the upper and lower eyelids in both eyes will be performed using an ophthalmic imaging device with a built-in infra-red camera, such as Lipiscan/Lipiview (TearScience/Johnson & Johnson), Keratograph 5M (Oculus), Antares (CSO/Lumenis) or equivalent system. While the patient rests his/her head on the chin rest, the eyelid will be gently everted (turned over) using a cotton tip applicator, and an image of the eyelid will be captured.

The percentage of area loss of the meibomian glands will be evaluated using a 5-point scale, as illustrated in Figure 8: 0: no loss; 1: < 25 % loss; 2: 26%-50% loss; 3: 51%-75% loss; 4: > 75% loss.


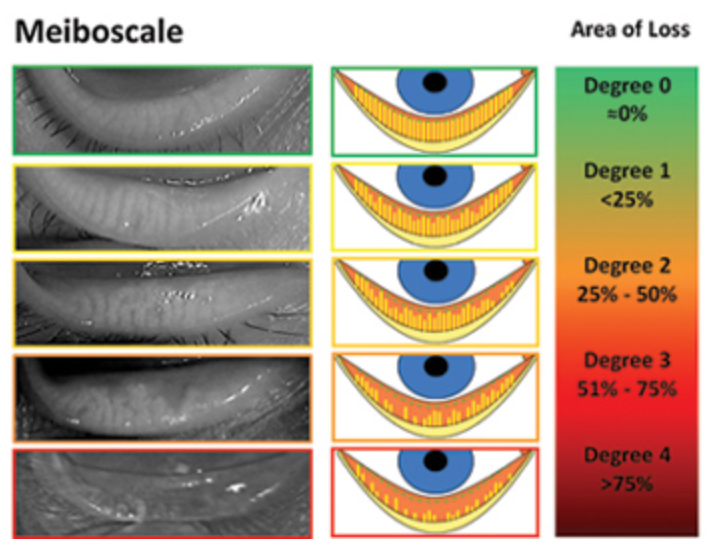


Figure 8. *Five-grade Meiboscale*


### *Enrollment*

Subjects that passed all inclusion/exclusion criteria will be enrolled to the study and then randomized to the study arm or the control arm. After randomization, treatment may start immediately. At this time, subjects in the study arm may drop from the study if they fail to pass the test spots.

### *Randomization*

Upon enrolment to the study, subjects will be randomized 1:1 to a study arm or to a control arm. The randomization process will adopt a blocked randomization strategy, using pre-defined random block sizes of 2 and 4. Each investigational site will be provided with a set of envelopes with randomization assignments. Randomization envelopes are labelled with a unique envelope number and are to be opened in consecutive order.

## Treatment visits

The schedule of treatments includes 4 treatments, at intervals of 2 weeks. The subject can advance the next treatment session by up to 3 days, or delay it by up to 7 days. The first treatment may occur on the same day as the screening visit, or up to 1 week from the screen visit. Each treatment session will include an IPL treatment or a sham IPL treatment, followed by MGX.

### *Pre‑Treatment Procedures*

- Before treatment, the subject’s face must be thoroughly cleaned with a mild cleansing agent, like soap, facial wipes, or a medical grade alcohol diluted by 50%.
- The area to be treated will be coated with a thin (1-2 mm) layer of transparent coupling gel (provided by Lumenis).
- Both eyes of the subject must be completely occluded with protective goggles. The goggles must cover the entire peri-ocular region, including the upper and lower eyelids.
- In all subjects, several test spots (active IPL or sham IPL, depending on the arm) will be given in inconspicuous areas of the facial skin, for example on the lateral aspect of the malar region. In subjects of the control arm, these sham spot tests will have no function other than to mimic the actual treatment. In subjects of the study arm, these test spots will be used to determine the IPL settings.
- For the first treatment session, the IPL settings for the 1^st^ test spot may be set using the values recommended in Table 2 , or using less intensive settings at the discretion of the investigator(s). For subsequent treatment session, the IPL settings for the 1^st^ test spot may be copied from the IPL settings used in the previous treatment session, unless the long-term skin reaction is not acceptable (including: a break in skin integrity, blistering, sloughing and/or discoloration or bruising). In this latter case the intensity of IPL settings for the 1^st^ test spot should be reduced with respect to IPL settings used in the previous IPL session, either by decreasing the fluence, increasing the pulse duration, and/or increasing the delay.
- After each test spot, the investigator will wait a few minutes and then will examine the immediate skin reaction. If the skin reaction is acceptable and as expected (pinkish to red appearance of the skin, which should gradually fade within a few minutes), the IPL treatment itself can continue with these settings. If the skin reaction is excessive (including: a break in skin integrity, blistering, sloughing and/or discoloration or bruising), or if the subject complains of more than mild pain/discomfort, the intensity of the IPL will be reduced either by decreasing the fluence, increasing the pulse duration and/or increasing the delay. Then, a new test spot will be performed.
- Up to 5 test spots are recommended. If the immediate skin reaction after these test spots is not acceptable (including: a break in skin integrity, blistering, sloughing and/or discoloration or bruising), upon decision of the investigator this subject may be discontinued from the study. It is recommended to perform up to 5 test spots.
- Whether or not the IPL settings were modified from the previous treatment session, the IPL settings used for the current treatment must be recorded in the appropriate eCRF.

### *Treatment*

- IPL pulses will be administered on the entire malar region, below the lower eyelids, from tragus to tragus including the nose. Adjacent pulses should be given with overlaps of about 1 mm.
- In subjects with skin type I-III, two passes will be performed. The first pass will be performed in one orientation of the IPL lightguide (for example, when the long side of the lightguide is parallel to the nose axis), and the second pass will be performed in the perpendicular orientation (in this example, when the short side of the lightguide is parallel to the nose axis). In subjects with skin type IV, a single pass or a double pass will be performed, at the discretion of the study investigator.
- If needed, the lightguide can be changed from small to large, for better accessibility.
- In subjects of the control arm, the IPL treatment will be sham. The study investigator will touch the skin with the lightguide of the IPL handpiece, as he/she would have done if the subject was in the study arm, but the trigger button will not be pressed. A clicking sound will be played whenever the sapphire lightguide will touch a new location on the skin, to give the subject the impression that the treatment is given.
- At the end of the IPL or sham treatment, the protective goggles will be removed.
- The subject will then assess his/her own level of pain/discomfort using a Visual Analog Scale (VAS).
- Then, each eye will be numbed with a topical solution, for example Proparacaine Hydrochloride 0.5%, and the meibomian glands of both the upper and lower eyelids will be manually expressed. Expression of the meibomian glands will be done by squeezing the meibomian glands with the aid of two Q-tips positioned on either side of the meibomian glands, or with a meibomian gland expressor forceps. Upon decision of the study investigator, the Q-tips or the meibomian gland expressor forceps may be dipped in a numbing solution as well. Squeezing the glands will be performed for 8 seconds or as soon as the glands are expressed, whichever comes first.
- Following MGX, the subject will assess his/her own level of pain/discomfort using a Visual Analog Scale (VAS).
- Up to 10 minutes later, the study investigator will assess the immediate/short term response (erythema, edema, purpura, etc.) of the skin using a 5 level scale: 0 = None, 1 = Trace, 2 = Mild, 3= Moderate; 4 = Severe.
- Longer term response (e.g. dryness) of the skin are anticipated to appear after the treatment and should disappear within several days. This will be documented in a specific section of the electronic Case Report Form (eCRF), at the next visit.

### *Post-treatment Instructions*

- At the end of each treatment visit, the study investigator or a designee will apply a sunblock protection (30-50 SPF) to the treated area.
- On the night following treatment, subjects should generally avoid hot water, cleanse their skin gently with tepid water, and hydrate the treated area with a suitable moisturizer, Aloe Vera gel, or any other anti‑burn cream or gel. It is important to avoid mechanical damage to the treated area and it should not be rubbed, scratched or picked.
- Between treatment sessions and up to 1 month after the final treatment session, subjects will be instructed to apply a sunblock protection (30-50 SPF) on the treated area, daily. The study investigator should make sure that the subject understands the need to avoid sun exposure, especially during the first 48 h after treatment.
- If the skin remains intact, subjects will be instructed to continue using standard make-up and moisturizing routine when relevant.
- In the case an unexpected skin reaction occurs, subjects will be instructed to stop using any make-up. Subjects will be instructed to immediately contact the study investigator in any case of an adverse reaction.
- Should subjects experience bothersome pain following a study treatment, they will be instructed to use an over-the-counter acetaminophen-based analgesics for pain management.

### *Check-up call*

One day after Tx1, the study investigator or designee will call the subject to ask about his/her medical condition and whether any adverse events occurred.

## Follow‑up Visit

### *Schedule*

There will be a single follow-up visit at 4 weeks (- 3 days, + 7 days) after the 4^th^ treatment visit

### *Examiner*

The examiner performing the follow-up procedures should be a different person than the study investigator who treated the subject.

### *General examination and report of adverse events*

At the beginning of the follow-up visit, the examiner will assess and record the skin condition in the treated area. The examiner will then ask the subject if any adverse event occurred. If so, the adverse events will be recorded in the appropriate AE form. In any case of SAE, the study investigator must contact Lumenis within 24 hours, to elaborate whether the SAE is related to the device (UADE).

### *Assessment of symptoms*

To reduce bias, assessment of symptoms will be performed by the subject, using the OSDI questionnaire, *before* any ocular examination or assessment of study outcome is performed by the examiner. In addition, the subject will also self-assess his/her symptoms using the EDS score with a VAS. The changes of OSDI and EDS, from baseline to the follow-up, will be used to estimate the secondary endpoints.

### *Assessment of TBUT*

TBUT will be measured for each eye separately as follows:

- A FUL-GLO^®^ fluorescein sodium ophthalmic strip (0.6 mg) will be applied to the inferior tarsal conjunctiva
- The subject will be asked to blink a few times to distribute the dye over the ocular surface.
- Once positioned at the slit lamp, the subject will close his/her eye completely.
- Then, the subject will be asked to open his/her eye without blinking.
- The timer will start as soon as the subject opens his/her eye, and it will be stopped at the first sign of tear film break of dry spot formation (first dark spot or discontinuity in the precorneal fluorescein-stained tear layer).
- For each eye, 3 consecutive readings will be taken
- TBUT will be calculated as the average of these 3 readings.

The *change* of TBUT, from baseline to the follow-up, will be used as the primary efficiency endpoint.

### *Routine ophthalmic examinations*

Routine ophthalmic examinations, performed separately for each eye, will include: biomicroscopy with the slit lamp (consisting of observation of the lid margins, conjunctiva, and eye lashes), BCVA with an ETDRS chart, and IOP with tonometry.

### *Close-up photos of the eyes*

Close-up photos of both eyes will be captured with a high-resolution camera (> 325 ppi). Image resolution should be high enough to see the lid margins, the iris and individual eye lashes in focus.

For best results, the camera should be used in “Macro” mode. For a simpler camera with no macro mode (for example. iPhone 7), position the camera at a distance of 30-40 cm from the eye, and use the optic zoom to take a close-up photo. If a mobile phone camera is used, it is advised to use a second mobile phone to provide illumination, rather than rely on the flash capabilities of the camera (for an example, see Figure 7).

### *Meibography*

Meibography of the upper and lower eyelids in both eyes will be performed using an ophthalmic imaging device with a built-in infra-red camera, such as Lipiscan/Lipiview (TearScience/Johnson & Johnson), Keratograph 5M (Oculus), Antares (CSO/Lumenis) or equivalent system. While the patient rests his/her head on the chin rest, the eyelid will be gently everted (turned over) using a cotton tip applicator, and an image of the eyelid will be captured.

The percentage of area loss of the meibomian glands will be evaluated using a 5-point scale, as illustrated in Figure 8: 0: no loss; 1: < 25 % loss; 2: 26%-50% loss; 3: 51%-75% loss; 4: > 75% loss.

### *MGS score*

The MGS score will be estimated for each eye separately as described by Lane and colleagues [29]:

- Along the lower eyelid, 15 glands will be evaluated: 5 nasal, 5 central and 5 temporal.
- Each group of 5 glands will be squeezed with Q-tips or with the Cunningham expressor (Storez) for 8 seconds or until all 5 glands are expressed, which ever comes first.
- Each gland will be graded as follows: 3 = clear liquid secretion, 2 = cloudy liquid secretion; 1 = inspissated/toothpaste consistency; 0 = no secretion.
- MGS will be estimated as the sum of grades of these 15 glands (range: 0 to 45).

## Termination visit

Normally, termination should occur at the follow-up visit after all tests were performed. In case the subject shows excessive immediate skin reaction after several test spots, early termination can occur at any of the treatment visits. The reason for early termination should be documented in the termination eCRF. The Study Exit/Termination eCRF should be signed with the study investigator’s signature.

## Study Evaluations

## Efficiency

### *Primary effectiveness endpoint*

The primary efficiency endpoint will be estimated as the difference in the *change* of TBUT from baseline to follow-up (10 weeks) between the study arm and the control arm, where the change of TBUT is defined as TBUT at the follow-up minus TBUT at the baseline.

A linear mixed-effect (LME) model will be used to test the null hypothesis that the changes of TBUT are not different between the two arms. The success criterion will include the following two conditions:

1. Two-sided p-value < 0.05
2. The change of TBUT in eyes of the study arm is *larger* than the change of TBUT in eyes of the control arm

The linear mixed-effect model will include a random effect for subjects to account for correlation between eyes within subjects.

### *Secondary effectiveness endpoints*

The following secondary efficiency endpoints will be estimated:

- The difference in the *change* of EDS, between subjects in the study arm and subjects in the control arm
- The difference in the *change* of OSDI, between subjects in the study arm and subjects in the control arm

, where *change* is defined as the value of the variable (OSDI or EDS) at the follow-up minus the value of the variable at the baseline.

### *Exploratory effectiveness endpoints*

The following exploratory endpoints will be evaluated:

- The difference in the proportions of subjects with TBUT > 10 sec at the follow-up, between eyes in the study arm and eyes in the control arm
- The difference in the proportions of subjects with OSDI < 23 at the follow-up, between subjects in the study arm and subjects in the control arm
- The difference in eyelids appearance at the follow-up, as qualitatively evaluated by the study investigator, between eyes in the study arm and eyes in the control arm
- The difference in the percentage of area loss of meibomian glands, as evaluated using meibography, between eyes in the study arm and eyes in the control arm
- The difference in the change of MGS from BL to FU, between eyes in the study arm and eyes in the control arm

, where the *change* of a variable is defined as the value of this variable at the follow-up minus its value at the baseline.

## Safety

Safety will be assessed by reporting:

- The difference in the incidence of related adverse events, between subjects in the treatment arm and subjects in the control arm
- The difference in the incidence of unrelated adverse events, between subjects in the treatment arm and subjects in the control arm
- The difference in the level of pain/discomfort during IPL treatment, between subjects in the treatment arm and subjects in the control arm
- The difference in the level of pain/discomfort during MGX, between subjects in the treatment arm and subjects in the control arm

## Study Analysis Plan

## Primary Study Hypothesis

The *change* of TBUT (ΔTBUT) in eyes of the study arm is *more positive*  than ΔTBUT in eyes of the control arm,

, where ΔTBUT is defined as TBUT at the follow-up minus TBUT at the baseline, and subscripts S, C indicate the study (S) or control (C) arms:

H0: ΔTBUT_S_ ≤ ΔTBUT_C_

H1: ΔTBUT_S_ > ΔTBUT_C_

## Secondary Study Hypotheses

1. The change of OSDI (ΔOSDI) in subjects of the study arm is *more negative*  than ΔOSDI in subjects of the control arm,

, where ΔOSDI is defined as OSDI at the follow-up minus OSDI at the baseline:

H0: ΔOSDI_S_ ≥ ΔOSDI_C_

H1: ΔOSDI_S_ < ΔOSDI_C_

1. The change of EDS (ΔEDS) in subjects of the study arm is *more negative*  than ΔEDS in subjects of the control arm,

, where ΔEDS is defined as EDS at the follow-up minus EDS at the baseline:

H0: ΔEDS_S_ ≥ ΔEDS_C_

H1: ΔEDS_S_ < ΔEDS_C_

## Sample Size

Approximately 4 months after initiation of the study, a round of site monitoring visits revealed that one of the three sites in the study was not following the instructions above with respect to where the M22 IPL lightguide should be applied; for IPL and sham subjects the lightguide was being applied too far from the eyelid margin in contrast to what is described in Figure 5 above. As a result, it is possible that the IPL treatments among the initial cohort of subjects at that site was will prove to be ineffective, or of reduced effectiveness compared to the sham control. The lightguide was applied correctly at the other two sites.

Although no data were unmasked, soon after this issue was recognized by study monitors, Lumenis initiated a corrective action (retraining and greater emphasis in the protocol), to better assure the protocol instructions would be followed.

To restore the original statistical power, the sample size was increased. Below are the original and adjusted calculations of the sample size:

### *Original calculation of the sample size*

Assuming a 1:1 randomization ratio, a mean of 5±5 sec for ΔTBUT of the study arm, a mean of 1±5 sec for ΔTBUT of the control arm, an alpha level of 0.05 and a power of 80%, the total sample size is 50 completed study eyes (25 for the study arm and 25 for the control arm). Assuming a dropout rate of 15%, the anticipated number of enrolled subjects is 58. Assuming a screening failure rate of 15%, the anticipated total number of screened subjects is 66.

In addition, MGD is less common without concomitant skin rosacea. Therefore, enrollment will continue until at least 10 subjects without skin rosacea are enrolled, even if 58 subjects were already enrolled.

### *Adjusted calculation of the sample size.*

After the corrective action to remedy the treatment errors, the sample size was recalculated using a simulation where we made the following conservative assumptions:

- The initial 8 IPL subjects at the affected site had response equal to the control arm (1±5 seconds)
- Subsequent to the corrective action, IPL subjects at the affected site had a response equal to the IPL arm (5±5 seconds)
- The final analysis was ITT, which included the inappropriately treated patients in the IPL arm.
- The correlation between eyes within a subject is 0.5.

Under these assumptions the overall treatment effect is “diluted” by the initial set of patients who were treated inappropriately. Under these conservative assumptions, the sample size needed to restore the original power was calculated to be 136 evaluable eyes (68 subjects, i.e. 18 subjects more than the original 50 subjects).

In addition, in order to allow for full comparison of the effect of the corrective action, 15 patients were added to the enrollment at the affected site so there would be sufficient before/after patients to allow for clinical evaluation of the difference.

Adding these 33 (18+15) subjects to the original 50 completed subjects, the number of completed subjects is up to 83. Therefore, with a loss to follow-up rate of 7% the expected number of enrolled subjects is 89, and with a screening failure rate of 23% the expected number of screened subjects is 116.

All sample size re-calculations were done prior to any unmasking of any results from the study.

## Study analyses

The primary efficacy outcome measure (TBUT) consist of a pre-treatment measurement (the baseline), a treatment intervention, and a post-treatment measurement (the follow-up). Both eyes will be included in the analysis. Since the two eyes of a subject are not independent, to estimate whether TBUT has improved from the baseline to the follow-up, a linear mixed effects model with random intercept, using the subject identity as the random effect, is the appropriate test. Since there are two eyes per person then there is only one possible covariance structure (i.e. symmetric, with a single correlation between eyes).

The secondary efficacy outcome measures are OSDI and EDS which will be collected at the baseline, and at a follow-up visit. In the case of OSDI, the value is estimated per subject. Hence, a single value will be collected per subject at each of these two visits. In this case a paired t-test is the appropriate statistical test to estimate whether dry eye symptoms have improved, using the change of OSDI from baseline to the follow-up. In the case of EDS, the value is estimated per eye. Hence, two values will be collected per subject at each of these two visits. Since the two eyes of a subject are not independent, to estimate whether EDS has improved from the baseline to the follow-up, a linear mixed effects model with random intercept, using the subject identity as the random effect, is the appropriate test. The level of statistical significance is α = 0.05. Non-parametric tests will be performed if the underlying distributions are not normal.

For all other eye-level analyses, where appropriate, sample means of continuous variables will be analyzed with linear mixed effects model with random intercept (whenever both eyes of a subject are included in the analysis), or two-sample t-tests. Proportions will be analyzed with $\chi$2 tests.

All statistical tests will be two-sided because potentially the treatment procedure may worsen the outcome. The level of statistical significance (type I error) is α = 0.05. Non-parametric tests will be performed if the underlying distribution is not normal.

For descriptive statistics, the N, Mean, Median, Standard deviation, Standard error of the mean, 95% confidence interval, Minimum, and Maximum will be reported for continuous variables; the frequency and proportion for each category will be reported for nominal and categorical variables.

A previous study demonstrated that in subjects with a lower TBUT at the baseline, the change in TBUT at the follow-up was larger [21]. Although not shown in this study, the severity level of skin rosacea at the baseline may also be a confounder. Hence, in addition to the primary analysis, we will carry out an analysis of covariance (ANCOVA) to adjust for these covariates.

The following baseline covariates will be included in the ANCOVA:

1. TBUT at baseline. This covariate will be treated as continuous.
2. The 4-point severity level of skin rosacea at baseline (0= none; 1 = mild; 2 = moderate; 3 = severe). This covariate will be treated as either a numeric score (continuous) or using orthogonal polynomial contrasts.

### *Stratified Analysis*

To account for the potential loss of IPL treatment effect at one site (hereafter called “A”) a stratified analysis will be performed that includes a separate stratum comprising subjects at site A who were treated prior to the corrective action. The strata will be labeled as A1, A2, B, and C for sites A, B, and C, with A1 and A2 the two separate groups of patients at site A before and after the corrective action. Since the treatment is applied over a sequence of four visits, we will conservatively assign to stratum A2 only those patients who received all four of their treatments subsequent to the corrective action. Any patient who received any one of their four scheduled treatments prior to the corrective action will be assigned to stratum A1.

Thus, the following four strata are obtained:

| Stratum | Corrective Action | Description |
| --- | --- | --- |
| A1 | No | Patients from site A with one or more treatment visits prior to the corrective action |
| A2 | Yes | Patients from site A with all 4 treatment visits subsequent to the corrective action |
| B | No | Patients from site B |
| C | No | Patients from site C |

An ANOVA analysis will be performed for the change in TBUT primary endpoint, with fixed effects for treatment and stratum and a random subject effect.

To examine the effect of the corrective action, we will group the strata into two sets: {A1}, for the pre-corrective-action subjects at site A, and {A2+B+C} for all post-corrective-action subjects at site A, combined with all subjects at B or C. We will test for significance of the corrective action by creating a 1 degree of freedom contrast for “treatment by corrective action”, using treatment (1 df) and the two corrective action sets (1 df), and test whether the contrast is significantly different from 0, using a two-sided alpha of 0.15.

- If the contrast is not significant, then the results will be analyzed as planned, pooling across all strata pre- and post- corrective action.
- If the contrast is significant then we will carry out the analyses separately for A1 and for the combined {A2+B+C}.

We will also directly compare outcomes between A1 and A2 by calculating the LSM for the difference between the two, along with the associated 95% CI.

## Adverse events

## Adverse Events Definitions

In this study, an adverse event is any undesirable clinical occurrence (sign, symptom, illness, or other medical event), that appears or worsens during the clinical study, or requires medical treatment or intervention, whether it is considered to be device related or not. If an adverse event occurs, the first concern will be the safety and welfare of the patient. Appropriate medical intervention will be made.

Any adverse events or complications reported by the subject or observed by the study investigator that occur during or after treatment with the device will be recorded in the medical record or source document and in the eCRF. The study investigator will determine if the adverse event is device related or procedure related. This assessment shall include a description of the adverse event, the onset date, the action taken (drug, surgery, hospitalization, etc.), the outcome (resolved, resolved with residuals, ongoing, etc.), the resolution date (if resolved), the severity, and seriousness.

Each adverse event should be assessed according to the following criteria:


### *Severity*

Each adverse event should be assessed for its severity, or the intensity of an event experienced by the subject.

- **Mild:** Awareness of a sign or symptom that does not interfere with the subject’s activity or is transient, resolved without treatment and has no sequelae.
- **Moderate:** May interfere with the subject’s usual activity and require additional intervention and/or treatment, and may have additional sequelae.
- **Severe:** Significant discomfort to the subject and/or interferes with the subject’s activity. Additional intervention and or treatment are necessary. Additional sequelae occur. Severe is used to describe the intensity of an event experienced by the subject.

### *Relationship of AE to the device or the procedure*

Each adverse event should be assessed for its relationship to the device or procedure as identified as follows:

**Device:** This category should be restricted to adverse events directly attributable to the study device

**Procedure:** A procedure is any activity that supports the usage of the device

Use the following categories for assigning the certainty of the relatedness:

**Definitely Related:** An AE is definitely related if it is obvious, certain or there is little doubt regarding the relationship

**Possibly Related:** An AE is possibly related if it is capable of being related but relatively unlikely.

**Not Related:** An AE is not related if it is determined that there is no plausible association.

**Unknown:** Use this term if there is insufficient information to determine if the AE is related to the device or procedure.

### *Pre-existing Conditions*

A pre-existing condition should not be reported as an adverse event unless there has been a substantial increase in severity or frequency of problems, which has not been attributed to natural history.

### *Diagnosis of Adverse Event*

There should be an attempt to report a “diagnosis” of the adverse event, rather than the individual signs associated with the diagnosis. However, a diagnosis should be reported only if, in the investigator’s judgment, it is relatively certain (i.e., definite or possible). Otherwise individual signs and symptoms should be reported as the adverse events.

### *Anticipated Outcome Related Adverse Events*

Anticipated adverse events in this study may include:

- Skin reactions including: flare-up, irritation, infection, blistering, pruritis, dryness, temporary skin color changes, burns, prolonged edema or erythema, *Herpes simplex* virus reactivation, post-inflammatory hyperpigmentation (PIH), contact dermatitis, and scarring. If any antibiotic ointment will be required during the study it will be provided by the study investigator at no cost.
- Ocular conditions including: iritis, iris atrophy, uveitis, elevated IOP, photophobia, floaters

Any anticipated AE that occurs at any time during or after the use of the study device must be reported by the study investigator to Lumenis. If the anticipated AE, in the opinion of the Sponsor (Lumenis) or the study investigator, is likely to affect the safety of the subjects or the conduct of the study, the ethical committee will be notified of the effect within 10 working days after Lumenis first receives notice of the event. If an adverse event occurs during the course of the study, the study investigator will continue following the subject until the adverse event is resolved, or up to 6 months following the last treatment, whichever comes first.

### *Unanticipated Adverse Device Effects*

An unanticipated adverse device effect as defined by the Federal Regulations [21 CFR 812.3(s)] is “any serious adverse effect on health or safety or any life-threatening problem or death caused by, or associated with, a device, if that effect, problem, or death was not previously identified in nature, severity, or degree of incidence in the investigational plan or application (including a supplementary plan or application), or any other unanticipated serious problem associated with a device that relates to the rights, safety, or welfare of subjects.” From a practical perspective, an unanticipated adverse device effect means a serious adverse event that is not listed in the device labeling, or the frequency or severity is greater than reported in the device labeling.

In the event of an unanticipated adverse event, the Investigator will immediately notify the contact person at Lumenis by telephone. If such an adverse event is being reported after normal working hours, the Investigator will leave a voice message at the telephone number below with accompanying report of the adverse event.

### *Serious Adverse Events (SAEs)*

The term “serious” is not synonymous with “severe”, the latter being used to describe the intensity of an event experienced by the subject. Any AE that does not meet any of the below criteria will be classified as non-serious. Any AE that meets one or more of the below criteria, will be classified as a serious adverse event (SAE).

An SAE is any event that satisfies one or more or the following:

- results in, or contributes to a death;
- is immediately life‑threatening (injury or illness);
- results in hospitalization, or prolongs an existing hospitalization;
- results in permanent impairment of body structure or function, or in persistent or significant disability/incapacity;
- results in an injury that requires medical intervention to prevent permanent impairment of body structure or function;
- is a device malfunction or deterioration in the characteristics and/or performance of the device that results in death or serious deterioration in health;
- is a device malfunction or deterioration in the characteristics and/or performance of the device that, if it were to occur again, could result in death or serious deterioration in health;
- results in a congenital anomaly or birth defect;
- is any medically significant injury, event or experience that requires medical/surgical intervention to prevent one of the outcomes listed above;
- results in end‑organ toxicity, including hematological, renal, cardiovascular, hepatic, gastrointestinal, and central nervous system events.

## Reporting

### *Adverse Events (AE) and Severe Adverse Events (SAE) Reporting*

All serious adverse events, whether or not deemed expected or device‑related, must be reported to the contact person at Lumenis within 24 hours by telephone (see below). A written report must follow within five (5) working days and is to include a full description of the event and sequence of events. The site personnel will directly contact the Lumenis Clinical Director ([yair.manor@lumenis.com](mailto:yair.manor@lumenis.com), +972-4-9099474, +972-52-373416).

The AE source document must be completed for all adverse events and serious adverse events. AE and SAE data must then be promptly entered into the AE eCRF for review by the Sponsor and CRO.

In addition to reporting adverse events within the context of this clinical study, 21 CFR Part 803 Medical Device reporting requirements and any applicable local device reporting requirements will be followed.

### *Device Malfunctions/Medical Device Reporting*

Each device failure will be assessed for possible reporting as a Medical Device Report (MDR), and a determination will be made in accordance with the Sponsor’s standard operating procedure. MDRs will be reported in accordance with 21 CFR 803. For all device malfunctions, notify the contact person at Lumenis within 24 hours.

## Risk/ Benefit Analysis

### *Risks*

Skin erythema or edema is anticipated and should resolve spontaneously, generally within a few hours and more rarely within a few days. More rare complications include skin flare-up, irritation, infection, blistering, pruritis, dryness, temporary skin color changes, burns, prolonged edema or erythema, *Herpes simplex* reactivation, post-inflammatory hyperpigmentation (PIH) and scarring. These complications should all resolve within a few days, possibly with topical treatment such as anti-biotic, steroids, or anti-inflammatory creams or ointments.

Use of IPL without protecting the eyes at all times with fully occluding goggles may result in severe ocular complications, including: iris photo-ablation, pupillary defects, uveitis, photophobia, pain, posterior synechiae and iris trans-illumination defects.

### *Anticipated Benefits*

For patients in the study arm, the anticipated benefit that may be observed in this study is the relief of signs and symptoms of DED, as measured by the tests and surveys required in this protocol. In addition, a previously proven benefit from IPL treatments, while not the objective of this study, is improvement in the appearance of the skin in the treated area.

Patients in the control arm may experience a smaller improvement of their signs and symptoms of DED, possibly due to a placebo effect.

## Administrative Procedures

## Investigator Selection

The investigator must be of good standing as an investigator and knowledgeable in relevant areas of clinical research to ensure adherence to the requirements of the protocol, including the protection of human subjects. Other site personnel must have appropriate research experience and infrastructure to ensure adherence to the protocol and enrollment of sufficient numbers of evaluable subjects. The curriculum vitae (CV) of the Investigator will be maintained in the Sponsor files as documentation of previous medical training, and federal databases will be searched to ensure that the investigator and/or the site are not prohibited from engaging in federally governed clinical research. The Principal Investigator will sign the signature page of this protocol, agreeing to comply with all applicable government regulations and the requirements of this study.

## Institutional Review Board (IRB) Approval

This clinical study will be conducted according to all applicable regulations under 21 CFR Part 812, and 21 CFR Parts 50 and 56, and with local laws and regulations relevant to the use of medical devices.

An Institutional Review Board (IRB) will approve the clinical study protocol prior to study initiation. Approval will be indicated in writing with reference to the final protocol number and date.
Details regarding the IRB's constitution including the names of its members, their qualifications and what function they perform on the board (e.g., chairman, specialist, lay‑member) will be made available to enable Lumenis and the Investigator to conform to regulations governing research on experimental devices.

## Case Report Forms/Data Collection

Table 5 summarizes all forms and questionnaires required to be completed for all study subjects enrolled and treated in this study. An electronic data capture (EDC) system, *IBM Clinical Development,* will be utilized to capture this information. The *IBM Clinical Development* system is a cloud-based system which is compliant with 21 CFR Part 11.

Study worksheets (source documents) will be provided by the sponsor for each subject enrolled in the study. In order to facilitate data entry, the worksheets coincide with the data entry pages in the EDC system. The appropriate worksheets will be completed by designated site personnel and initialed or signed where indicated at each examination. All worksheets will be completed in a legible manner in black/blue ink. Any corrections to the worksheets will be made by drawing a single line through the incorrect entry, recording the correct information, and initialing and dating the change.

Completed study worksheets shall be promptly entered into the EDC system by designated site staff. The study worksheets and data entered in the EDC system will be audited by the Study Monitor.

Study investigators are responsible for ensuring site staff completely and accurately records study data in the appropriate sections of the worksheets and eCRFs. All eCRFs must be signed by the Investigator in the EDC system.

The CRO will ensure completeness and data accuracy at each investigational site by comparing all study worksheets (source data) to data entered in the EDC system during periodic site visits. Recording errors will be addressed and rectified by designated site staff. Adherence to GCPs for proper recording of information as well as ensuring required corrections are made will also be addressed during these periodic visits.

All clinical data generated in the study will be submitted to Lumenis or designated CRO for quality assurance review and statistical analysis. Computerized data checks will be used to identify unusual data entries for verification prior to statistical analysis.

## Required Documentation

Prior to starting the clinical study, the following documents must be submitted or returned to Lumenis:

- Signed Clinical Trial Acknowledgement (signature in Appendix I) for the protocol (signed acknowledgement may be sent via email with original copy to follow via mail – one signed copy should be retained by the Investigator for his/her files.
- Signed Clinical Evaluation Agreement
- Curriculum vitae of the Principal Investigator
- Signed Financial Disclosure Statement for each investigator
- IRB Assurance of Compliance form or equivalent
- Written approval from the IRB of both the protocol and informed consent form

## Device Use/Accountability

The study site personnel will maintain records of the model and serial number of the device (if appropriate) used for each treatment during the conduct of the study on a Device Accountability Log. Receipt and disposition of the devices will also be maintained on the log. The device along with the associated delivery handpieces and accessories are to be maintained by the research Sponsor with reasonable care being taken by the Investigators and facility to prevent damage to, or unauthorized use of, the equipment. The device along with associated delivery handpieces and accessories are not to be used for other non‑research subjects during the conduct of the evaluation.

## Training Requirements

Both the Study Investigators and the Sponsor, prior to any independent use of the device, will agree upon the Investigators’ training requirements. Prior to the study, the Sponsor will ensure that each investigator has received in depth training on the use of the device. Device and study related training will be documented on a Training Log.

## Modification of Protocol

The protocol may be amended with the written agreement of the Sponsor and upon notification of and approval by the IRB. Investigators should review the contents of the amended protocol. Subsequent alterations should only be made in written conjunction with the Sponsor.

Medically significant amendments to the protocol (e.g., changes that increase the risk or the inconveniences for the patient, changes to the inclusion/exclusion criteria, etc.) must be approved by the IRB prior to implementation.

## Data Retention/Archiving Data

The Investigator must keep the following documents in a secure place for at least 2 years after the last clearance of a marketing application or at least 2 years have elapsed since the formal discontinuation of clinical development of the investigational product.

- A signed copy of the final protocol and amendments.
- Copies of the subjects’ study worksheets, clinical charts, all source data, any associated subject‑related raw data or, where applicable, authorized copies of raw data.
- Clinical images or photographs stored on flash drives or similar electronic media.
- The subjects’ signed Informed Consent forms.

## Study Monitoring

Lumenis and the CRO will oversee the conduct and progress of the study and will be in regular communication with the study investigators and site staff. Study monitoring will be managed by the CRO. The study monitors will be involved in inspection and review of sites and records, to ensure continued compliance with the protocol, appropriate use of the investigational device, and adequacy of the investigators and the facilities to carry out the study.

The study will be monitored by the CRO using telephone calls, email correspondence and on-site visits. At a minimum, visits to each site will be conducted at the initiation of the study, at two interim points during the study, and at termination of the study.

## Termination of Study

Lumenis reserves the right to discontinue the study at any time for administrative or other reasons. Written notice of study termination will be submitted to the investigator in advance of such termination. Termination of a specific site can occur because of (but is not limited to) inadequate data collection, low subject enrollment rate, achievement of the total enrollment, or non-compliance with the protocol or other clinical research requirements.

## Reporting Requirements

The investigator must promptly report to Lumenis any withdrawal of IRB approval at the site. Additional reporting requirements include:

- Notify Lumenis’ designee and to the IRB a report of any severe adverse device effect, whether anticipated or unanticipated, that occurs during the study as soon as possible, but in no event later than 10 working days after the investigator first learns of the effect. This report is to include a description of the effect, subsequent treatments, clinical outcomes, and outcome diagnoses. If the site personnel are not sure whether an event meets these criteria, they should call the contact person at the CRO or Lumenis.
- Notify Lumenis, the CRO, and the IRB immediately (within 24 hours) if an emergency situation arises in which the subsequent treatment, in the best interests of the subject, requires a deviation from the protocol. This should be followed with written confirmation that describes the emergency action and outcomes, to Lumenis, the CRO and the IRB within 5 working days.
- Report to the IRB and Lumenis, within 5 working days, the use of the study device without signed informed consent from the subject.
- Report adverse events in accordance with 21 CFR 803.
- Submit regular progress reports to the IRB, Lumenis or the CRO, as requested by the investigators or IRB.
- Submit a final report of the study to the IRB, the CRO and Lumenis within 3 months after termination or completion of the study.

## References

Table 5. References

| [1] | M. Lemp, L. Crews, A. Bron, G. Foulks and B. Sullivan, "Distribution of aqueous-deficient and evaporative dry eye in a clinic-based patient cohort," *Cornea,* vol. 27, pp. 1142-1147, 2008. |
| --- | --- |
| [2] | J. Nelson, J. Shimazaki, J. Benitez-del-Castillo, J. Craig, J. McCulley, S. Den and G. Foulks, "The international workshop on meibomian gland dysfunction: report of the definition and classification subcommittee," *Invest. Ophthalm. Vis. Sci.,* vol. 52, no. 4, pp. 1930-7, 2011. |
| [3] | G. Geerling, J. Tauber, C. Baudouin, E. Goto, Y. Matsumoto, T. O'Brien, M. Rolando, K. Tsubota and K. Nichols, "The international workshop on meibomian gland dysfunction: report of the subcommittee on management and treatment of meibomian gland dysfunction," *Invest. Ophthalmol. Vis. Sci.,* vol. 52, no. 4, pp. 2050-64, 2011. |
| [4] | J. Ding and D. Sullivan, "Aging and dry eye disease," *Exp. Gerontol.,* vol. 47, no. 7, pp. 483-90, 2012. |
| [5] | J. Horwath-Winter, A. Berghold, O. Schmut, I. Floegel, V. Solhdju, E. Bodner, G. Schwantzer and E. Haller-Schober, "Evaluation of the clinical course of dry eye syndrome," *Arch. Ophthalmol.,* vol. 121, no. 10, pp. 1364-8, 2003. |
| [6] | L. Jones, L. Downie, D. Korb, J. Benitez-Del-Castillo, R. Dana, S. Deng, P. Dong, G. Geerling, R. Hida, Y. Liu, K. Seo, J. Tauber, T. Wakamatsu, J. Xu, J. Wolffsohn and J. Craig, "TFOS DEWS II Management and Therapy Report," *Ocul. Surf.,* vol. 15, no. 3, pp. 575-628, 2017. |
| [7] | Cornea/External Disease Preferred Practice Pattern Panel, "Blepharitis Preferred Practice Pattern," *American Academy of Ophtalmology,* 2013. |
| [8] | C. Raulin, B. Greve and H. Grema, "IPL technology: a review," *Lasers Surg Med,* vol. 32, no. 2, pp. 78-87, 2003. |
| [9] | C. Raulin, M. Goldman, M. Weiss and R. Weiss, "Treatment of adult port-wine stains using intense pulsed light therapy (PhotoDerm VL): brief initial clinical report," *Dermatol Surg,* vol. 7, no. 594-7, p. 23, 1997. |
| [10] | C. Raulin , R. Weiss and M. Schönermark, "Treatment of essential telangiectasias with an intense pulsed light source (PhotoDerm VL)," *Dermatol Surg,* vol. 23, no. 10, pp. 941-5, 1997. |
| [11] | M. Angermeier, "Treatment of facial vascular lesions with intense pulsed light," *J Cutan Laser Ther,* vol. 1, no. 2, pp. 95-100, 1999. |
| [12] | M. Clementoni, P. Gilardino, G. Muti , M. Signorini, A. Pistorale, P. Morselli and C. Cavina, "Intense Pulsed light treatment of 1000 consecutive patients with facial vascular marks," *Aesthetic Plast Surg,* vol. 30, no. 2, pp. 226-32, 2006. |
| [13] | C. Kent, "Intense pulsed light: for treating dry eye (White Paper) [www.reviewofophthalmology.com/content/d/technology_update/i/1353/c/25857/]," *Rev Ophthalmol,* 2010. |
| [14] | H. Larkin, "Intense Pulsed Light: Skin treatment appears to clear meibomian gland (White Paper) [http://issuu.com/eurotimes/docs/16-4]," *Eurotimes (ESCRS),* vol. 16, no. 4, p. 26, 2011. |
| [15] | R. Toyos, "Intense, pulsed light for dry eye syndrome (White Paper) [http://crstoday.com/2009/04/CRST0409_14.php]," *Cat Refract Surg Today,* no. April, pp. 71-3, 2009. |
| [16] | R. Toyos, C. Buffa and S. Youngerman, "Case report: Dry–eye symptoms improve with intense pulsed light treatment (www.eyeworld.org/article.php?sid=2698)," *EyeWorld (ASCRS),* September 2005. |
| [17] | R. Toyos, W. McGill and D. Briscoe, "Intense pulsed light treatment for dry eye disease due to meibomian gland dysfunction: a 3-year retrospective study," *Photomed Laser Surg,* vol. 33, no. 1, pp. 41-6, 2015. |
| [18] | S. Dell, "Intense pulsed light for evaporative dry eye disease," *Clin Ophthalmol.,* vol. 11, pp. 1167-73, 2017. |
| [19] | G. Vora and P. Gupta, "Intense pulsed light therapy for the treatment of evaporative dry eye disease," *Curr Opin Ophthalmol,* vol. 26, pp. 314-318, 2015. |
| [20] | S. Vegunta, D. Patel and J. Shen, "Combination Therapy of Intense Pulsed Light Therapy and Meibomian Gland Expression (IPL/MGX) Can Improve Dry Eye Symptoms and Meibomian Gland Function in Patients With Refractory Dry Eye: A Retrospective Analysis," *Cornea,* vol. 35, no. 3, pp. 318-322, 2016. |
| [21] | S. Dell, R. Gaster, S. Barbarino and D. Cunningham, "Prospective evaluation of intense pulsed light efficacy and meibomian expression on relieving signs and symptoms of dry eye disease due to meibomian gland dysfunction," *Clin Ophthalmol,* vol. 11, pp. 817-827, 2017 . |
| [22] | P. Gupta, G. Vora, C. Matossian, M. Kim and S. Stinnett, "Outcomes of intense pulsed light therapy for treatment of evaporative dry eye disease," *Can J Ophthalmol.,* vol. 51, no. 4, pp. 249-53, 2016. |
| [23] | J. Albietz and K. Schmid, "Intense pulsed light treatment and meibomian gland expression for moderate to advanced meibomian gland dysfunction," *Clin Exp Optom.,* vol. doi: 10.1111/cxo.12541., 2017. |
| [24] | R. Liu, B. Rong, P. Tu, Y. Tang, W. Song, R. Toyos, M. Toyos and X. Yan, "Analysis of Cytokine Levels in Tears and Clinical Correlations After Intense Pulsed Light Treating Meibomian Gland Dysfunction.," *Am J Ophthalmol. ,* vol. 183, pp. 81-90, 2017. |
| [25] | B. Rong, P. Tu, Y. Tang, R. Liu, W. Song and X. Yan, "Evaluation of short-term effect of intense pulsed light combined with meibomian gland expression in the treatment of meibomian gland dysfunction," *Zhonghua Yan Ke Za Zhi.,* vol. 53, no. 9, pp. 675-81, 2017. |
| [26] | J. Craig, Y. Chen and P. Turnbull, "Prospective trial of intense pulsed Light for the treatment of meibomian gland dysfunction," *Invest Ophthalmol Vis Sci,* vol. 56, no. 3, pp. 1965-70, 2015. |
| [27] | X. Jiang, H. Lv, H. Song, M. Zhang, Y. Liu, X. Hu, X. Li and W. Wang, "Evaluation of the Safety and Effectiveness of Intense Pulsed Light in the Treatment of Meibomian Gland Dysfunction," *J Ophthalmol,* 2016. |
| [28] | T. Fitzpatrick, "The validity and practicality of sun-reactive skin types I through VI," *Arch Dermatol,* vol. 124, no. 6, pp. 869-71, 1988. |
| [29] | S. Lane, H. DuBiner, R. Epstein, P. Ernest, J. Greiner, D. Hardten, E. Holland, M. Lemp, J. 2. McDonald, D. Silbert, C. Blackie, C. Stevens and R. Bedi, "A new system, the LipiFlow, for the treatment of meibomian gland dysfunction," *Cornea,* vol. 31, pp. 396-404, 2012. |
| [30] | K. Miller, J. Walt, D. Mink, S. Satram-Hoang, S. Wilson, H. Perry, P. Asbell and S. Pflugfelder, "Minimal clinically important difference for the ocular surface disease index," *Arch Ophthalmol. ,* vol. 128, no. 1, pp. 94-101, 2010. |
| [31] | B. A. Tomlinson A1, D. Korb, S. Amano, J. Paugh, E. Pearce, R. Yee, N. Yokoi, R. Arita and M. Dogru, "The international workshop on meibomian gland dysfunction: report of the diagnosis subcommittee," *IOVS,* vol. 52, no. 4, pp. 2006-49, 2011. |

## Abbreviations and Terms

Table 6. *List of Abbreviations*

| **Term** | **Meaning** | **Category** |
| --- | --- | --- |
| AE | Adverse Event | Clinical term |
| BCVA | Best Corrected Visual Acuity | Ocular test |
| CRF | Case Report Form | Clinical term |
| CRO | Clinical Research Organization | Clinical term |
| CV | Curriculum vitae | General |
| DED | Dry Eye Disease | Indication |
| EC | Ethics Committee | Clinical term |
| eCRF | Electronic case report form | Clinical term |
| EDC | Electronic Data Capture | Clinical term |
| EDS | Eye Dryness Score | Score used as Outcome |
| ETDRS | Early Treatment Diabetic Retinopathy Study | Chart used for measuring visual acuity |
| FU | Follow-up | Clinical term |
| IC | Informed Consent | Clinical term |
| ICF | Informed Consent Form | Clinical term |
| IOP | Intra Ocular Pressure | Ocular test |
| IPL | Intense Pulsed Light | Procedure |
| IRB | Institutional Review Board | Clinical term |
| J/cm^2^ | Joules per centimeter square | Unit |
| LCD | Liquid Crystal Display | Technical term |
| mOsm/L | Milliosmoles per liter | Unit |
| N | Number of subjects | Statistical term |
| Nd:YAG | Neodymium-doped Yttrium Aluminum Garnet | Technical term |
| nL | Nanoliter | Unit |
| MDR | Medical Device Report | Clinical term |
| MG | Meibomian Gland | Anatomical term |
| MGS | Meibomian Gland Secretion | Score used as Inclusion |
| MGD | Meibomian Gland Dysfunction | Indication |
| MGX | Meibomian Gland Expression | Procedure |
| OPT | Optimal Pulse Technology | Technical term |
| OSDI | Ocular Surface Disease Index | Index used as Inclusion/Outcome |
| PIH | Post Inflammatory Hyperpigmentation | Indication |
| ppi | Points per Inch | Unit |
| SAE | Serious Adverse Event | Clinical term |
| TBUT | Tear Breakup Time | Value used as Inclusion/Outcome |
| Tx | Treatment | Clinical term |
| UADE | Unanticipated adverse device effect | Clinical term |
| VAS | Visual Analog Scale | Score |
| χ^2^ | Chi-square | Statistical term |
